# Digital Shade Matching in Dentistry: A Systematic Review

**DOI:** 10.3390/dj11110250

**Published:** 2023-10-27

**Authors:** Farah Rashid, Taseef Hasan Farook, James Dudley

**Affiliations:** Adelaide Dental School, The University of Adelaide, Adelaide, SA 5000, Australia; taseef.farook@adelaide.edu.au (T.H.F.); james.dudley@adelaide.edu.au (J.D.)

**Keywords:** photography, colour analysis, aesthetic dentistry, digital shade matching, metamerism

## Abstract

The pursuit of aesthetic excellence in dentistry, shaped by societal trends and digital advancements, highlights the critical role of precise shade matching in restorative procedures. Although conventional methods are prevalent, challenges such as shade guide variability and subjective interpretation necessitate a re-evaluation in the face of emerging non-proximity digital instruments. This systematic review employs PRISMA protocols and keyword-based search strategies spanning the Scopus^®^, PubMed.gov, and Web of Science^TM^ databases, with the last updated search carried out in October 2023. The study aimed to synthesise literature that identified digital non-proximity recording instruments and associated colour spaces in dentistry and compare the clinical outcomes of digital systems with spectrophotometers and conventional visual methods. Utilising predefined criteria and resolving disagreements between two reviewers through Cohen’s kappa calculator, the review assessed 85 articles, with 33 included in a PICO model for clinical comparisons. The results reveal that 42% of studies employed the CIELAB colour space. Despite the challenges in study quality, non-proximity digital instruments demonstrated more consistent clinical outcomes than visual methods, akin to spectrophotometers, emphasising their efficacy in controlled conditions. The review underscores the evolving landscape of dental shade matching, recognising technological advancements and advocating for methodological rigor in dental research.

## 1. Introduction

Pleasing dental aesthetics fuelled by social media and digital multimedia communications [1,2,3,4] have been proposed to represent greater importance than oro-dental function. It has been reported [5] that restoration with a shade mismatch causes increased patient dissatisfaction in comparison with a sub-optimal tooth shape.

Conventional visual colour matching using shade guides is a relatively simple procedure and most commonly used to match tooth shades. The aesthetic outcome of the restoration may lack predictability but may not necessarily be associated with deficiencies in the method of taking the shade. For example, when using a VITA classical A1–D4^®^ system to match a resin composite restoration, an A3 composite shade from one brand may have a markedly different shade to that manufactured by another company [6].

A contact-based instrument evaluates the colour of an object when the instrument is in contact with that surface, whereas a non-proximity device such as a digital camera records shades from a distance. Non-proximity devices that have been used to match colour shades in dentistry include professional digital cameras [7,8,9], intraoral cameras [10,11], and smartphone cameras [12,13]. Shade matching using spectrophotometers and colourimeters were first introduced as contact-based colour-measuring devices in dentistry in the 1970s [14]. A spectrophotometer measures the light energy that is reflected from a body [15], whereas a colourimeter measures reflected light and then converts it to red, green, and blue intensities [16].

The use of a spectrophotometer or colourimeter for shade taking has been referred to as the “gold standard”. However, the concept of a single and permanent “gold standard” has been challenged in that knowledge is constantly advancing, and therefore, the term is constraining [17,18]. In addition, with the application of artificial intelligence in analysing shades and colour variations, the term “ground truth” has been suggested, as it implies the best attempt to obtain the truth. In this paper, instead of accepting the concept of a single gold standard or ground truth, we adopted the concept of a “*Criterion Standard*”, which is the best standard at a given time [19].

The employment of digital methods to shade match restorations is driven partly by an ever-increasing acceptance of digitisation and automated diagnostics in dentistry—particularly the use of intraoral scanners. Digital shade matching is based on the theory of colour spaces or colour models [20]. In 1931, CIE (International Commission on Illumination) proposed various colour spaces on an XYZ tristimulus, a system for visually matching a colour against the three primary colours of red (R), green (G), and blue (B), namely, RGB. In 1976, CIE introduced the L* a* b* values (L*, where 0 is dark and 100 diffuse white; a*, where negative values denote green and positive values red; and b*; where negative values denote blue and positive values yellow) with a formula to express colour difference by calculating Euclidian colour differences, commonly referred to as Delta E (∆E) [7,21,22]. Simply, ∆E = <1.0 are colour differences that are not perceptible to the human eye, ∆E = 2–10 are colour differences perceptible at a glance, and ∆E = 100 are colours that are exactly opposite. A detailed understanding of the various colour models is not pertinent to this systematic review, although definitions, functions, mathematical commonality, and formulae are stated in File S1; Table S1.

The primary aim of this systematic review was to methodically synthesise existing literature to identify non-proximity digital instruments and colour spaces employed in dentistry. Simultaneously, the secondary objective was to aggregate studies that investigate the comparison of these instruments with spectrophotometers or conventional visual colour-matching methods.

## 2. Materials and Methods

### 2.1. Protocol

This systematic review adhered to the guidelines set forth by the Preferred Reporting Items for Systematic Reviews and Meta-Analyses (PRISMA). The protocol was officially registered with The International Prospective Register of Systematic Reviews (PROSPERO) under registration number CRD42020211418.

### 2.2. Eligibility Criteria for Systematic Review

#### 2.2.1. Inclusion Criteria

1. Articles using digital colour analyses (for example, XYZ tristimulus, RGB, CIELAB, ∆E) for the head and neck region with or without measurable outcomes;
2. Articles investigating digital shade taking used in dentistry and characterised according to the following MeSH terms [23]:
  - Colourimetry in dentistry;
  - Endodontics and dental aesthetics;
  - Prosthodontics;
  - Maxillofacial prostheses;
  - Periodontics and oral pathology;
  - Orthodontics;
3. Studies using computerised digital photography for dental and facial skin colour matching/analyses (see below Section 2.2.2, Exclusion Criteria No 4);
4. Research articles, clinical trials, clinical case reports and case series, dental techniques, and short communications describing the implementation of digital photography to carry out dental shade matching (see below Section 2.2.2, Exclusion Criteria no 5).

#### 2.2.2. Exclusion Criteria

1. Studies that used only visual methods for shade taking;
2. Studies that used spectrophotometric or colourimetric methods but did not include digital photography;
3. Articles that described only the use of dental clinical photography;
4. Studies describing only the development of cosmetic beauty products;
5. Editorials, opinions, review articles, reports on lectures, book chapters, conference proceedings, non-peer-reviewed articles, non-English articles, and patent files.

### 2.3. Information Sources

Data were retrieved from the following databases: Scopus^®^ (Elsevier, Amsterdam, The Netherlands), PubMed.gov (National Library of Medicine, Bethesda, MD, USA) and Web of Science™ (WoS) (Clarivate Analytics PLC, London, UK). The initial searches were carried out between 20 August 2020 and 12 September 2020. A search update was performed later in October 2023.

The article search process was conducted independently by two reviewers. Inter-rater reliability was measured using Cohen’s kappa (*K*) coefficient (i.e., a statistical coefficient that measures degree of agreement and disagreement between two reviewers)

### 2.4. Search Keywords

Keyword-based search strings using Boolean logic [24] were used to examine the databases. The search strings and search results are provided in File S1; Table S2.

### 2.5. Data Collection Process

**Figure 1:**
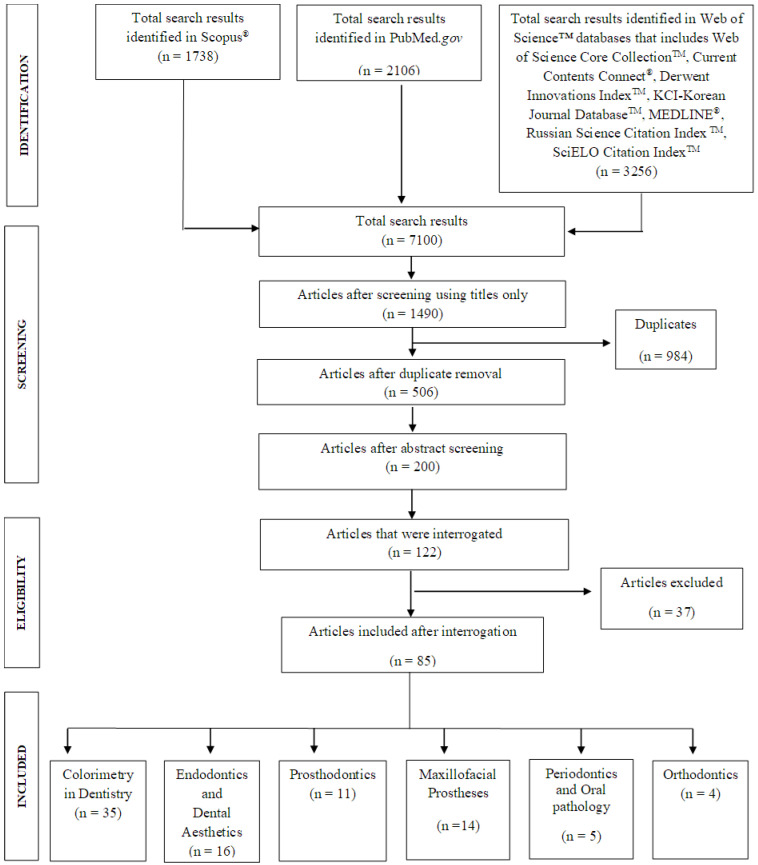
PRISMA flowchart.

Duplicate articles were removed using EndNote™ X8.2e. The abstract screening was carried out using the above a priori inclusion and exclusion criteria. All papers that were deemed to meet the inclusion criteria (n = 122) were read in full. After a thorough full paper read, 37 papers were excluded, and the reasons for exclusion of those articles are provided in File S1; Table S3. This led to 85 papers that fully satisfied the predefined inclusion criteria (see Figure 1).

### 2.6. Risk of Bias and Applicability

The quality of all included papers was assessed in the following order:

Firstly, the Joanna Briggs Institute Critical Appraisal tool (JBI) was used to assess the trustworthiness, relevance, and results of the included papers. When applying the JBI tool, each paper was ascribed a score, with less trustworthy articles scoring lower than studies that were judged robust.

Following the application of JBI, GRADEpro GDT (Guideline Development Tool) was used to assess methodological quality, qualitative rigor, and clinical relevance [25,26].

The study incorporated two different bias assessment tools to enhance the comprehensiveness of understanding potential biases, recognising that even if the outcomes are similar, the utilisation of distinct tools may uncover nuanced differences to a lesser extent [27].

### 2.7. Data Items and Summary Measures

To answer the first research question, specifically, which non-proximity digital instruments were used and their adopted colour space, the following information was collected from the 85 selected articles: study aims, study design, photographic apparatus with calibrations, study outcomes, possible sources of bias, novel approach, and potential clinical impact. These findings are summarised in File S1; Tables S5–S10.

To answer the second research question, namely, whether these non-proximity digital methods have better clinical outcomes than those taken with a spectrophotometer, colourimeter, and conventional visual methods, the following modified four-point Newcastle–Ottawa Scale [28,29] was used to grade papers that met the initial inclusion criteria (n = 85) to answer the second research question:

Scoring Criteria

**0**  Articles that did not compare non-proximity digital methods with measurements from a spectrophotometer or colourimeter or that used conventional visual methods;
**1**  Studies that did not adequately define the characteristics of the PICO comparison group;
**2**  Studies that did define group characteristics but compared only non-proximity methods with contact or conventional visual methods but without quantitative measurements;
**3**  Studies that compared non-proximity digital methods for shade matching with measurements from a spectrophotometer, colourimeter, and conventional visual methods using quantitative measurements.

Only articles that scored a 3 on the four-point Newcastle–Ottawa Scale were included for the PICO evaluation. Based on the following PICO criteria, a search was conducted:

- Population: studies reporting dental aesthetic treatment;
- Intervention: studies that used non-proximity devices for shade taking;
- Comparison: dental shades from non-proximity devices compared with the *Criterion Standards* we adopted (viz measurements from spectrophotometers and colourimeters, and conventional visual methods);
- Outcome: whether shades obtained with non-proximity devices had better clinical outcomes than those obtained with the use of spectrophotometers, colourimeters, or conventional visual methods.

### 2.8. Updated Searches

A search update was performed in October 2023. The papers identified (n = 19) were subjected to the same scrutiny as for the initial search. Although these newly added studies were tabulated separately (File S1; Table S4), their findings are incorporated into the Discussion section.

## 3. Results

### 3.1. Literature Search and Quality of the Papers

One thousand seven hundred thirty-eight articles were identified using the Scopus^®^ 2106 and PubMed.gov databases and 3256 using the Web of Science™ database. One hundred twenty-two articles met the inclusion criteria, but after interrogation, 37 further articles were excluded. See Figure 1 for the PRISMA flowchart and also File S1; Table S3.

The following supplementary tables show the article categorisation based on dental subject areas:

(1) Colourimetry in dentistry (File S1; Table S5) (n = 35) [3,4,7,8,9,10,11,12,13,15,16,21,30,31,32,33,34,35,36,37,38,39,40,41,42,43,44,45,46,47,48,49,50,51,52];
(2) Endodontics and dental aesthetics (File S1; Table S6) (n = 16) [5,53,54,55,56,57,58,59,60,61,62,63,64,65,66,67];
(3) Prosthodontics (File S1; Table S7) (n = 11) [66,68,69,70,71,72,73,74,75,76,77];
(4) Maxillofacial prostheses (File S1; Table S8) (n = 14) [78,79,80,81,82,83,84,85,86,87,88,89,90,91];
(5) Periodontics and oral pathology (File S1; Table S9) (n = 5) [92,93,94,95,96];
(6) Orthodontics (File S1; Table S10) (n = 4) [97,98,99,100].

The inter-rater reliability was *K* = 0.72 (substantial agreement) [101].

The application of GRADEpro GDT showed that 8% (n = 7/85) of the papers were judged to be of high quality, 42% (n = 36/85) of moderate quality, 39% (n = 33/85) of low quality, and 11% (n = 9/85) of very low quality. In order to achieve oversight, it was considered apposite to include all 85 papers in the review. Of those judged to be moderate to very low quality, one-third (n = 27/85) reported convenience sampling, 17% (n = 15/85) failed to provide detailed descriptions of the camera apparatus, 13% (n = 11/85) did not describe how they controlled for variations in ambient light, and the remaining 30% adopted a more specific form of convenience sampling in which they failed to describe racial, professional, and anatomical variations. Racial variations are those present across different ethnic groups, professional variations occurred in papers that did not state the age and sex of the observers performing the visual analyses, and anatomical variations occurred when shades were recorded from different teeth (File S2; Table S14).

Of the 85 papers, 56 articles (n = 56/85) were eligible for critical appraisal using the JBI appraisal tools (File S2; Table S15). Twenty-nine articles (n = 29/85) were excluded because of inadequate comparative study evaluation presented via JBI [102]. Forty (72%, n = 40/56) articles met the JBI appraisal threshold of >8. Sixteen (29%, n = 16/56) articles fell below the threshold due to (i) sub-optimal study design characterisation; (ii) failure to consider confounding factors such as ambient lighting variations, camera calibrations, lens selection, and illumination sources and observer opinions; or (iii) failure to adequately describe the gold standard that they adopted.

Notably, 39% (n = 13) of investigators used commercial spectrophotometers or colourimeters not originally intended for dental applications. For brand names of spectrophotometers and colourimeters identified by this review, see File S1; Table S11.

### 3.2. Do Digital Systems Have Better Clinical Outcomes Than a Spectrophotometer, Colourimeter, or Conventional Visual Methods?

Of those 85 articles, 33 (see Table 1) satisfied the PICO. The 52 articles that were excluded and the reasons for their exclusion are stated in File S1; Table S12. Out of the articles examined, approximately 54% (18 out of 33) involved comparing measurements obtained from digital systems to those derived from a spectrophotometer or colourimeter [5,7,11,15,16,21,31,36,47,52,54,56,59,63,72,87,90,94]. Additionally, about 45% (15 out of 33) of the papers conducted comparisons with traditional visual analyses [3,4,5,15,21,38,39,43,45,47,61,75,78,97,103]. Of note, only five papers compared outcomes from digital photography, spectrophotometers and/or colourimeters, and visual analysis methods. Their findings are stated below:

1. The study in [15] compared the colour agreements for 50 maxillary incisor teeth recorded in a dental clinic during the daytime using calibrated digital photographs, measurements from a spectrophotometer, and visual methods using commercially available shade tabs (VITAPAN^®^ classical shade guide). The average colour differences (∆E) against the spectrophotometer measurements were 1.69, and statistical Z-test (raw scores above the mean, where a score of 0 indicates that the raw score is identical to the mean score) scores were similar (Z = −3.2) for both the spectrophotometry and the digital photography, yet the kappa agreement between visual shade matching and spectrophotometry was very low (*K* = 0.2).
2. The investigation in [11] recruited three independent observers who compared photographs obtained using an intraoral camera, a visual method using a commercial shade guide (Vita 3D-Master), and a spectrophotometer. Measurements from intraoral cameras were significantly correlated (*p* < 0.01), with conventional visual analyses at 1650 Lux light intensity and colour temperature (6500K and 3800K), although measurements from spectrophotometers showed only weak correlations when compared with both intra-oral cameras and conventional visual methods.
3. In the study in [5], all three methods were used to record restoration shades. The findings suggest that lighter shades produced lower colour differences (∆E = 2.60) when measurements were taken using photographs and a spectrophotometer. In contrast, darker shades of restorations produced larger colour differences for all methods (∆E = 7.7 to 8.2).
4. The study in [47] compared the effectiveness of camera white balance in determining the accuracy of selecting the shade tab with measurements from a spectrophotometer and the visual method. In a blinded test (visual method), the ability of practitioners to colour match improved significantly (*p* < 0.05) when the camera’s white balance was calibrated. In addition, they also found high correlations (r > 0.96, (*p* < 0.001)) between the measurements from a spectrophotometer and the use of digital photography.
5. Finally, the study in [21] compared 3D scanning to visual analysis and the use of a spectrophotometer. The authors reported that spectrophotometers more accurately captured shades compared to 3D scanning and visual analysis.

Below are the findings from other papers that compared digital photography with any of the other methods but not all three:

1. Fourteen papers [5,11,15,21,31,36,43,47,49,52,54,63,65,75] reported significant correlations between measurements obtained by digital photography and a spectrophotometer.
2. There were significant correlations observed for L* (lightness: r = 0.85) and b* values (yellow/blue axis: r = 0.96), but a* values (red/green axis: r = 0.58) showed a weaker correlation when measurements were compared with those from photographs and a colourimeter [16].
3. There was no correlation when comparisons were made for visual analysis (using the Social Appeal Scale (*p* < 0.01) and the geometric asymmetric index (*p* = 0.02)) and digital photography [97]. However, one article reported [38] that observers’ shade-matching ability improved significantly (*p* < 0.04) when digital photographs were shown on a computer screen.
4. The following eight studies reported ∆E value measurements from a spectrophotometer or colourimeter, digital camera, or visual methods, but not all three:
  i. ∆E = 0.09 (∆E = < 1.0 are colour differences that are not perceptible to the human eye) when predicted colour values were generated from a photograph using a regression model compared with colour values extracted directly from a photographed shade guide (Vident Inc., Brea, CA, USA) and software (Adobe Photoshop 7.0, San Jose, CA, USA) [49];
  ii. ∆E = 1.69 when comparisons were made for measurements from a spectrophotometer and digital photographs of maxillary incisor teeth [15];
  iii. ∆E = 2.3 (∆E = 2–10 are colour differences perceptible at a glance) when one set of digital CIELAB values was extracted directly from a computer screen and compared with the colour values measured with a colourimeter [75];
  iv. ∆E = 3.2 when CIELAB values were extracted using in-house software (Toodent, Babes-Bolyai University, Cluj-Napoca, Romania) from photographs of a shade guide (VITA Toothguide 3D-MASTER^®^, Bad Säckingen, Germany) and compared with spectrophotometer-generated values [31];
  v. ∆E = 6.94 when colour measurements were recorded from a 3D-printed custom shade guide, compared with measurements from a spectrophotometer [48];
  vi. ∆E = 7.35 when comparisons were made between visual analysis of maxillary central incisors and measurements from a spectrophotometer [7];
  vii. ∆E = 8.20 when comparisons were made between visual analysis and digital photographs of a reference tooth restored with resin composite shade A3 and a test tooth restored with composite shades DA4 and DA3.5 [5];
  viii. ∆E = 14.60 ± 5.20 for CIELAB values of carious tooth surfaces when photographed digitally compared with measurements from a colourimeter [94].

The present systematic review also identified the following findings of interest, although not directly related to our research questions:

1. Conventional visual methods for colour matching were also influenced by ill-defined observer opinions [3,4,43,44,84,89,103,104], training [4], gender [3,4], eye fatigue [43], and, strangely, observer monthly income [4]. With respect to monthly income, it was reported that observers with higher incomes preferred lighter tooth shades, whereas those from lower income groups preferred darker shades.
2. Colour selection for ceramic restorations using photographs was shown to produce significantly different a* values (red to green) compared with colours matched using conventional shade tabs, resulting in the restoration appearing darker, with a greenish-yellow tint. Furnace-firing temperature of the ceramic, underlying coping materials, thickness of the restoration, and ceramic product brands also resulted in shade mismatches [70].
3. Machine learning algorithms such as support-vector machines were used to enhance digital colour spaces [10,46] because they can mitigate inconsistency in ambient lighting when used in conjunction with digital or intra-oral cameras. Although there were minor shifts in a* (reddish tint) and b* (yellowish tint) values [46], there was good inter-device reliability.

The included studies were heterogenous with respect to observers and quantification of the conventional visual method, both contact and non-proximity devices, ambient lighting conditions, and sample sizes. It was therefore not possible to carry out a meta-analysis.

### 3.3. Results from the Updated Search

Nineteen additional articles [104,105,106,107,108,109,110,111,112,113,114,115,116,117,118,119,120,121] were identified after the initial search, and their results are reported in Table S4 and included in the Discussion section. The findings from these additional articles mirrored those from the initial search and therefore did not warrant a revision to the original study methodology [122].

## 4. Discussion

This review aimed to first identify the non-proximity digital recording instruments and colour spaces frequently applied in dentistry for shade matching. While doing so, it was seen that digital shade matching had numerous study design variations and could not be challenged by a singular gold standard. For the second objective, a comparison was made across non-proximity methods, contact-measuring methods (spectrophotometer and colourimeter), and visual shade matching. It was seen that, under controlled environments, non-proximity digital systems exhibited similar clinical results as their contact device competitors, yet conventional shade matching still possessed an overarching influence on dictating shades in dentistry.

Conventional visual colour matching using shade guides remains the most commonly used method in dentistry [45]. The alternatives, such as a spectrophotometry or colourimetry, can be prohibitively expensive; for example, a VITA Easyshade V spectrophotometer costs approximately GBP 2000. In addition, the potential purchaser may be influenced by the literature reporting that spectrophotometers lack some accuracy, as the measurements from these instruments are influenced by tooth curvature and enamel translucency [40,65] and have poor inter-device reliability [13].

The accuracy and predictability [10,48] of the conventional visual method has been questioned. What can be learned from the blue jay? When viewed using a backlight, the feathers are brown because they contain the pigment melanin. But when ambient light hits the small pockets of air and keratin in the feathers, all of the colours of the wavelength except blue are absorbed [123]. When applied to shade matching, a tooth demonstrates visual anisotropy because a tooth comprises enamel, dentine, and pulp. In contrast, a resin composite restoration exhibits isotropy with uniformity in optical properties.

Shade matching is often linked with metameric failure. llluminant metameric failure (illuminant metamerism) is when the shades from two objects match when viewed under one light source but not another. Therefore, daylight photography (ca. 6000K at noon) of teeth produces significantly different images compared with dental photographs of teeth when taken indoors (2700K). It affects, in particular, the red and bluish tints on dental shade tabs [30,47,52]. Interestingly, the use of digital photography to compare commercially available dental shade tabs shows varying degrees of colour mismatch, with custom-made shade tabs resulting in only marginal improvements [11,33,35,60,61].

Geometric metamerism is the effect of colours matching when viewed from one angle but not from a different angle. Colour blindness is an example of observer metamerism and can result in major colour mismatches when tooth shade matching is carried out using visual methods [5,7,15,31,48,49,75,87,94]. Field size metamerism occurs when colours match when viewed at the centre but appear different when large field areas are viewed. Finally, device or instrumental metamerism occurs when different readings are obtained for the same colour from the same instrument because of variations within the instrument’s spectral response. This could be because some of the shade-taking devices used by dentists are not designed for dental use [7,12,15,21,76]. Spectrophotometers and colourimeters are used in dentistry with the aim of minimising such metameric failures.

### 4.1. An Absence of a Gold Standard When Matching Shades

In answering our first research question, the methodology was straightforward. However, it was more challenging to answer the second research question specifically comparing the clinical outcomes of digital systems with spectrophotometers or colourimeters and conventional visual methods, as there was an assumption that measurements from spectrophotometers are the gold standard, and therefore, many studies restricted their comparison to only this device.

Almost two decades ago, P Finbarr Duggan [124] published a letter in the British Medical Journal that stated, “Because the phrase (‘Gold Standard’) smacks of dogma its use should be discontinued in medical science”. When deciding on our methodology, we were persuaded by the pyrite principal [18], as we soon realised that there is no single gold standard for dental colour analysis. This is supported by the adoption of the term ground truth, which has been used in machine learning, including its application in tooth shade taking [10,46,50,51]. In our study, we adopted the concept of criterion standards in that the term encompasses the best fit for those conditions [19]. For example, one criterion standard for shade matching would be the conventional visual method that has been used from time immemorial, but others have argued that a more contemporary criterion standard would be the use of a spectrophotometer or colourimeter.

### 4.2. Measurements Used in Visual Shade Matching

Although shades recorded using spectrophotometers, colourimeters, and cameras use straightforward recording instruments that provide defined values, there is no agreed method of shade matching using the visual method. Attempts have been made to quantify visual methods, such as asking study participants to select shades from shade guides [21,45,48,49,65]; using the (i) Acceptability Test, where the observers are asked whether the change in colour matches their visual judgements [3,4,5,78]; and the (ii) Agreement Test, where the participants are asked whether they judge that shades from photographs are the same as shades chosen in real life [15,61,75,103].

### 4.3. Why Is There an Increasing Use of Non-Proximity Digital Recording Instruments for Shade Matching?

Smartphones have become integral to our life [9,40,125], with ownership standing at approximately 60 to 80% of the global population (https://www.statista.com/statistics/203734/global-smartphone-penetration-per-capita-since-2005/, accessed on 24 October 2023). They are relatively inexpensive and easy to use; facilitate dentist-to-patient communication, including colour manipulation; and are non-proximity, resulting in allayed infection control concerns [3,5,32,38]. Most studies [12,13,46,79,109,110,111,112,114,115] that use smartphone cameras for dental shade matching use Apple Inc. products. Such luxury brands have a limited market share in emerging and developing countries, where less expensive Android-based smartphone manufacturers are dominant. Of note, costly flagship smartphones may not be a prerequisite for capturing well-calibrated photographs for applications in dentistry.

Digital cameras have an ever-increasing role in dental cosmesis in that the patient can take part in designing their smiles and tooth shapes and choosing shades [5,8,48,79,111,112,113]. Interestingly, dentists’ preference for using photography when shade matching [15,74,75,109] may merely be a function of what economists refer to as utility in that there is considerable value in using a camera or smartphone, even if they are expensive, as long as they minimise restoration re-makes.

The applicability of 3D scanners in tooth shade matching is equivocal, with some scanners not even meeting their primary function of producing accurate casts [8]. However, 3D scanners may have a role in recording surface topology in that they can be used to record the relationship between objective measurements and subjective perception, the geometric colour asymmetry index. It has been argued that changing the viewing angles of the observer and the angles of illumination of the 3D scanner greatly reduces device-dependent inter-observer reliability [110,115] and colour shifts, which are changes in the colour intensity of an object surface as a consequence of the viewing angle or the angle of the illumination source [8,21,97]. Some investigators [16,48] claim that 3D scanners can measure lightness and ∆E differences in tooth shades with greater accuracy and consistency compared with the use of spectrophotometers or colourimeters.

### 4.4. Factors That Influence the Accuracy of Shades Taken Using Digital Photography

Non-proximity instruments are not without their shortcomings. Colour accuracy is dependent on several factors, such as camera calibrations, lens selection, illumination sources, colour temperatures, and ambient light [13,15,30,36,37,40,45,47,49,52,80,108,126]. Most investigators [5,7,8,9,13,31,35,36,42,43,44,57,65] preferred using a macro lens with focal lengths (distance between the camera lens and the image sensor) between 50 and 105 mm; shorter focal length lens resulted in wider viewing angles and lower magnification of the target area (File S1; Table S13). In order to obtain ∆E values close to the acceptable threshold, the aperture (amount of light accessing the image sensor) should be kept at f/10 when using a light filter and f/29 for normal photography under direct light without filters [9]. This is because filters allow only a specified amount of light to access the lens, although the use of higher apertures limits that amount even further, thereby causing colour distortions near the periphery. Unwanted reflections and glares blight photographs of reflective surfaces, but can be mitigated by the application of tungsten lights with colour filters [85,88,91] and computerised white balance calibration [116,117]. White balance is the process that removes unwanted colour casts (a tint of a particular colour, usually unwanted, that affects the photographic image). This can be achieved either automatically or manually by implementing computerised methods. Almost half of the investigators (68.42% of authors) preferred manual white balance calibration of their photographic devices because manual white balance calibration reduces undesired environmental lights and significantly improves digital colour accuracy compared with automatic camera white balance correction [47,57] (File S1; Table S13).

### 4.5. Other Factors Affecting Shade Matching

As salivary reflections adversely affected the L* (lightness) and a* (red/green) values, investigators suggested that shade matching should be carried out on teeth that have been dried or, alternatively, using polarising filters [72,116]. However, drying a tooth may be counterintuitive in that teeth are always bathed in saliva.

Colour temperature is a numerical system measured in Kelvin (K) that measures the colour based on its warm (reddish) to cool (bluish) spectrum. Only some investigators [10,11,16,43,45,46,52,59,63,77,84,91] used an ambient colour temperature within the range of 5332K to 6500K (outdoor shade), implying that the images were taken with an electronic flash. An overcast sky has a colour temperature of 6500–8000K (more bluish), whereas the colour temperature in a closed room/photography studio varies between 2500K and 3500K (more reddish). File S1; Table S13 provides the calibrations used in the included studies. The practical application of this is that the patient has to decide, guided by the dentist and the technician, whether the restoration has an ideal shade match when viewed indoors or outside.

### 4.6. The Future of Digital Shade Matching in Dentistry

In the future, there may be a role for machine learning in tooth shade matching. Machine learning is a form of artificial intelligence that uses historical data to predict the new output. In conjunction with the use of digital or intra-oral cameras, machine learning has been used to enhance digital colour spaces [10,46] to mitigate inconsistency in ambient lighting [10,50,51,114]. Such technology is already being implemented with third-party applications such as Shadent software (patent no. 201841046815, Intellectual Property of India) and Chromatcher (DMP Dental Industry, S.A.). It has been suggested [107,119,120] that artificial intelligence-driven digital photography will become the new *Criterion Standard* for dental shade matching. Notably, the study also highlights a scarcity of high-quality research in this particular field of dentistry, underscoring the urgent need for more high-quality studies to be conducted.

When a search update was performed in October 2023, the newly added articles [104,105,106,107,108,109,110,111,112,113,114,115,116,117,118,119,120,121] supported our earlier findings. It has been stated [122] that if the update shows no substantial change, the method and findings from the substantive search can be defended.

### 4.7. Limitations

1. To ensure a focused approach and manage the extensive data sources available, this article search was confined to specific databases and tailored research questions.
2. Recognising the complexity of summarising comparisons between non-proximity digital shade-matching instruments, spectrophotometers (considered a high standard), and visual shade assessment (considered a low standard), this systematic review acknowledges the need for additional approaches. To address various aspects within this research field, further investigations are required, such as considering the number and type of specimens (e.g., natural tooth, shade tab, restorative material), the study design (in vitro or in vivo), colour measurement conditions, experiment type, and reported accuracy and precision.
3. This systematic review did not include any brand-specific non-proximity or contact colour-measuring instruments, which allowed for a more generalised assessment of the field, considering a wider range of devices and their overall performance rather than specific brand characteristics.

## 5. Conclusions

The following conclusions may be drawn from the findings of the current systematic review:

- In recent decades, the utilisation of digital cameras and smartphones for recording tooth shades has witnessed a significant increase. Among the studies examined (n = 85), 42% (n = 35) opted to use the CIELAB (International Commission on Illumination) colour space for their analyses.
- Under controlled conditions, non-proximity digital instruments consistently demonstrated more reliable clinical outcomes compared to conventional visual tooth shade-matching methods.
- Under controlled environments, digital instruments were found to be equally effective when compared to the use of spectrophotometers and colourimeters.
- There is no universally accepted gold standard for tooth shade matching due to the multitude of variables involved.

## Figures and Tables

**Table 1:** Summary of papers identified.

Author (Year)	Digital Method Examined	Method Evaluated against	Analysis Performed	Study Outcomes	Associations and Correlations	Funding Sources
Mahn, 2020 [7]	Photographs of maxillary central incisor with cross-polarising filters (60 observers)	Spectrophotometer	∆E	∆E = 6.12	Not reported	Not stated
He, 2020 [36]	Photographs with and without cross-polarisation filter of maxillary incisor teeth	Spectrophotometer	CIELAB	Not reported	Highly significant correlations (*p* < 0.0001) were observed.	Not stated
Liu, 2019 [48]	3D-printed custom colour chart	Manufacturer-provided colour chart	∆E	∆E = 2.19–11.23 for teeth and gingival shades	Not reported	Capital’s Funds for Health Improvement and Research (CFH 2018-2-4101) The Natural Science Foundation of China (81801015) The New Medical Technology Program of Peking University Hospital of Stomatology (PKUSSNCT-19G01)
Lagouvardos, 2018 [78]	Digital photographs of facial skin colour (81 skin specimens)	Visual analysis	CIELAB	Δa* and Δb* values (2.0–2.5 units) that were judged acceptable	Not reported	Not stated
Yoon, 2018 [16]	Digitally scanned images	Colourimeter	CIELAB	Significant differences (*p* < 0.001) were observed for all three (L* a* b*) values.	L* (*p* < 0.05) and b* (*p* < 0.05) values were strongly correlated to each other but a* values (*p* < 0.05) showed a weaker correlation.	Not stated
Labban, 2017 [4]	Digital photographs	Visual Analysis	Acceptability Test	Shade preferences varied significantly (*p* ≤ 0.05) between male and female observers. Female observers preferred lighter tooth shades (*p* < 0.05).	Associations were found between observers’ preferred shade tabs and their education level (*p* = 0.036) and monthly income (*p* = 0.009).	King Saud University, Saudi Arabia
Mehl, 2017 [21]	Tooth shades obtained by the 3D scanner	Tooth shades selected by dental professionals Spectrophotometer	∆E	Spectrophotometer was more accurate than 3D scanner and visual method	Not reported	Not stated
Miyajiwala, 2017 [15]	Digital photographs of tooth shades	Spectrophotometer Visual analysis	∆E	∆E = 1.69 for the spectrophotometer	Significant agreement on tooth shade selection (kappa = 0.20; *p* < 0.01) for spectrophotometer and visual methods	Not stated
Kim, 2017 [103]	Digital photographs of dental restorations one month following placement (2 observers)	Visual analysis	Agreement	Significant differences (*p* < 0.001) between methods were found.	Observer 1 showed higher proportions of agreement (Pa = 0.58–0.97) than Observer 2 (Pa = 0.53–0.73).	The Ministry of Health and Welfare, Republic of Korea (HI16C-0272-010016)
Rauber, 2017 [5]	Digital photographs of restorations with different shades (GA3.5, GA4) placed at different cavity depths (0.5 mm, 0.7 mm, 1 mm)	Visual analysis Spectrophotometer	∆E	GA3.5: ∆E = 2.60 GA4 at 0.7 mm: ∆E = 7.7 GA4 at 0.5 mm: ∆E = 8.2	Laypeople identified colours a little more accurately (87%) than dentists (83%). Lighter shades of restoration were more acceptable for cavity depths of 0.5 mm and 0.7 mm. Darker shades of restoration were more acceptable for 1.0 mm-deep cavities.	Funding was not stated (but materials were donated by Ivoclar Vivadent Inc.).
Lakhanpal, 2016 [72]	Digital photographs of extracted premolar teeth	Spectrophotometer	CIELAB	L* and a* values produced significantly different outcomes (*p* < 0.001), whereas no difference was observed for b* values (*p* > 0.05).	Not reported	Not stated
Berssenbrügge, 2015 [97]	Digital photography	Visual analysis (asymmetry index)	Correlation	Not reported	Colour asymmetry index and geometric asymmetric index showed significant correlation (r = 0.43, (*p* = 0.017))	Deutsche Krebshilfe (German Cancer Aid)
Culic, 2014 [31]	In-house software (TooDent)	Spectrophotometer	∆E	In-house software-generated photograph in ∆E < 3.20 for 81% of readings	Strong correlation (r = 0.91; *p* < 0.001) observed between in-house software -generated photographs and spectrophotometry	UMF internal Grant (27020/18/2011)
Montero, 2014 [3]	Digital photographs of teeth with different shades	Visual analysis (Dental students)	∆E	∆E values for darker tooth colours were 8.5 for females and 6.4 for males. ∆E values for lighter tooth colours were 9.9 for females and 8.6 for males	Social Appeal Scale was correlated with psychological competences (r = 0.87), relationship satisfaction (r = 0.84), and social abilities (r = 0.83; (*p* < 0.01))	Department of Surgery of the University of Salamanca
Xiao, 2014 [87]	Camera photogrammetry to create 3D skin colour chart	Spectrophotometer	∆E	ΔE = 3–4	Not reported	Wellcome trust Translational Research Award UK—Automated Rapid Manufacture of Facial Soft Tissue Protheses and Fripp Design Limited, UK
Xiao, 2013 [90]	3D-printed colour chart for skin colour	Spectrophotometer	∆E	∆E < 3.0	Not reported	Wellcome trust Translational Research Award UK—Automated Rapid Manufacture of Facial Soft Tissue Protheses and Fripp Design Limited, UK
Tam, 2012 [45]	Digital photographs of shade guide	Visual analysis	Accuracy of RGB, HSV, XYZ, and CIELAB	Increased accuracy with both CIELAB and HSV (0.75 and 0.67) compared with RGB (0.55) and XYZ (0.50)	Not reported	Not stated
Lasserr, 2011 [11]	Photography using an intraoral camera	Direct visual analysis	Chroma (L, M, R)	Not reported	Significant agreement (*p* < 0.05) for intraoral photography compared with direct visual analysis for shades of canines and central incisor teeth	Not stated
	Direct visual analysis	Spectrophotometer		Not reported	Correlation co-efficient was lower when comparing direct visual analysis with spectrophotometer	
	Indirect and direct visual analysis	Spectrophotometer		Not reported	Correlation coefficient was higher (*p* < 0.01) when comparing direct visual analyses and intraoral photographs	
Athanasios, 2011 [56]	18% grey card-calibrated digital photographs	Spectrophotometer	CIELAB	Spectrophotometer errors were greater (L* = 1.44, a* = 0.43 and b* = 0.62) than those of calibrated digital photographs: (L* = 0.97, a* = 0.67, and b* = 1.25).	Not reported	Not stated
Delalleau, 2011 [88]	Digital photography and software calibration to measure skin colour	Colourimeter	∆E	∆E < 3.0	Not reported	Not stated
Tung, 2010 [47]	Shade tabs generated in CWB (Camera White Balance) and AWB (Auto White Balance) setups	Spectrophotometer and visual analysis (observers were asked to compare photographs generated using CWB and AWB)	Correlation	Colour -matching abilities of operators improved significantly (*p* < *0*.05) from 67% in AWB to 93% when using CWB	Significantly high correlation was found, (r > 0.96, (*p* < 0.001)) between CWB and spectrophotometer. No significant correlation was found (r = 0.04, (*p* = 0.483)) for a* values when all three methods were compared.	Taipei Veterans General Hospital, Taipei, Taiwan (V96C1-045)
Lindsey, 2010 [39]	Digital photographs of maxillary incisors	Visual analysis	∆E	∆E = 1.45–2.90	Not reported	National Eye Institute (R15 EY013527) National Institute of Dental and Craniofacial Research (K23 DE016890)
Yamanel, 2010 [59]	Digital photographs of dental composite resin	Colourimeter	∆E	Significant differences were observed for L* (*p* < 0.05) and a* (*p* < 0.05) values, whereas b* (*p* > 0.05) showed no significant difference	Not reported	Not stated
Won-suk Oh, 2010 [75]	Multiple photographs of shade tabs were quantified using the photo colourimetric method (PCM).	Visual analysis: Observers were asked to choose the best matched shade tabs from a computer screen.	∆E	∆E = 2.3	Not reported	Not stated
Caglar, 2009 [52]	Digital photographs of dental shade tabs	Colourimeter	∆E	Colourimeter showed lower L* values (*p* < 0.01) than standard digital photographs	Not reported	Not stated
	Digital photographs taken at different colour temperatures	Colourimeter	∆E	At 2700 K, a* and b* values for digital images exhibited no significant colour differences (*p* > 0.01). At 2700K to 6500K and beyond, there were significant colour differences in a* and b* (*p* < 0.01 and *p* < 0.001, respectively).	L* and b* values obtained from colourimeter and digital photographs showed high levels of correlation.	
Schrop, 2009 [43]	Software-calibrated photographs	Visual analysis performed in clinic	HSV	When viewing on a computer screen, observers showed significantly better (*p* < 0.02) tooth colour-matching capabilities for software-calibrated images compared with visual analysis.	Not reported	Not stated
	Digital photographs of teeth	Visual analysis	HSV	No significant difference (*p* > 0.02) was found between digital photographs and visual analysis.	There was a significant correlation (*p* = 0.01) between the times taken to perform the computer screen-based colour analysis procedure and the visual analysis method.	
Jarad, 2008 [65]	Digitally measured colour values of shade tabs	Manufacturer-provided colour values of shade tabs	∆E	Not reported	Significant correlation (r = 0.97, (*p* < 0.01)) was observed between the digital and manufacturer-provided colour values.	Not stated
Iwami, 2007 [94]	Digital photographs of caries following application of a detector dye	Colourimeter	∆E	∆E ranged from 4.70 ± 2.90 to 14.60 ± 5.20	Not reported	Origination of Frontier BioDentistry at Osaka University Graduate School of Dentistry Grant-in-Aid for Scientific Research (A) (14207081) and (C) (17591990 and 19592199)
Gadhia, 2006 [61]	Observers used photographs with a custom-made shade guide	Visual analysis (using the Lobene Stain Index)	Agreement	Not reported	Photographs of custom-made shade guide showed better agreement among observers (*K* = 0.57 to 0.93) than use of the Lobene Stain Index (*K* = 0.38 to 0.79)	Not stated
Lath, 2006 [54]	Digital photographs of stained teeth before and after removal of stains	Spectrophotometer	Only L* value measured	Not reported	Strong negative correlation coefficient (r = 0.98)	Not stated
Wee, 2006 [49]	Photographs of dental shade tabs taken with various digital cameras calibrated using an in-house algorithm	Manufacturer-provided colour values	∆E	All cameras showed significant differences (*p* = < 0.0001).	High correlation was observed (r = 0.98) between digitally evaluated and manufacturer-provided colour values.	USPHS grant from the National Institutes of Health (R15 EY013527)
Jarad, 2005 [38]	Software-based colour calibration (Adobe Photoshop^©^)	Visual analysis	∆E	Significant (*p* < 0.001) differences between software-based colour calibration (Adobe Photoshop^©^) and visual analysis	Not reported	Not stated
Guan, 2005 [63]	Digital photographs of teeth during bleaching	Spectrophotometer	Effect of brushing time on tooth whitening using a toothbrush	Not reported	Intervention had little effect on either image (*p* < 0.31) or spectrophotometer (*p* < 0.22)	Not stated
			Whitening effect of 43% hydrogen peroxide bleaching agent	Not reported	Significant (*p* < 0.05) whitening effect for both regimens	

∆E = colour difference; proportion of agreement = Pa; Cohen’s kappa = *K*; SAS = Social Appeal Scale; RGB = red, green, blue; HSV = hue, saturation, value; CIELAB = *L refers to lightness and a* and b* refer to colour characteristics, where a* is the red-to-green axis and b* is the yellow-to-blue axis; conventional colour analysis = visual me.

## Data Availability

All supporting data has been made available as Supplementary Files (Files S1 and S2) provided with the manuscript.

## Supplementary Materials

The following supporting information can be downloaded at: https://www.mdpi.com/article/10.3390/dj11110250/s1, File S1; Table S1: Definitions with mathematical formulae. File S1; Table S2: Search keywords with database results (as of November 2020). File S1; Table S3: Articles that were excluded following investigation of the paper. File S1: Table S4: Summary of data synthesised from a search update (up to October 2023). File S1; Table S5: Summary of findings for colourimetry in dentistry. File S1; Table S6: Summary of findings for endodontics and dental aesthetics. File S1; Table S7: Summary of findings for prosthodontics. File S1; Table S8: Summary of findings for maxillofacial prostheses. File S1; Table S9: Summary of findings for periodontics and oral pathology. File S1; Table S10: Summary of findings for orthodontics. File S1; Table S11: Details of the colour analysis devices that were used as criterion standards. File S1; Table S12: Newcastle–Ottawa scoring: reasons for further exclusion of papers. File S1; Table S13: Camera-oriented preparation used for shade taking. File S2; Table S14: Quality of study analysis using Cochrane GRADEpro GDT. File S2; Table S15: Joanna Briggs Institute (JBI) critical appraisal results.
