# Supplementary material for: Digital Shade Matching in Dentistry: A Systematic Review
Source: Dent J (Basel). 2023 Oct 27;11(11):250. doi: 10.3390/dj11110250 (PMC10670912; doi:10.3390/dj11110250)
Supplement: Supplementary file 1 [file dentistry-11-00250-s001.zip › File S1 Table S1 to S13.pdf]

DIGITAL SHADE MATCHING IN DENTISTRY:

A SYSTEMATIC REVIEW

Supplemental Tables S1 to S13

Contents

Table S1: Definitions with mathematical formulae 2
Table S2: Search keywords with database results (*as of November 2020*) 5
Table S3: Articles that were excluded following interrogation of the paper 7
Table S4: Summary of data synthesized from a search update *(up to October 2023)* 10
Table S5: Summary of findings for Colourimetry in Dentistry 16
Table S6: Summary of findings for Endodontics and Dental Aesthetics 34
Table S7: Summary of findings for Prosthodontics 44
Table S8: Summary of findings for Maxillofacial Prostheses 50
Table S9: Summary of findings for Periodontics and Oral Pathology 55
Table S10: Summary of findings for Orthodontics 58
Table S11: Details of the colour analysis devices that were used as criterion standards 61
Table S12: Newcastle-Ottawa scoring: reasons for further exclusion of papers 63
Table S13: Camera oriented preparation used for shade taking 67

Table S1: Definitions with mathematical formulae

**RGB (red, green, blue)**	A colour space that minimises light intensity and sensitivity by projecting the RGB colours through the RGB chromaticity triangle.
**HSV (hue, saturation, value)**	An alternative representation of the RGB colour model but adopts human perception to describe the colour values.
**HIS (hue, saturation, intensity)**<br><br>**HLS (hue, lightness, saturation),**	These are the variants of HSV colour space.
**YUV (luma, blue projection, red projection)**	A colour encoding system in which human perception is used to encode the colours from both images and video recordings.
**YCbCr (luminance, Chrominance-blue, Chrominance-red)**	A RGB dependent colour space. This colour space is commonly used for digital video standards and television transmissions.
**YIQ (luminance, in-phase, quadrature),**	A system used by the National Television System Committee (NTSE) colour TV system which uses human colour response characteristics.
**XYZ** t**ristimulus values**	One of the first standard reference values, from which other more recent colour spaces have been developed.
**CIE**LAB	It is the colour model mapping system developed by International Commission on Illumination that uses a combination of three tristimulus values (red/green/blue) and plotted in a three-dimensional (3D) space. It is claimed it can quantify every colour the human eye can distinguish.
**CIE76**	The first algorithm [t] developed algorithm by CIE and claimed to produce more uniform colour values.
**CIE94**	This revised algorithm proposed by CIE to resolve the non-uniformities within the CIE76 algorithm.
**CIE2000**	This is the most precise algorithm proposed by CIE, although the most complex.

**L*a*b***	*L refer to lightness, a* and b* refer to colour characteristics in which a* is red to green axis and b* is yellow to blue axis. This is a three-dimensional colour space closely aligned with the visual colour space.
**ΔE (Euclidian colour differences)**	Proposed by CIE to calculate the colour difference between two subjects.
**U*V*W* (U* V* refers to chrominance and W* refers to luminance)**	A uniform chromaticity scale proposed by CIE that can offer more perceptual colour values than CIELAB.
**L*u*v* (*L refer to lightness and U* V* refers to chrominance)**	Developed by CIE to replace U*V*W* colour space the L*u*v* colour space is used mostly in computer graphics.
**$I_1I_2I_3$**	A statistically independent component colour space that decorrelates the RGB components and stabilise RGB images.
**SVM (Support-Vector Machine)**	The supervised machine learning model analyses data using regression analysis resulting in image segmentation and feature extraction.
**Formula to calculate ΔE from CIE76**	$\Delta E*_{76} = \sqrt{}(\Delta L)^2 + (\Delta a)^2 + (\Delta b)^2$
**Formula to calculate ΔE from CIE94**	$\Delta E*_{94} = \sqrt{(\frac{\Delta L*}{k_L S_L})^2 + (\frac{\Delta C*_{ab}}{k_C S_C})^2 + (\frac{\Delta H*_{ab}}{k_H S_H})^2}$
**Formula to calculate ΔE from CIE2000**	$\Delta E*_{00} = \sqrt{(\frac{\Delta L'}{k_L S_L})^2 + (\frac{\Delta C'}{k_C S_C})^2 + (\frac{\Delta H'}{k_H S_H})^2 + R_T \frac{\Delta C'}{k_C S_C} \frac{\Delta H'}{k_H S_H}}$<br>*ΔL'= lightness difference; ΔC' = chroma difference; ΔH'= hue value difference*
**Formula for conversion of RGB to CIELAB**	1. RGB to XYZ [same white point (i.e., define individual colour within the colour spaces) D65] $X = C_{xr}R$<br>2. RGB to XYZ [new white point D50, Bradford correction (reference white point)] $X = BC_{xr}R$

	3. XYZ to LAB (reference white X_n)<br>$$X_1 = \frac{X}{X_n};\ Y_1 = \frac{Y}{Y_n};\ Z_1 = \frac{Z}{Z_n}$$
**Formula for conversion of CIELAB to RGB**	1. LAB to XYZ<br>$$X = X_n X_1;\ Y = Y_n Y_1;\ Z = Z_n Z_1$$<br>2. RGB to XYZ [same white point D50]<br>$$R = C_{xr} X = C_{xr}^{-1} X$$<br>3. RGB to XYZ [new white point D65, Bradford correction]<br>$$R = (BC_{xr}R)^{-1} X = C_{rx} B^{-1} X$$<br>*Here,* C_{xr}*= Gamma correction,* BC_{xr}*= Bradford gamma correction*
**Formula for conversion of RGB to HSV** [1]	$$H = \begin{cases} undefined, if\ S = 0 \\ \frac{60 \times\ G - B}{\delta}, if\ m = R \\ \frac{60 \times B - R}{\delta + 120}, if\ m = G \\ \frac{60\ \times R - G}{\delta} + 240, m = B \end{cases}$$<br>$$S = \begin{cases} \frac{(m - n)}{m}, if\ m \neq 0 \\ 0 \end{cases}$$<br>$$V = m$$

Table S2: Search keywords with database results (*as of November 2020*)

Search keywords	Scopus®	PubMed.*gov*	Web of Science™
Maxillofac* AND photograph* AND (color OR colour)	38	59	32
Maxillofac* AND photograph* AND shade	4	5	5
Maxillofac* AND digit* AND (color OR colour) AND tech*	21	38	35
Maxillofac* AND computer* AND (color OR colour) AND tech*	55	67	58
Head AND computer*AND (color OR colour) AND prosthe*	21	35	27
Head-neck AND digit* AND (color OR colour) AND tech*	0	20	2
Compar* AND digit* AND (color OR colour) AND maxillofac*	19	37	21
Instrument* AND digit* AND maxillofac* AND (color OR colour)	3	11	14
Skin* AND (color OR colour) AND digit* AND match AND fac*	0	0	0
Skin* AND photograph* AND (color OR colour) AND match	35	45	144
Skin* AND photograph* AND (color OR colour) AND maxillofac*	6	17	6
Computer* AND spectrophoto* AND skin* AND shade	2	2	6
Computer* AND colourimeter* AND skin* AND shade	1	2	6
Smart* AND shade AND match	7	4	77
(Color OR colour) AND profile AND digit* AND dent*	19	23	39
Shade AND match AND compar* AND prosthe*	102	184	172
Shade AND match AND photo* AND dent*	24	72	95
Smart* AND shade AND com* AND dent*	9	2	8
Smart* AND match AND photo* AND dent*	0	9	12
Soft* AND match AND photo* AND dent*	25	105	136
Soft* AND (color OR colour) AND profile AND dent*	27	41	62
"L, a, b" AND digit* AND match AND skin*	1	1	2
CIELAB AND (color OR colour) AND skin AND digit*	17	9	28
Vectorscope AND match AND skin*	0	0	0
Microscop* AND shade AND photo* AND dent*	11	11	8

Prosthe* AND photograph* AND (colour OR colour) AND tech*	69	74	169
Prosthe* AND digit* AND (colour OR colour) AND shade	80	84	98
Topograph* AND maxillofac* AND digit*	8	15	10
CIELAB AND skin AND photo*	41	27	48
Dent* AND photograph* AND (colour OR colour)	106	148	138
Dent* AND photograph* AND shade	121	130	180
(Tooth OR teeth) AND shade AND digit*	173	139	253
(Tooth OR teeth) AND photo* AND (colour OR colour)	169	145	232
Colour AND match AND (tooth OR teeth) AND photo*	38	90	157
Smart* AND (tooth OR teeth) AND (color OR colour)	25	13	52
Colour AND comput* AND (tooth OR teeth) AND photo*	163	167	263
Smile AND digit* AND (color OR colour)	39	33	56
Orth* AND digit* AND shade	32	19	133
Colour AND photo* AND implant	16	21	58
Dent* AND implant* AND shade AND digit*	8	15	20
Comput* AND implant AND (color OR colour)	32	61	68
Comput* AND dent* AND abut* AND (color OR colour)	47	61	83
Digit* AND col* AND denture	48	37	82
Photo* AND col* AND bridge	6	3	30
Digit* AND shade AND denture	13	3	16
Smart AND photo* AND denture	2	1	2
Comput* AND photo* AND denture	13	12	30
Digit* AND col* AND fixed	12	9	83
**TOTAL**	**1738**	**2106**	**3256**

Table S3: Articles that were excluded following interrogation of the paper

Author name	Article title	Reasons for exclusion
**Souza, 2021** [2]	One-year clinical performance of lithium disilicate versus resin composite CAD/CAM onlays	No colour analysis was carried out
**Sánchez-García, 2020** [3]	Semantic and structural image segmentation for prosthetic vision	Did not address the research aims of our study
**Kabil, 2019** [4]	Effect of high light intensity bleaching protocol versus descending light intensities bleaching protocol on post bleaching teeth sensitivity: A randomized clinical trial	No colour measurements were made with digital cameras
**Askarian, 2019** [5]	Smartphone-based method for detecting periodontal disease	Conference proceedings only
**Espinoza, 2018** [6]	Using cross-polarized photography as a guide for selecting resin composite shade	No measurable outcomes/data
**Gavinho, 2018** [7]	Detection of white spot lesions by segmenting laser speckle images using computer vision methods	No colour analysis was carried out
**Vizcaya, 2018** [8]	Retrospective 2- to 7-year follow-up study of 20 double full-arch implant-supported monolithic zirconia fixed prostheses: measurements and recommendations for optimal design	No colour analyses
**Brokos, 2017** [9]	Digital photographic procedure for comprehensive two-dimensional tooth shade analysis	No measurable outcomes (data)
**Hein, 2017** [10]	eLABor_aid: a new approach to digital shade management	No measurable outcomes/data
**Mclaren, 2017** [11]	A technique using calibrated photography and photoshop for accurate shade analysis and communication	Review article
**Sharmili, 2017** [12]	Comparative analysis of image processing algorithms for visual prosthesis	Did not address the research aims of our study
**Pavlov, 2017** [13]	Electro-optical system for the automated selection of dental implants according to their colour matching	No measurable outcomes/data
**Coachman, 2017** [14]	Dynamic documentation of the smile and the 2d/3d digital smile design process	No colour analyses were carried out
**Chen, 2017** [15]	An image-processing strategy to extract important information suitable for a low-size stimulus pattern in a retinal prosthesis	Did not address the research aims of our study
**Brandao, 2016** [16]	Technique for preserving pupil size when photographing the iris for ocular prostheses	Opinion letter only

**Watson, 2014** [17]	Complete integration of technology for improved reproduction of auricular prostheses	Although spectrophotometers were used, digital cameras were not included in this study
**Elhabian, 2013** [18]	Clinical crowns shape reconstruction - an image-based approach	Conference proceedings only
**Walder, 2013** [19]	Photographic and video graphic assessment of the smile: Objective and subjective evaluations of posed and spontaneous smiles	The authors compared the diagnostic value of video graphics and photographic methods to reproduce posed smiles, but no colour measurements were taken
**Salat, 2011** [20]	Achieving a precise colour chart with common computer software for excellence in anterior composite restorations	No measurable outcomes/data
**Hatamleh, 2011** [21]	Porosity and colour of maxillofacial silicone elastomer	No colour measurements were made with digital cameras
**Kim-Pusateri, 2009** [22]	Reliability and accuracy of four dental shade-matching devices	No colour measurements were made with digital cameras
**Rosenstiel, 2009** [23]	Quantification of the aesthetics of dentists' before and after photographs	The digital photographs were used only to evaluate the height and width ratio of teeth
**Lovato, 2009** [24]	Evaluation of a computerised method for denture biofilm quantification: inter-examiner reproducibility	Describes only an imaging tool to quantify complete denture biofilms
**Griffin, 2008** [25]	Use of digital photography to improve composite resin selection and material placement	Lecture presentation only
**Chu, 2007** [26]	Clinical steps to predictable colour management in aesthetic restorative dentistry	Digital cameras were not introduced to the workflow
**Griffin, 2007** [27]	Assessing aesthetic composite veneer placement via digital photography	Lecture presentation only
**Stumpel, 2004** [28]	Simplifying the correction of the digital image in shade communication	No measurable outcomes/data
**Sykes, 2004** [29]	Applications of rapid prototyping technology in maxillofacial prosthetics	Book chapter only
**Bengel, 2003** [30]	Digital photography and the assessment of therapeutic results after bleaching procedures	No measurable outcomes/data
**Yong Ng, 2003** [31]	Non-contact imaging colourimeter for human tooth colour assessment using a digital camera	Conference proceedings only
**Dancy, 2003** [32]	Colour measurements as quality criteria for clinical shade matching of porcelain crowns	No colour measurements were made with digital cameras
**Phelan, 2002** [33]	Use of photographs for communicating with the laboratory in indirect posterior restorations	No measurable outcomes/data

**Dirksen, 2001** [34]	Three-dimensional quantification of colour-marked occlusal paths on anatomically oriented casts	Describes only a method to evaluate occlusal contacts
**Juan, 1999** [35]	Computer-aided periodontal disease diagnosis using computer vision	Describes only a method to digitally evaluate the pocket depth for diagnosing periodontal diseases
**Inaba, 1997** [36]	A computer-assisted videodensitometric method to visualize mineral distributions in in vitro and in vivo formed root caries lesions	Describes only a method for determining the mineral distribution in dentinal lesions
**Swineford, 1994** [37]	A digital transillumination technique for incipient lesions on teeth	Describes only a transillumination method, using a scanner to detect incipient lesions on the tooth surface.
**Gazi, 1988** [38]	Photographic assessment of the antiplaque properties of sanguinarine and chlorhexidine	No colour analysis was carried out

Table S4: Summary of data synthesized from a search update (up to October 2023)

Author Year	Title	Summary of Methods	Outcome
Chung, 2023[39]	Perceptual and quantitative analysis of discoloration of orthodontic elastomeric chains by food	**Samples:** 9 typodont models were prepared, and ceramic brackets were bonded to six maxillary anterior teeth.<br><br>**Colour analysis:** Orthodontic elastomeric chains were immersed in curry, coffee, and wine solutions over 28 days and photographed using digital camera with two fluorescent tubes In a dark room. CIELAB values were extracted from the photographs.<br><br>A web-based survey gathered visual assessments from 50 respondents where observers were between 20–40 years old.	Results showed significant differences in discoloration among immersion solutions, with curry causing the highest discoloration (ΔE= 6.34), followed by coffee and wine (ΔE= 4.46, and 9.98, respectively)<br><br>Visual scoring correlated significantly with discoloration, especially in the early stages.
Ginesin, 2022[40]	Digital photometric analysis of gingival response to periodontal treatment	**Samples:** 40 patients diagnosed with periodontitis (stage III/ IV, grade C)<br><br>**Colour analysis:** digital photographs were recorded using a DSLR camera before initial treatment (T0), 6-8 weeks after treatment (T1), and 3 months post-surgery (T2). CIELab values were extracted from the photograph and compared with proposed naked eye colour threshold (ΔE= 3.7)<br><br>A ring light and a custom white balance were applied when the gingiva was photographed.	Results showed a significant reduction in the a*-value (indicating a decrease in red colour intensity) from T0 (32.01) to T1 (29.28) with further reduction at T2 (27.45).
Rashid, 2022[41]	Color variations during digital imaging of facial prostheses subjected to unfiltered ambient light and image calibration techniques within dental clinics: An in vitro analysis	**Samples:** 432 measurements were collected from pigmented silicone samples<br><br>**Colour analysis:** Images of the silicon samples were captured without calibration (raw) and with two white balance calibration (WBC) methods: post-processing WBC (PPWBC) using a Macbeth colour chart and an 18% neutral gray card, and camera calibration using a gray card. A windowed and a non-windowed clinic were used to capture images<br>LAB colour values were extracted from the images, and colour differences (ΔE) were compared to that of spectrophotometric values.	The study found significant colour variations in maxillofacial prosthetic specimens due to natural ambient light variations. Camera white balance calibrations (CWBC) and PPWBC using colour charts were effective for colour correction in windowed clinics, while CWBC and PPWBC using gray cards produced better outcomes in windowless setups.
Jorquera, 2022 [42]	A comparison of ceramic crown colour difference between different shade selection methods: Visual, digital camera, and smartphone	**Samples:** 45 patients who required ceramic restoration<br><br>**Colour analysis:** Shades were selected once visually by using shade guides and then digitally from photographs; from both smartphone and professional digital camera	There were no significant ΔE difference (P=.857) was observed for both the digital photographs and smartphone images (ΔE= 2.75 and 2.34, respectively)<br><br>visual selection and digital photography produced significantly different colours (ΔE= 5.32)

		White balance was calibrated by using a software by using a grey reference card.<br><br>Then the crown was prepared by using the selected shade tabs and the ΔE values were evaluated between the crown and adjacent teeth	
Sirintawat, 2021[43]	The Accuracy and Reliability of Tooth Shade Selection Using Different Instrumental Techniques: An In Vitro Study	**Samples:** 30 central incisor crowns<br><br>**Colour analysis:** the crowns were photographed using digital single-lens reflex (DSLR) cameras with twin flash and polarized filter, DSLR cameras with a ring flash and polarized filter, smartphone cameras with light corrector and polarized filter, intraoral scanner, and spectrophotometer.<br><br>Reliability was assessed through intraclass correlation and accuracy was evaluated by calculating ΔE (colour difference) compared to a control group (spectrophotometer)	Results indicated satisfactory reliability (intraclass correlation) for all methods. However, the ΔE values showed statistically significant differences from the clinically acceptable range (ΔE = 6.8).<br><br>Digital camera with twin flash, smartphone camera and spectrophotometer showed similar accuracy (ΔE = 10.90, 10.57, and 11.57, respectively), while DSLR cameras with a ring flash showed least accuracy (ΔE = 19.98)
Abreu, 2021 [44]	Analysis of the colour matching of universal resin composites in anterior restorations	**Sample:** 6 class III cavities were restored by 2 different resin composites.<br><br>**Photographs:** Samples were photographed by using cross polarizing filter and white balance were corrected by using Grey reference card<br><br>**Colour analysis:** CIEDE-2000 formula was used to evaluate the colour differences ΔE<br><br>**Other analysis:** Visual scoring was done by observers to score the colour matching	In case of ΔE values, no significant differences (P>.05) were observed for both the photographic and visual scoring methods when used in different shades but in same composite groups.<br><br>Furthermore, a positive co-relation (r=.205) was observed between both the methods
Duong, 2021 [45]	Automated caries detection with smartphone colour photography using machine learning	**Sample:** 620 unrestored molar/premolar extracted tooth<br><br>**Photographs:** A smartphone camera was used to photograph the teeth. Photographs were labelled based on the ‘International Caries Detection and Assessment System’ (ICDAS) codes by 4 observers<br><br>**Caries analysis:** 2 sets of Support Vector Machine (SVM) algorithm were proposed	Both the algorithm exhibited improvement in accuracy, sensitivity, and specificity, thus suggesting smartphone camera images can be use in caries detection while in combination with machine learning<br><br>However, those algorithms still need further improvement and verifications

		Afterwards the algorithms were compared based on their accuracy, sensitivity and specificity and best algorithm was evaluated for caries detection	
Ebeid, 2021 [46]	Accuracy and repeatability of different intraoral scanners on shade determination	**Sample:** 10 different shades of ceramic blocks were fabricated<br><br>**Colour analysis:** Each sample was measured 10 times by using spectrophotometer and 3 different intraoral scanners.<br><br>Afterwards, accuracy and repeatability of the procedures were evaluated	There were highly significant differences in accuracy for all instruments (P<.001) with substantial inter-device variability and low repeatability (44.3% for spectrophotometer and 51.9% for intraoral scanners)
Hein, 2021 [47]	Objective shade matching, communication, and reproduction by combining dental photography and numeric shade quantification	**Sample:** Maxillary right central incisor<br><br>**Photographs:** The tooth was dried and images were taken by using a cross polarizing filter.<br><br>eLAB prime software was used to calibrate the white balance. trichromatic subtractive colour mixing technique was used to obtain the desired shade. Digital try in was performed by using eLAB software and necessary adjustments were made prior to patient supply	Authors concluded that eLAB software could predict and digitally portray accurate tooth colours when photographs were taken with polarizing filters
Ulloa, 2021 [48]	A Digital Method to Fabricate the Ocular Portion of An Orbital Prosthesis with A Smartphone Camera, Colour Calibration and Digital Printing	**Sample:** Tooth shade A2 was selected from shade guide and the LAB values were evaluated by using spectrophotometer<br><br>**Photograph:** The tooth shade was photographed along with the patients' eyes by using a smartphone camera. Cross-polarizing filter was implemented<br><br>**Colour analysis:** Adobe photoshop was used to calibrate the image based on A2 shade tab values	Smartphone camera photographs under polarizing filters can be used to match lighter shade tabs taking eye colour as reference
Moham madi, 2021 [49]	Validity and reliability of tooth colour selection by smartphone photography and software applications	**Sample:** 9 Vita Lumin Vacuum shade tabs were selected<br><br>**Photographs:** Shade tabs were photographed 10 times by using Smile lite (an intraoral photo aide).	The smartphone application attained 100% repeatability while photoshop produced more accurate results overall (P<.05)

		**Colour analysis:** Colour accuracies were evaluated from photographs taken and calibrated by a smartphone application (Chromatcher) and photoshop software.<br><br>shade tabs from 2 different shade guides were photographed under same lighting condition using the aforementioned apparatus and compared to spectrophotometer values	
Newton, 2021 [50]	The impact of tooth colour on the perceptions of age and social judgements	**Sample:** 54 photographs of human face were scored by 25 Caucasian male and female observers<br><br>**Colour analysis:** Images were shown to a computer screen and the observers were asked to rate them according to pre-set characteristics with their tooth shade<br><br>The effects of tooth colour on the subjective social judgements were evaluated from this study	Darker teeth received poorer subjective ratings when compared to lighter teeth, whereas natural teeth obtained intermediate ratings
Raza, 2021 [51]	Assessment of a smartphone-based software application as a potential digital tool in tooth shade selection: A prospective clinical study	**Sample:** 250 maxillary right central incisors and canine<br><br>**Colour analysis:** a double-blinded acceptability test was conducted using visual analysis, spectrophotometer and an-house smartphone application (shadent).<br><br>The relationship and agreement between all the methods were evaluated	64.6% agreement was found between visual analysis and Shadent software with 0.59 kappa coefficient<br><br>Additionally, 69.3% agreement was found between spectrophotometer and Shadent with 0.65 kappa coefficient<br><br>Shadent performed better for maxillary central incisors than canines
Shehadeh, 2021 [52]	Clinical evaluation of a novel radiofrequency-based toothbrush for teeth whitening and reduction of teeth stains: A pilot study	**Sample:** 12 human subjects<br><br>**Colour analysis:** Subjects were asked to use a radiofrequency (RF)-utilizing brush twice daily.<br>Tooth shade and stains were assessed using both spectrophotometer and digital photographs<br><br>Before-after colours difference were evaluated to see the effect of that brush	When compared to an unstructured mix model there was significant improvement observed in visit 5 (P<.001) and 6 (P<.001)<br><br>No significant difference was found between male and female subjects but tooth shade was significantly correlated to age (P<.001)
Soldo, 2021 [53]	Assessment of Colour Parameters on Maxillary Right Central Incisors Using Spectrophotometer	**Sample:** 30 maxillary right central incisor	The images were affected greatly under different lighting conditions with a* and b* values being

	and RAW Mobile Photos in Different Light Conditions	Photographs: Teeth were photographed by using a smartphone under different lighting conditions (frontal, lateral, with diffuser, with polarizing filters)<br><br>**Colour analysis:** CIELAB values were evaluated by using a software and compared with a spectrophotometer	mostly affected within calibrated photographs and L* on the non-calibrated photographs
Kalman, 2020 [54]	Development of a novel dental shade determination application	**Colour analysis:** An in-house application was used for colour evaluation. The application was connected with an intraoral scanner via Bluetooth.<br><br>After scanning the application will provide the RGB and LAB values of the scanned tooth	Author claimed that the proposed application can provide simple and systematic approach to perform smile designing, however, the application was limited to Apple users only
Kelkar, 2020 [55]	A comparison between visual, digital photography and polarizing filter photography for shade selection	**Samples:** 9 shades were selected from 2 different Vitapan classical shade guides<br><br>**Colour analysis:** 10 participants visually matched the shades of one shade guide to another.<br>A professional camera was used to photograph one shade guide with and without polarizing filters.<br><br>A custom shade guide was developed in Adobe photoshop software, and the observers were asked to match the shades between newly developed digital shade guide and preselected shade guide.	Statistically significant differences (P= .049) were observed between conventional visual and digital photographic methodologies<br><br>The two modifications of digital photography did not show any significant association (P=.181)
Suganya, 2020 [56]	Spectrophotometric evaluation of shade selection with digital and visual methods	**Sample:** 50 maxillary central incisors<br><br>**Colour analysis:** Shade tabs were selected by 3 methods: visual, spectrophotometric, and digital photographic method.<br><br>LAB values were extracted from the spectrophotometer as well as from photographs using Adobe photoshop.	There were highly significant differences (P<.001) observed among the methods
Rutkūnas, 2020 [57]	Accuracy of an intraoral digital scanner in tooth colour determination	**Sample:** 120 maxillary anterior teeth<br><br>**Colour analysis:** Values were extracted by using an intraoral camera and a spectrophotometer	When compared to the spectrophotometer, intraoral scanner showed 53.3% colour accuracy when recorded as VM and 27.5% accuracy for the VC shade guide.

Later on, both the obtained values were compared to evaluate the accuracy of the devices and matched with 2 different shade guides Vita 3D-Master (VM) and Vita classical (VC)

For repeatability check, all teeth were measured 5 times

Table S5: Summary of findings for Colourimetry in Dentistry

Author name (year)	Study design	Aim of study	Measurable outcomes with evaluation	Photographic apparatus used with calibrations	Certainty evaluation with Cochrane GRADEpro	Study outcomes	Novelty of the study	Clinical implications of the study
Mahn, 2020 [58]	Cross sectional study (In Vivo)	To compare colour matching and colour registration techniques using 3 different methods	• A direct visual comparison was made with an ambient light among 60 students' Maxillary right central incisors and a shade guide (A-D shade Ivoclar Vivadent AG). Colour registration was done of the matched shades and CIELAB values were evaluated<br>• Selected shade tabs were re-captured within a lightbox using WhiBal grey card.<br>• The images were saved in RAW format and photo-editing software (Lightroom; Adobe Systems Inc) was used for white balance correction. CIELAB values were measured by 'Colour Meter' software v5.11, Apple Inc<br>• For the digital image, the shade tabs were again photographed using cross-polarizing filter and CIELAB colour value were evaluated using the same software<br>• A calibrated spectrophotometer (Vita EasyShade; Vita Zahnfabrik) was used to register the colour and CIELAB value evaluation	Single Lens Reflex (SLR) digital camera D7100 Nikkon crop, with a macro lens 85mm, shutter speed 1/125, aperture F25, ISO200 and external twin flash system (Model R1C1, Nikkon crop) and for polarized image a cross-polarizing filter (Polar_eye) was used	• Moderate<br>(Colour was evaluated within same ethnic group.<br>Only labial surface of maxillary right central incisor was used)	• ΔE values between spectrophotometer and visual shade was 7.35.<br>• ΔE values between cross-polarizing filter and spectrophotometer was 6.12<br>• There was a significant difference (P<.001) present in visual method in comparison of 2 other methods<br>• There was no significant difference (P>.001) in L* and b* co-ordinates obtained from both spectrophotometer and cross-polarizing filter used image	Bland-Altman test were done for reliability check between the observers	For better accuracy in colour registration, a digital image analysis is preferable over visual analysis
Murro, 2020 [59]	Cross sectional study (In Vivo)	To evaluate whether skin and tooth colour had any influence on smile attractiveness	• Photograph of a young woman was captured showing teeth, lips and surrounding skin<br>• The images were edited in Adobe Photoshop CS5 to create shade varieties from reference skin shade guide (L'Oreal True Illusion compact makeup shades; L'Oreal) and tooth shade guide (Vita classical A1-D4 system)<br>• 16 images were created with codes and printed.<br>• 328 participants, categorized by age, sex and level of education, were screened using Ishihara chart.<br>• They were then interviewed and asked to rate the image attractiveness by using visual analogue scale (VAS)	Digital camera D7000; Nikon was used<br>Other camera details not provided	• Moderate<br>(The study was conducted within the same ethnic group.<br>There were multiple uncategorised variations within the shade guide)	• The result suggested that variation in the tooth shade was more important than skin tone for the observers. The correlation coefficient VAS Vs Skin tone and VAS Vs tooth shade were (r=0.123, P<.01) and (r= -0.431, P<.01) respectively<br>• Participants preferred a1 and a4 tooth shades more<br>• Age significantly influenced response (P=.01) while sex and level of education had no significant influence (P>.05).	Participants were initially screened for colour blindness using the Ishihara test.	Tooth shade influences smile attractiveness more than the surrounding skin colour
He, 2020 [60]	Experimental study (In vitro)	To evaluate the effect of enamel thickness on tooth colour using 3 different	• 50 extracted teeth were positioned on a custom-made stand with a Colour Checker (Extra Mini chart; X-Rite Inc) and makeshift light blocking trap for white balance and camera calibration.	Digital camera Nikon D810 with 105mm macro-lens, shutter speed 1/100, aperture F13, ISO 100, twin flash (Twin Lite MT-24EX	• Moderate<br>(Only maxillary incisor teeth were evaluated)	• No significant correlation (P<.05) was observed between enamel thickness and colour difference of NP filters	A custom-made dark box (25 × 32 × 24 cm) was used to standardize the study	Spectrophotometer and digital photography colour analysis values are not affected by enamel thickness

		colour measuring techniques; non polarized photographs, cross-polarized photograph and spectrophotomete r	• The teeth were photographed by using with and with-out cross polarisation (NP, CP) filters<br>• Pre-operative and post-operative (after demineralisation) colour analyses were done using Adobe Lightroom CC 2015 and spectrophotometer (ShadepilotTM; DeguDent)<br>• An intraoral 3D scanner, TRIOS 3 (3Shape Trios A/S) was used to measure the thickness dimension	Speedlite, Canon Inc.), flash output 1/8 was used to take no polarized method images<br><br>Cross polarized image filters were attached in-front of the camera lens with Shutter speed 1/100, aperture F11, ISO 100 and flash output 1/1		• No significant correlation (P<.05) was observed between enamel thickness and ΔL^*, Δa^* values among all 3 methods<br>• High significant correlation (P<.0001) was observed among the measuring outcomes of all 3 methods		
Albert,<br>2019 [61]	Case report (In vivo)	To carry out colour analyses using a smartphone camera	• Photographs of dental shade guide (VITA Tooth 3D-Master) and patient's teeth were taken using a smartphone camera<br>• hue and chroma were removed to produce monochromic images<br>• Easyshade spectrophotometer was used as a control group. Hue and chroma values were compared between the groups	Incomplete camera and filter descriptions were provided.	• Moderate	• Both camera-based images and spectrophotometer produced 3M1 shade for tooth restoration	Smartphones were evaluated for their clinical accuracy in prosthetic rehabilitation	Smartphone cameras can provide efficient colour analysis results without any added costs
Justiawan,<br>2019 [62]	Experi mental study (In vitro)	A colour matching system was developed for tooth-based shade recognition	• Digitally photographed images of the patients were collected from the clinic. The pictures were taken within 250-300 Lux lighting condition.<br>• 16 images were selected based on VitaPan shade guide reference.<br>• 40×40 pixels of tooth image area were cropped from intersection outcomes<br>• RGB, HSV and L* a* b* values were used to evaluate the colour characteristics during image pre-processing.<br>• 4 algorithm models were used to extract the features from the colour models<br>• 640-point data were extracted from the RGB, HSV and CIELAB values which were classified using K-nearest neighbours (KNN), Neural Network (NN) and Decision tree (DT). 640 data were divided into 480 learning data and 160 testing data<br>• Based on 3 performances; learning time, processing time and computational time the algorithms were judged for each colour spaces	No camera description was provided	• Low<br><br>(Racial and geographic dental shade variations were not considered during the design)	• *RGB evaluation*: The learning accuracy for NN was at 97.92% but took longer computational time (34 minutes). In this context KNN algorithm showed the fastest computational time (2 seconds) and had the highest testing accuracy (92.50%)<br>• *HSV evaluation*: NN showed 96.46% learning accuracy and 78.75% testing accuracy but with longer computational time (33 minutes). DT showed the fastest time (58 seconds)<br>• *CIELAB evaluation*: KNN showed 92.08% learning accuracy , 77.5% testing accuracy and computational time (0.4 seconds)	Machine learning was used to create a predictive model for tooth colour recognition from photographs	Machine learning algorithms can evaluate different colour profiles. The right algorithm with the right colour profile can be used to generate a computerised decision support system for clinical colour matching
Liu,<br>2019 [63]	Experi mental Study (In vitro)	To develop and validate an in-house 3D colour reproduction system for intraoral scanning	• *Colour profile development for scanner*: A colour chart (ColourChecker Digital SG, X-Rite Inc) with 96 colour patches and spectral reflectance were measured using spectrophotometer (ColourEye 7000A, X-Rite	_	• High	• Both intraoral scanner and 3D printer colour profiles showed good performance, but cubic polynomial showed better result (ΔE= 6.940 ranging from 1.504 to 32.660) than quadratic polynomial for printer colour profile	A colour profile for both the 3D scanner and 3D printer were developed	As of now, 3D printing colour accuracy is still limited for dental prostheses when derived from scanned images

		and 3D printing to improve tooth and gum shade aesthetics.	Inc). Based on the spectral data, CIE XYZ tristimulus values were evaluated<br>• Each patch was scanned using an intra-oral scanner (TRIOS 3, 3Shape A/A) and the digital model was saved to VRML format. Photoshop CC2014 (Adobe System) software was used to calculate the RGB values within 567,460 pixels selected area.<br>• *Colour profile development for 3D printer*: Digital model of the colour patches were cropped and reconstructed to 13×13×4 mm blocks to create a 3D colour chart.<br>• The model was printed with a 3D printer (J750, Stratasys Ltd).<br>• Spectral reflectance and CIE XYZ tristimulus value of each 3D printed box was evaluated by using spectrophotometer.<br>• For both the cases, polynominal regression based on least squares was done to resolve the relationship between the CIE XYZ and corresponding RGB values of the blocks<br>• CIE XYZ values of both colour charts were transferred into CIELAB values to evaluate the reproduction accuracy and the colour difference ΔE between the two-colour charts were evaluated<br>• Texture of the images were adjusted using 4 colour profile combinations: quadratic scanner profle – quadratic printer profle (QS-QP), quadratic scanner profle – cubic printer profle (QS-CP), cubic scanner profle – quadratic printer profle (CS-QP) and cubic scanner profle – cubic printer profle (CS-CP)<br>• For viability check, 18 resin blocks, 16 tooth shades and 2 gum shades were digitised with intra-oral scanner. According to the pre-described workflow, the models were reconstructed and printed using a 3D printer (J750, Stratasys Ltd., Israel).<br>• CIELAB values were evaluated for both original and printed samples and colour difference ΔE were calculated		(The lighting condition was not clearly described)	• Significant colour difference was observed (P<.005) between original colour chart and 3D printed chart.<br>• Group CS-CP colour profile showed the best result 8.66 ±6.94<br>• Colour difference (ΔE) of tooth and gum shades ranged from 2.1850 to 11.2156 with a mean of 6.5378 and a median of 6.3129		
Lazar, 2019 [64]	Comparative study (In Vitro)	To evaluate the influence and accuracy of polarizing filters on tooth colour	• Vita 3D-Master shade guide (Vita Zahnfabrik GmbH) was photographed in direct light and then polarized light<br>• The images were calibrated using ColourChecker Passport Photo (X-Rite) within Adobe lightroom 5.6 (Adobe System Inc)	Digital camera Canon EOS 60D with Canon EF-S 60mm marco lens, aperture F29 for direct light and F10 for the polarized light, ISO 100, macro ring flash (Sigma EM-140 DG E-TTL	• Moderate	• There were significant differences (P<.05) between polarized and non-polarized images<br>• Polarized photography was within acceptability threshold (2.7) for 23% cases while direct light exceeds the threshold	_	Cross polarizing filters in dental photography can produce accurate representations of tooth morphology

			• RGB values from the histogram were converted into L*, a*, b* values using ColourMine Org converter software	II) ¼ and a ‘Polar-eyes’ filter was used				
Lin, 2019 [65]	Experimental study (In vitro)	Proposed an automated shade matching system based on image processing and fuzzy decision techniques	• VITA 3D-Master shade tabs were photographed using a digital camera within normal clinical environment.<br>• 2 sets of images were photographed. 26 shade tab images were taken for each group and labelled as Dataset A and B for colourimetric analyses.<br>• To facilitate colourimetric analysis, RGB images were convert to YcbCr format.<br>• The images were then scaled to a fixed size 300×300 pixels and compared.<br>• S-CIELAB values of YcbCr images were used to select the teeth images, PSNR was used to evaluate the chrominance values (Cb and Cr).<br>• The images with the smallest S-CIELAB and highest PSNR values in Cb and Cr were selected for fuzzy decision system<br>• In fuzzy decision the inputs were brightness of the tabs b(n) and chrominance of the tabs c(n). To train the fuzzy technique, additional datasets C and D were used.	No camera details were provided	• Moderate<br><br>(Clinical environment was not detailed)	• The fuzzy decision technique produced a 1.92% more accurate result when compared to other studies (92.31%) with an average score of 98.74%	Fuzzy decision technique was applied with PSNR (Cb), PSNR (Cr), S-CIELAB to minimize the subjective shade matching variations	Machine learning algorithms are capable of accurately providing objective reliable decisions on shade selection
Sampaio, 2019 [66]	Comparative Study (In Vivo)	To evaluate and compare the colour differences and luminosity in different digital photography equipment’s with and with-out grey reference card	• 60 images of 10 students were captured by using 6 different photographic equipment<br>• A standard grey reference card (Genuine White Balance Reference) was put closer to patient’s teeth for further white balance correction of the capture images<br>• CIELAB values of the maxillary central incisor were extracted from original photographs by using software Classic Colour Meter version 1.8.1<br>• Afterwards, Adobe Photoshop Lightroom CC v6.0 was used to calibrate the white balance of the images and CIELAB values were extracted from the calibrated images<br>• Later on, ΔE values were calculated and compared based on their luminosity against a selected gold standard (i.e., smallest obtained ΔE value)	• Nikon D7000 (SLR) digital camera with AF-S VR 85mm macro lens (NIKKOR) and a wireless close-up 4804 R1 speedlight flash was used for all 5 groups<br>• For 2nd group with the same camera only wireless close-up 4804 R1 speedlight flash surrounded by 80gsm white printing paper extra used<br>• For 3rd group with the camera with a ring flash system Circular EM-140 DG (Nikkon) were used<br>• For 4th group wireless close-up 4804 R1 speedlight flash attached in a dual	• Moderate<br><br>(Only colour values of maxillary central incisor were evaluated)	• With and without white balance card produced lower ΔE value (ΔE =3.4) when compared with gold standard<br>• Luminosity result showed that groups with digital camera + flash attached to a dual-rigid point (P=.73) and digital camera + flash wrapping filter paper (P=.106) and digital camera + wireless close-up flash (P=.551) had no significant difference with cross polarizing filter method<br>• Digital camera + ring flash with cross-polarizing filter had significant difference (P<.001)<br>• Only grey card showed significant difference when ring flash (P=.008) or smartphone (P=.023) were used but no significant difference among the other groups (P>.05)	_	Using standard grey reference card and cross polarizing filter with digital photography can provide standardized dental colour values

				point rigid flash bracket at one side of the camera was used<br>• 5th group speedlight flash were attached with cross polarizing filters was used<br>• 6th group a smartphone Iphone 7 (Apple inc) was used<br>• For all the groups standard camera parameters were maintained shutter speed 1/125, aperture F25, film speed 200, flash (ETTL) and distance 80cm				
Maddula,<br>2018 [67]	Cross sectional study (In vivo)	To evaluate the colour reproduction accuracy of intraoral photographs with and without grey card calibration	• left lateral incisors of 10 participants were used<br>• Images were taken in RAW format and RGB values were evaluated using Adobe Photoshop CS3<br>• Conversion of the RGB values to CIELAB values was done in Caloromine software	Dgital camera Nikon D5200 was used.	• Very low<br>(Incomplete description of camera settings was provided.<br>Statistical analyses and values were not provided)	• There were differences present within colour measurements influenced by the use of grey cards<br>• The white balance correction with grey card showed more accurate result than without correction	_	During shade selection with digital photography, a grey card can be used to neutralize the image and provide better brightness accuracy
Lam,<br>2018 [68]	Experimental study (In Vitro)	Described a technique of 2D surface texture and colour mapping information to generate a 3D model	• Photographs of the following were taken: Maxillary, mandibular, occlusal, right and left buccal view<br>• An open-source photo editing software GNU Image Manipulation Program, v2.8 was used to colour calibrate the images from a reference (ColourChecker Passport,X-Rite)<br>• An intraoral scanner (True definition scanner; 3M ESPE) was used to scan the dentition and generate an STL file.<br>• Geometric registration was done to project the photographs to onto virtual model	Single Lens Reflex (SLR) digital camera EOS 500D with Canon macro lens EF 100mm, aperture F2.8 and a ring flashlight (Canon Macro Ring Lite MR-14EX) was used	• Moderate	• An open file format technique was proposed which could record RGB colours and be manipulated using CAD software.	Intraoral photographed were mapped into virtual 3D model by using geometry registration	A colour mapped virtual 3D model obtain from an intraoral image can easily evaluate colour space values
Sampaio,<br>2018 [69]	Comparative study (In Vitro)	To evaluate the effectiveness of different shade guides by using digital camera	• 5 different shade guides were used: VITA Classical (Vita Zahnfabrik) control, IPS e.max Ceram (Ivoclar Vivadent), IPS d.SIGN (Ivoclar Vivadent), Initial ZI (GC) and Creation CC(Creation Willi Geller)<br>• 11 different shade tabs: B1, B2, B3, B4, C1, C2, C3, C4, D2, D3, D4 were used.<br>• Shade guides and shade tabs were photographed 275 times	Digital camera Canon EOS 60D with 100-mm Canon Macro Lens, shutter speed 1/125, aperture F16, ISO 100 and a ring flash (MT-14 Canon Ring Flash) and a cross-polarizing filter (Polar_Eyes emulation) was used	• Low	• Vita Classical shade guide (control) all other shade guides showed significant difference in hue ($P<.05$) for B1, B2, B4, C1, C4, D2, D4 shade tabs.<br>• e.max showed the highest hue equibalance (3 matched out of 11)<br>• Vita Classical shade guide had a significant difference ($P<.05$) in chroma	_	A digital camera combined with cross polarizing filter and a ceramic shade guide can provide accurate tooth colour analysis

			• Adobe photoshop CC software was used to analyse the picture based on HSB model (hue, saturation, and brightness)			values for B2, C1, C3, D2 and D3 shade tabs.<br>• d.Sign (4 matched out of 11) presented with highest equivalence.<br>• Vita classical showed a significant difference in brightness (P<.05) for only B4 tabs when compared to the rest of the shade guides.<br>• Creation showed the highest brightness equivalence (6 matched out of 11)		
Kim,<br>2018 [70]	Experimental Study (In Vitro, Vivo)	A custom-made shade matching device was created by using support vector machine (SVM) algorithm	• 1M1, 2M2, 3M2, 4M2, and 5M2 shade tabs and their original RGB values were used to design a standard colour chart.<br>• A device was developed to measure the values by applying cross-polymerisation scheme to an intraoral camera (Whicam story 3, Gooddrs)<br>• Colour correction was performed within 3×3 colour correction matrix.<br>• A robotic arm (LBR iiwa 7 R800, KUKA) was used to standardize the image capture process. The samples were placed on a custom-made jig to a fixed position.<br>• A cap head was used to control light to 632.4 lux LED room and colour temperature 5332K.<br>• RGB values were then converted to CIELAB values.<br>• Edge detection procedure was carried out in clinic. The gingival area was first extracted by using greyscale with their obtained RGB values. The captured image was converted to binary image and 'labeling' algorithm was applied to classify the tooth area. The mean colour values were extracted and analysed<br>• The accuracy of the developed system was evaluated by applying 2 methods: Euclidean distance and SVM algorithm. The smallest Euclidean distance between measured tooth colour and mean shade colour were selected as matched shade tab<br>• *For device reliability check*: 8 similar camera devices were used for colour measurement of the shade tabs	_	• Moderate	• SVM algorithm showed higher (90%) matching rates than Euclidean distance<br>• The SVM algorithom showed 95.0 (0.36), 91.9 (0.71) and 93.5 (0.76) reliability for devices 1,2 and 3	A machine learning algorithm was developed for colour detection using intra-oral cameras	SVM algorithm-based machine learning was shown to predict colours with >90% accuracy
Yoon,<br>2018 [71]	Comparative study (In Vitro)	To compare between two digital instruments to evaluate the accuracy and	• A custom-made shade guide was fabricated from using A1, A2, A3, A3.5, and A4 no shade tabs (Vita pan Classical shade guide).<br>• For L*, a*, b* values the shade tabs were scanned, using a calibrated intraoral scanner (TRIOS Pos Colour-P13; 3Shape dental	_	• Low<br>(The shade tabs were conveniently selected	• A significant (P>.05) homogeneity of variance in the data was present between the two 2 methods<br>• Significant differences (P<.001) in L* a* b* values (especially L*) were	A comparative analysis of colour data was performed between digital scanner and shade	Though repeatability parameters were excellent, there was a lack of accuracy when

		possibilities of using intraoral scanner for shade analysis	system) with a fluorescent light which colour temperature was 6500K (FHF 32W/865, Osram GmbH)<br>• A 3D model of the shade tabs was obtained after multiple scans and was displayed on a 2880×1800pixel resolution computer (MacBook Pro; Apple)<br>• Screenshot of the scan was taken, and the image was analysed for CIELAB values using Adobe Photoshop CS6; (Adobe Systems)<br>• A colourimeter (ShadeEye-NCC; Shofu Inc) was also used to obtain the CIELAB values of the shade tabs. Under xenon arc lamp with D65 illumination to measure the tabs<br>• For repeatability check and to eliminate the inter-rater differences every measurement was taken 10 times by a single investigator<br>• A comparison was made between L* a* b* values and colour difference ΔE obtained from intra-oral scanner and colourimeter		and only includes 5 tabs.<br><br>The measurements were taken by a single observer)	present between colourimeter and digital scanner.<br>• In digital scan image and colourimetric analysis, L* and b* values strongly correlated with each other (r_L = 0.857, P< 0.05 and r_b = 0.961, P <0.05) but a significantly weak correlation was observed for a* values (r = 0.582, P <0.05)<br>• No significant difference (P>.05) was observed between Intra-class correlation coefficient (ICC) values of two methods	values obtained by colourimeter	colours were obtained using intraoral scanners
Labban,<br><br>2017 [72]	Cross sectional (In Vivo)	To evaluate the impact of gender and skin colour on tooth shade selection	• One adult male and one female were photographed smiling<br>• The subject photographs were modified according to "Fitzpatrick" scale into 4 colours (fair type II, medium III, olive IV and brown V). 48 images were generated from the modification<br>• 6 shade tabs (BL1, BL2, BL3, BL4, A1, and B1) were taken from 'A-D shade guide' (Ivoclar Vivadent Inc) and photographed separately and merged to create a virtual shade guide<br>• 336 participants (60.4% male and 39.6% female) were appointed to evaluate the most aesthetically acceptable tooth shade based on gender and skin colour from the images	Digital camera Nikon d7100 with shutter speed 1/80 and aperture F8.0. and 2 external flashlights were used (Neewer 60×60 cm/2400×2400)<br><br>For teeth samples, same camera setting was used with an USM marco lens	• Low<br><br>(Teeth shades were conveniently selected.<br><br>Focused on only one ethnic group.<br><br>Different lighting conditions and background were used while taking the photographs)	• Tooth shade selections varied significantly (P≤.05) for male and female skin colours<br>• Lighter tooth shade with lighter skin colour was preferred (P<.05) by the female observers<br>• Significant association was observed between education level(P=.036) and monthly income (P=.009) of the observers when selecting acceptable shades	Fitzpatrick reference skin scale was used to standardize skin colours	Skin colour in photographs can dictate which tooth shade is perceived as acceptable by the general population
Luo,<br><br>2017 [73]	Experimental study (In Vivo and In Vitro)	To evaluate the ability of a custom-made digital imaging system for measuring tooth colour and whiteness	• To check the repeatability of the system, 2 sets sample were used. The first set comprised of a 16 shade tabs (Vita Classical shade guide) and the second set comprised of 14 extracted human teeth<br>• The sets were photographed using VDIS system at the baseline, 5min from baseline, 2 hours,1 week and 2 weeks.<br>• For the validation check, upper central incisor of 33 male and female volunteers were photographed by two operators at the baseline, 5min and 120min interval.	The system consisted of a commercial Single-lens reflex (SLR) camera with a ring light (CCS Inc), a diffuser filter (CCS Inc), 2 polarizing filters (CCS, Inc) were placed in-front of the camera.	• Low<br><br>(The shade guide was conveniently selected.<br><br>Incomplete camera description was provided.	• In vitro measurement: ΔE*<1 unit. ΔWIO between 1-2 unit<br>• In-vivo measurement process, CIELAB and ΔWIO showed 82% and 99% variability<br>• Operator variability was less than 0.5% and overall measurement error was 0.3%	A Video-based imaging system (VDIS) was developed	Controlled digital photography can produce reproducible tooth colour analyses

			• A graphic-user-reference was developed to analyse the images based on region of interest and the extracted RGB, L* a* b* and tooth whiteness index values (WIO). As a reference model, ColourChecker chart (VeriVide) was used.<br>• Repeatability and reproducibility of the in-built VDIS system were measured based on the obtained RGB, L*a*b* and WIO values	A white ceramic tile was used to monitor the white balance	For in vivo analysis only central incisor was used as sample)			
Mehl,<br>2017 [74]	Comparative study (In Vivo)	To compare a 3D scanner with traditional visual and digital colour measurement techniques	• Colour measurements of 40 maxillary central incisors and canine were evaluated. Colour measurements were taken in 7 ways. Group 1-2 underwent visual colour analysis while groups 3-7 underwent digital colour analysis<br>• Group 1: One dentist used the Vita 3D-Master with bleached shade guide (Vita Zahnfabrik) and visually matched the shade tabs with the respected tooth.<br>• Group 2: dental technicians also followed the same technique to visually match the colour by using 3D-Master shade guide (Vita Zahnfabrik).<br>• Group 3: used Trios 3 Shape device (Trios Colour,3Shape) to scan the surfaces of the respected tooth. Colours were measured using 'colour prick' from 3D model.<br>• Group 4: used a calibrated Vita Easy shade Spectrophotometer device (Vita Zahnfabrik) for colour value evalaition.<br>• Like group 4, Group 5 used Vita easy shade spectrophotometer but newer generation (Easyshade Advance 4.0, Vita Zahnfabrik) for colour measurement<br>• Group 6: also used spectrophotometer but from a different manufacturer, SpectroShade device (MTH Optic Research AG) in which, after photo-calibration, the target teeth were recorded at 90º angle with an optic hand piece.<br>• Group 7: used SpectroShade Micro device (MTH Optic Research AG) to evaluate the micro colour of the target tooth<br>• All 680 measurements were taken within same environment and colour measurements were taken. The colour difference ΔE and accuracy were evaluated	_	• Moderate<br>(The teeth were conveniently selected for colour analysis)	• A significant difference (P<.05) was observed between the groups having easy shade and easy shade advance spectrophotometric method.<br>• SpectroShade Micro (Group 7) showed significantly more accuracy (P<.05) than the other methods<br>• Repeatability measurement among the digitally evaluated colour devices did not differ significantly (P>.05)	_	A 3D scanner can provide more accurate surface data and colour measurements than the other digital colour analysis devices<br>Spectrophotometers are victims of inter-device result variability
Miyajiwala,<br>2017 [75]	Comparative Study	To compare and assess 3 different shade selection	• 50 right maxillary central incisors were used for colour evaluation. Colour was visual	Single-lens Reflex (SLR) digital Camera Canon 500D attached with a Harison	• Moderate	• There was significant agreement (Kappa=0.204, P<.01, Z=-3.2) among the 3 colour evaluation methods	For the colour blindness check Munsell colour test and pseudochromatic	Observers can identify and differentiate certain tooth shades better than

	(In Vivo)	methods within a clinical environment	analysed, spectrophotometrically, and digital photography method.<br>• The procedures were carried out between 11:00 am and 1:00 pm on a clear day<br>• The subjects were photographed with an 18% grey reference card. Image analysing software Adobe photoshop CS,8.0 was used to evaluate the CIE L*, a*, b* values<br>• For visual colour analysis VITAPAN classical shade guide (VITA Zahnfabrik) was used to select the appropriate shade tabs based on the hue and chroma values<br>• Spectrophotometer VITA Easyshade, (VITA Zahnfabrik) was used to evaluate the L*, a*, b* values	tripod stand (3D EV). Camera aperture and automatic white balance, ISO 125, a flash TTL attached on a was used	(Only the middle third of the maxillary right central incisor was evaluated.<br><br>Daylight regulations were not described)	• Digital photography and spectrophotometer showed higher portion of agreement (Z=-3.2) when they were compared to evaluate the similarities.<br>• Higher significant difference (P<.01) was observed between spectrophotometer and digital methods of having ΔE value <2<br>• A higher percentage of observers agreed with spectrophotometer on A1 (20%) and D2 (10%) shades	colour test were done by an experienced ophthalmologist	others, which may be comparable to spectrophotometric values
Tam,<br>2017 [76]	Experi mental study (In Vitro)	A machine learning method with smartphone camera was proposed to facilitate dental colour matching,	• A 3D-Master (Vita Zahnfabrik) shade tab was used and labelled in numeric order from 1 to 26.<br>• The shade guide was photographed inside a dental clinic and divided into 2 groups.<br>• For group 1, 40 images were captured with different illuminations in different parts of the clinic.<br>• To minimize the effect of different illumination changes within group 2, a fixed position was set, and the shade tabs were again photographed for 10 times.<br>• All the pictures were converted from JPEG file to bitmap file for further processing<br>• The selected shade areas were cropped for each image. Whole content was divided into small blocks (10×2) to compute the colour distribution. RGB, HSV, XYZ and L, a* and b* values were extracted from the blocks and transformed into a row vector structure<br>• Support vector machine (SVM) was used as a classifier. For this purpose, one of the previously photographed samples were used as a test shades and others were used as training samples.<br>• For top classification accuracy, only top 1 and 3 shade matches were considered	Smartphone camera iPhone 6 plus (Apple,USA) with automatic capture mood, Shutter speed, aperture, ISO, White balance was enabled.<br><br>Light tube 4000K colour temperature was installed into a sunlight protected room	• Moderate<br><br>(A colour temperature meter would be needed for doing SVM colour analysis because a constant illumination is required)	• For group 1, one and three classification accuracies were (0.86 ±0.1 and 0.98 ±0.4)<br>• When 15 shots per set was used the accuracy was 0.90 but when 40 shots per set was used than it reached into higher accuracy level 0.98.<br>• In case of group 2, top one three accuracies were 0.97 and 1.00. The standard deviation of the classification was decreased when the training sample comprised.<br>• Compared to group 1 for higher accuracy group 2 required lower sample size for SVM classification<br>• Both the groups showed excellent intraclass correlation, but group 2 (r=0.99) exhibit better reliability than group 1(r=0.94)	Support Vector Machine (SVM) learning was used in digital shade matching	Fewer photographs fed into machine learning can yield accurate colours when lighting conditions are controlled.<br><br>Deep learning is also capable of overcoming mismatched illumination albeit with a loss of accuracy
Carney,<br>2016 [77]	Experi mental study (In viro)	To validate the accuracy of digital imaging system using RGB model	• 35 shades containing VITA Linearguide 3D Master and 15 shades containing VITA Bleached Guide 3D Master shade guides were photographed and RGB values were extracted (using ImageJ)	Canon Rebel T3i camera was used with a flashlight Canon Ring Lite MR-14EX and ¼ flash output, shutter speed 1/200, ISO 200,	• Moderate<br><br>(Measurements were taken with a limited range of area)	• Regression model provided high proportion of variance (R^2>.99) in reflectance<br>• High intraclass correlation coefficient was observed in RGB values (R=0.84, G=0.97, B=0.97) during multiple measurements among the same shade	Relationship between digital RGB values and lightness of shade was established using radiant energy	Accurate tooth colour can be obtained from digital imaging

			• Radiant energy was measured using spectroradiometer within 380mm to 780 mm Spectrawin 2.0<br>• Regression models were used to convert the RGB value to Commission Internationale d'Eclairage XYZ tristimulus values with D65 illumination and 2º observer.	aperture F22 and focal length 0.39<br><br>Radiant image: (PR 705) with a Xenon arc lamp (300W; Oriel Instruments) connected with a fiber optic light cable in a 45º angle.		tabs. Which indicates high reliability present in R values and little variability in G and B values		
Gurrea, 2016 [78]	Case-control study (In vitro)	To digitally evaluate the colour variability between different shade guides considering hue value	• 125 photographs of VITA classical (VITA Zahnfabrik) (Control), IPS e.max Ceram (Ivoclar Vivadent), Iitial ZI (GC), Creation CC (Creation Willi Geller) were taken<br>• Hue, saturation and brightness (HSB) were evaluated using Adobe Photoshop CC software	Single Lens Reflex (SLR) digital camera Canon EOS 60D with 100-mm Canon Macro Lens, Ring flash (MT-14 Ring Flash, Canon), Shutter speed 1/125, aperture F16, ISO 100 was used. Camera was set Adobe RGB colour space mood and a cross-polarizing filter (Polar Eyes, Bioemulation) was used	• Very low<br><br>(Shade tabs were conveniently selected)	• The results showed that none of the VITA -coded shade guides properly matched chroma or hue with VITA Classical shade tabs.<br>• Results suggested that shade selection should be made by using ceramic shade tabs	A cross polarizing filter was used to remove specular reflection from various shade tabs	There is an evident mismatch among the different commercially available shade tabs
Hein, 2016 [79]	Experimental study (In Vitro)	To evaluate the colour changes caused by different diffusers and the effectiveness of grey reference card for colour correction	• 40 extracted, unrestored teeth were selected and positioned perpendicularly by using an ABB grid pattern (LEGO)<br>• The teeth were photographed using 5 commonly used diffusers; Polyethylene (PET), White Forest photographic paper (ProTech Lighting), Mini SoftBox polyamide material (LumiQuest), 80gsm white printing paper, 3M linear polarizing sheet<br>• A standard grey reference card (WhiBal, Micheal Tapes Design) was used for white balance correction<br>• The RAW images were transferred into Adobe Lightroom CC and the images were colour corrected and also CIELAB value were evaluated	Digital camera Canon EOS 5D MKII with shutter speed 1/125, aperture F32, ISO 100 and twin flash Canon MT-24EX Macro light was used.	• Low<br><br>(The sample characteristics were not clearly described)	• White Forest (ΔE=1.24), LumiQuest (ΔE=2.94), PET (ΔE=6.55), 3M linear polarizing sheet (ΔE=7.58) all caused visual colour change<br>• Grey reference card Improved colour differences for White Forest (ΔE=0.58),LumiQuest (ΔE=0.93), PET (ΔE=0.66), 3M linear polarizing sheet (ΔE=0.53)	A custom grid-like stand was designed to house the teeth and reference card to standardize the photographs	Choice of professional diffuser affect the colour accuracy of the photographed tooth. Grey cards can mitigate some of the colour differences present
Culic, 2014 [80]	Experimental Study (In vitro)	To evaluate tooth colour parameters from a digital image using in-house software	• VitaPan 3D master shade tab was photographed<br>• In-house software (TooDent) was used. Image was calibrated every time by selecting 2M2 shade tab as reference and area selection with photographic reflection exclusion was done automatically<br>• 2 sets of CIELAB were obtained by using software as well as with spectrophotometer Vita EasyShade Advanced.	Digital camera Canon 400D, Canon 100mm lenses and CanonMR14-EX ring flash-light (5500K) with shutter speed 1/125, aperture F22, ISO 100, Resolution 3888×2595 pixels	• Low<br><br>(The reference shade tab was conveniently selected.<br><br>Environmental factor control methods were	• 80.77% ΔE values were below the reference threshold level of 3.2<br>• There was a strong correlation (r=0.914, P<.001) between the in-house tool and the predefined shade tab values	A novel in-house software was developed to evaluate dental photographic colour accuracy	A well-calibrated in-house software can accurately analyse colours within dental photographs

					not adequately described.)			
Montero,<br>2014 [81]	Cross sectional study (In Vivo)	To evaluate the influence of lightness of tooth colour on social attractiveness using photographs	• 2 subjects (Male and female) with straight maxillary anterior teeth were photographed full face using a digital camera<br>• For both the subjects, original photographs were used as a natural smile sample<br>• Photoshop Cs6 (Adobe Inc) was used to create the other photographs by increasing or decreasing the exposure by 0.1 unit. RGB values were converted into CLELAB colour values<br>• 7 copies of each photograph were laser printed (21cm×30cm) and distributed among 42 dental students<br>• Participants were asked to answer the questionnaire which extracted data related to sociodemographic, behavioural, and self-rated dental condition of the participants. Afterwards, the observers were asked to make judgments on the test subject's social appeal from the photograph	Single Lens Reflex (SLR) digital camera Canon EOS-400 (SLR) with a tripod with-out flash within a natural lighting condition was used	• Very Low<br><br>(Full description of the camera setup was not provided.<br><br>Ethnic variations were not considered.<br><br>Dental student observers were used that may not represent the general population)	• ΔE values for darker to natural tooth colour were 8.5 for females and 6.4 for males. And ΔE values for natural to lighten tooth colour were 9.9 for females and 8.6 for males<br>• Gender within the photograph was not a significant factor ($P > .001$) for Social Appeal Scale (SAS) but in case of relationship competence (i.e., one of the scoring criteria of SAS) female photograph showed higher relationship competence (2.4±0.9) than the male (2.3±0.9) when the tooth lightness was natural<br>• SAS was more correlated with psychological competences, relationship satisfaction and social abilities ($r = 0.87, 0.84, 0.83$; $P < 0.01$)	The impact of the dental appearance based on social judgment was evaluated by using Social Appeal Scale (SAS)	Proper light exposure on the teeth can influence the perception of observers on the overall attractiveness and social status of the person
Tam,<br>2012 [82]	Comparative study (In vitro)	To compare the colour of the shade tabs by using appropriate morphology and colour texture of the tooth with a digital camera	• A 3D- Master (Vita Zahnfabrik) dental shade guide was used and each tab was photographed 10 times<br>• The images were cropped manually using Matlab (Matlab R2007b) software.<br>• light reflection was removed by converting the image to greyscale.<br>• The entire content was divided into (10×2) small boxes to compute the colour distribution<br>• 2 device dependent (RGB, HSV) and 2 device -independent colour spaces (XYZ, CIELAB) were evaluated by plotting them into different equations	Single Lens Reflex (SLR) digital camera Canon EOS 1100D with shutter speed 1/200, aperture F25, ISO 100, a ring flashlight Sunpak auto 16R pro (SEA&SEA SUNPAK) colour temperature 5500-5600K within a dental clinic was used	• Low<br><br>(The shade tab was conveniently selected)	• There was higher accuracy in CIELAB and HSV (0.75 and 0.67 respectively) over RGB (0.55) and XYZ (0.50)<br>• When individual colour spaces were compared, b* (0.80) from L*a*b* and S (0.75) from HSV presented with higher accuracy<br>• Stability of the camera apparatus in individual shoots ranged from 0.73 to 0.96<br>• Average accuracies were obtained for darker shades were higher 0.99	_	Colour accuracy is camera device dependent but can also be influenced by other factors such as image stabilisation and shade guide selection
Lasserre,<br>2011 [83]	Comparative study (In Vivo)	To compare the reliability of an intra-oral cameras in determining accurate colours by comparing against visual and spectrophotometric methods	• 76 right central incisors and canine teeth were analysed by 3 observers for colour detection<br>• The teeth were analysed for 2 consecutive days under 700 lux light intensity and 3750K colour temperature by using the 3 methods<br>• *1st method:* visual colour selection was done by using Vita 3D-Master Shade Guide, under a daylight illumination device (Optilume True Shade; Optodent) at 1650 lux light intensity and 3800K colour temperature	_	• High<br><br>(A small number of observers were selected for colour evaluation)	• The correlation coefficient agreement was high and significant ($P < .01$) among 3 examiners for True Shade lamp and Sopro intraoral camera<br>• For all 3 methods inter examiner reliability was higher only for canines ($P < .01$)<br>• Correlation co-efficient was lower for spectrophotometer for methods 1 and 2<br>visual method<br>• In 1st and 2nd visual analysis method significant agreement ($P < .05$) was	Observers' colour vision was examined by desaturated test of Lanthony (Desaturated Farnsworth Panel D-15) prior to the study	The use of chairside intraoral cameras was shown to produce relatively similar diagnoses of tooth colour when compared to other conventional methods

			• *2nd method:* Colour selection was done using Sopro 171 intraoral camera at colour temperature of 6500K. Hue, Chroma and Value of the imaged teeth were visually compared with the Vita 3D-Master Shade Guide<br>• *3rd method:* Reference colour data were obtained from the same tooth area, using Vita Easyshade Spectrophotometer at D65 illumination. Basic tooth colour was determined by "ToothSingle" menu option			found for both canine and central incisors		
Tung, 2011 [84]	Experimental study (In Vitro)	To evaluate the influence and effectiveness of camera white balance setup in dental photography	• 1.0 mm thick 2 sets of ceramic disks, consist of 15 shade tabs were fabricated with Vita VMK 68 dentin porcelain (Vita Zahnfabrik) according to manufacturer's instructions.<br>• All 15 shade tabs were photographed under two separate white balanced conditions: auto white balance (AWB) and camera white balance (CWB).<br>• Images were projected on a 24-it resolution screen. Image were then cropped to 10mm area and RGB values were converted into L*, a*, b* using Adobe Photoshop 6.0 software.<br>• The areas were measured 3 times and mean values were recorded by using histogram function of Adobe Photoshop. Images with the shade tabs were used as digital shade guide<br>• For reliability check, this procedure was carried out 5 times leading to 15 images being taken for each condition<br>• To validate the digitally generated shade guides, L* a* b* values of each shade tab were compared to a spectrophotometer (CM-508, Konica Minolta Sensing)<br>• For applicability check, 10 other operators were asked to match the ceramic shade guides with the digital shade guides. The least colour difference ΔE between the test specimens and 15 reference guides were calculated.	Single-lens reflex (SLR) Digital camera Nikon D1 with 105mm macro lens (AF Micro-Nikkor, Nikon),ISO200, 2 ring flashes (Nikon Macro Speedlight SB-29 TTL, Nikon), a custom made LED illuminator with 12 white LEDs (SDL-5N3PW-S, Sander Electronic) were used for CWB calibration.<br><br>According to manufacturer's instructions CWB was manually pre-set by shooting a white standard plate (no. 91547, Tokyo Denshoku)<br><br>Characteristic of the light sources were analysed by using a Spectrophotometer (USB200, Ocean Optics) and a chroma meter (CL-200, Konica Minolta Sensing)	• Moderate	• A significantly high correlation coefficient (r >0.96, P<0.001) was found for ΔE between spectrophotometer values and the shade tabs generated in CWB setups<br>• No significant correlation (r =0.038, P=0.483) was found for a* values when comparing spectrophotometer with CWB and AWB setup<br>• The operators' colour matching ability improved from 67% in AWB to 93% (P<.05) when using CWB	A custom-made LED illuminator with 12 LED white lights was used to make a circular frame for shade photography	Manual/custom camera white balance can aid in more accurate shade classifications
Lindsey, 2010 [85]	Cross-sectional study (In Vivo)	Evaluated the influence of facial colour on the perceptibility and acceptability threshold of tooth colour	• 2 portrait images of full maxillary central incisors of 2 different people smiling were edited in Photoshop (Adobe Systems, Inc).<br>• The pictures were edited in following way: left central incisor was replace by the mirrored image of right central incisor, specular reflectance was adjusted, the colour balance was adjusted based on the average standard CIELAB values of central incisor, images	_	• Low<br>(Only the maxillary right central incisor was modified.	• Perceptibility and acceptability for each portrait colour space was almost identical<br>• Colour difference threshold was in between (ΔE=1.45-2.9)<br>• Power analysis showed a colour difference of ΔE=0.35 which was 80% statistically significant (P<.05)	The colour vision of the participants was checked by pseudo-isochromatic plate test	Tooth colour analysis can be done by using digital photograph but is subjected to observer perception and acceptability

			were placed against 5500k achromatic backgrounds with 1024 x1024 pixels<br>• The images were screened in a computer display (19” RGB monitor, 24D Diamondtron, 1600 h x 1200 v x 80 frames/sec x 8 bit per colour channel) for perceptibility and acceptability check<br>• 1000 trials were made in pseudo-random order among 10 randomly selected observers.<br>• The trial included 32 separate conditions: 2 portrait types (Caucasian and African American) x 5 ΔE^* values within 1.0-5.0 x 3 CIELAB directions (L*, a*, b*) for each image.<br>• Beginning of each trail participants were viewed a grey screen for at least 2.5 seconds<br>• The participants underwent 4 sessions of perceptibility and acceptability check, and each session was for <30 minutes. In first 2 sessions, participants were asked to indicate which central incisor looked different according to their perception of colour. In the second 2 sessions, they were told to select the teeth more acceptable to them.		The study was conducted between 2 specific ethnic groups)	• Higher L* value was observed for Caucasian image		
Denissen, 2010 [86]	Experi mental study (In vitro)	To develop a ceramic shade tab and analyse its photographic colour accuracy	• 5 Shade tabs were made with 1.5mm thick glass ceramic using CEREC3 scan milling.<br>• The shade guides and teeth were photographed<br>• Daylight spectrum was measured by using PC2000 Spectrometer (Ocean optics)<br>• Adobe Photoshop CS2 software was used to measure L*, a*,b* values of both the teeth and shade guide within different tolerance setting (6,8,10,14,16)	Canon EOS D30 camera with les EF 100mm/FI 2.8 35^{0}angle, shutter speed 1/125, aperture F16, ISO 80, 2160×1440 pixels<br><br>White balance and noise reduction on, no polarizing filter and Nikon Flash power 1/8 were used<br><br>A white tile (WS-2 Top Sensor System, Eerbeek) 32mm diameter and 10mm thickness was used reduce light reflection	• Low<br><br>(Only measured the colour of the premolar tooth)	• Precision error was between 0.602 and 2.179% for shade guides at different tolerance level<br>• Error for premolar was between 0.488 and 1.773%.<br>• Tolerance level 6 pixel showed significant difference with all shade guides (P>.05).	5 ceramic shade tabs were made for computer-based colour analysis with different tolerance levels	Custom made shade tabs produce clinically acceptable colour variances (ΔE<0.5) when evaluated using digital photography

Caglar, 2010 [87]	Comparative study (In vitro)	To compare the digital colour values obtained from ceramic and composite shade tabs against conventional colourimetric technique	• The following 2 composite shade guides were photographed: Charisma (Heraeus Kulzer) and Premise (Kerr Corporation, Orange)<br>• The following 2 ceramic shade guides were photographed: Vita Lumin Vacuum (VITA Zahnfabrik) and Noritake (Noritake Co)<br>• *1st group of photographs:* taken using 4 fluorescent lamps Philips PL-C 18 W/827 (Koninklijke Philips Electronics) at a temperature of 2700K.<br>• *2nd group of photographs*: taken using 2 fluorescent lamps attached to the lower socket of the stand and 2 lamps attached to the upper (Philips PL-C 18 W/865, 6500K lower, 2700k upper)<br>• *3rd group of photographs:* taken using 4 fluorescent lamps at 6500K temperature<br>• For each shade guide, 3 images were captured and colour value were evaluated using Adobe Photoshop CS2, (Adobe System Inc)<br>• EasyRGB software (Logicol S.r.1) was used to convert the RGB values to CIELAB values and compared with a colourimeter ShadeEye NCC Detal Chroma Meter (Shofu Inc.)	Dgital camera Fuji S20 Pro (Fujiflim) with auto white balance, shutter speed 1/80, aperture F11 and 4 fluorescent lamps 1200lm with temperature (2700 K, 2700–6500 K, and 6500 K) were used	• Moderate<br><br>(Shade tabs were conveniently selected)	• L* values from both shade guides did not exhibit significant colour differences (P>.01) at all colour temperatures<br>• At 2700K temperature, a* and b* values for digital imaging did not show significant colour difference (P>.01) but within 2700-6500K and 6500k temperature the values showed significant difference (P<.01 and P<.001)<br>• Colourimeter showed significantly lower L* values (P<,01) than the photographic technique<br>• L* and b* values obtained from colourimeter and digital imagining both showed high levels of correlation<br>• a* values showed high levels of correlation among the shade tabs (except Charisma composite shade guide)	–	Colour temperatures above 2700K significantly affect the accuracy of red and bluish colours present within the dental shades. This is because the shade tabs are composed of different filler particles that reflect more of the predominant tints from the shaded surface
Schropp, 2009 [88]	Comparative Study (In vitro)	To evaluate the effectiveness of photographic image and graphic software on colour analysis against conventional technique	• 12 tabs from a shade guide (Vita 3D-Master Guide) were used as test tabs<br>• In the 1st part, the test tabs and matched shade tabs from another Vita shade guide were placed side by side and photographed in natural light with grey background and within 3 types of flash setting<br>• ¼, 1/32 and 1/8 setting of flash were used to take images so that conditions like overexposed (OE), underexposed (UE) and well exposed (WE) images could be captured at the same time under same environmental condition<br>• By using Paint Shop Pro (PSP) V9.0 graphic software six images of OE, UE and WE were merged into one file and arrange into 3 rows and 2 columns<br>• By viewing the image into 17-inch LCD display (ViewSonic VG700d) 9 observers selected two shade tabs with identical colour	Single Lens Reflex (SLR) digital camera Canon EOS 20D (Canon USA Inc) with marco lens 100mm, shutter speed 1/125, aperture F22, ISO 200, a ring flashlight (Canon Macro Ring Lite MR-14EX) was used	• Low<br><br>(The shade tabs were conveniently selected)	• 8 out of 12 shade tabs were correctly matched (67%) in the graphics software<br>• Shade matching capability was significantly better (P<.02) for graphic than visual analysis or digital photography<br>• No significant difference (P>.02) was found between visual shade matching performed in clinic or by digital photographs<br>• A significant correlation (P=.01) was found between time required and colour shade when visual analysis was done to determine correct colour shade	• A phantom head with Columbia Detoform model was used	Human perceptibility of tooth shade is influenced by the type of shade and time given for analysis. Such limitations are not seen for computer driven colours

			codes. The image selection and time spent for assessment were recorded.<br>• All test tabs and matched shade tab colours were checked using Spectrophotometer (Vita Easyshade, Vident)<br>• In part 2 of the study the ability of the observers to visually match the tabs were evaluated. For this A phantom model with Columbia Dentoform model (S562, Columbia Dentoform Corp) was used. The test tabs were placed into phantom head and within a standardized daylight lamps (Elipse U3 EL-4,000◦ K, D-TEC AB) and colour temperature 4,800ºK they are photographed. The observers were asked to match the colour and time required for the selection were recorded.<br>• After 2 months of the 1st session the test was repeated on an alternative group of 12 shade tabs. Observer was asked to choose lightness group, Chroma, and R, L hues<br>• Lightness, chroma and hue were analysed using graphic software PSP.<br>• ΔE values between the test tabs and 26 shade tabs were calculated.<br>• The measures were taken after 4 weeks of interval					
Smith,<br>2008 [89]	Experimental study (In Vivo and In vitro)	To assess the reproducibility of a digital imagining system (DIS) used for tooth colour measurement	• For in vitro assessment, 10 extracted incisors/canines with different colours were positioned into the DIS system with black background and photographed 3 times by 2 operators each<br>• DIS system consist of a metal frame with 4 daylight halogen lamps 50W SoLux D65 (Outside-in Ltd), a polarizing filter 105mm positioned at 90º angle.<br>• Surrounding metal frame was fitted with 4 UV fluorescent light tubes (Lighting Technology) at 30º angle and a professional camera was attached with the frame<br>• For in vivo, 2 studies were conducted. For study 1, 25 healthy male and female subject's upper anterior teeth were photographed for 4 consecutive days by 3 operators to check the day-to-day variation. 2 images from one subject were used to check operator reliability and intra-operator repeatability.<br>• For study 2, 11 healthy subjects were photographed twice a day for 3 days by 3 operators. When the light had been	Digital camera Kodak SLR/N 14 Mpixel (SLR, Eastman Kodak Company) with a 90mm macro lens (Nikkon) was used.<br><br>The camera was connected with a BelkinIEEE 1394 Fire-Wire PCI card (Belkin International INC.) and Sony Viao Laptop (Sony crop)	• Moderate<br><br>(The camera calibration information was not adequately provided)	• CIELAB value recorded by the operators for DIS: L*= 64.81-67.16, a*= 6.56-6.99 and b*= 26.30-29.72.<br>• L* and a* values were within the range of previously reported non-contact camera-based system studies but the b* value was slightly higher because of variations in lighting<br>• Pairwise correlation and P values showed excellent agreements between In vitro and in vivo data (r>.9)<br>• On day 1 and 2, in vivo study experiment for operators 4 and 5 showed excellent agreements Operator 1, 4 and 5 showed lower correlations (r <0.9 but >0.7).<br>• on day 3, all operators showed excellent agreement (r ≥ 0.9)<br>• Intra and Inter-operator variability was not significant (P>.05) among the 5 operators	• A mobile version non-contact-camera based DIS was developed	A well-designed apparatus can take visually acceptable, colour accurate dental photographs. However, a learning curve accompanies, that varies between operators

			illuminated for a fixed time the subject's teeth image were taken<br>• The images were analysed in Adobe Photoshop CS2, V9 (Adobe system Inc) and the RGB values were converted into CIELAB values and CIEWI (Whiteness Index) values. Values were standardized against ceramic white tile colour standard (Ceram) by using algorithm					
Wee,<br>2006 [90]	Comparative study (In viro)	To evaluate the colour accuracy of three commercially available digital cameras by applying various colour calibration models	• The following shade tabs were used; 264 colour patches from Q60 card; 65 shade tabs including 16 tabs from the Vita Lumin Vacuum (Vident Inc.), 26 tabs from 3D Master (Vident Inc.), 3 bleaching shade tabs (Vident Inc), and 20 tabs from Chromascop (Ivoclar, Vivadent)<br>• For colour measurements an experimental setup was developed. For stability check, a white tile (SW) with known spectral value were evaluated for 10 mins and 24 spectral measurements were taken.<br>• Initial incident light was evaluated by plotting spectral reflectance of known SW measurement and measured value of reflected light at 'zero' time within an equation<br>• For accuracy check, 24 colour patch (Mini Colour Checker, GretagMacbeth) measurements were carried out and compared with the manufacturer-provided data using linear regression<br>• For repeatability check, 7 dental shade tabs were chosen from the 65. 22 patches were also selected from 264 colour patches<br>• Colour measurements were taken 3 times in a random order<br>• Shade tabs and colour patches were digitally photographed by using 3 commercially available digital cameras<br>• *The experimental setup*: A camera was set up in place of the spectroradiometer and used to capture 987 images<br>• Images were converted to tiff format, cropped and RGB values calculated using software Adobe Photoshop 7.0 (Adobe Systems, Inc)<br>• 4 calibration models and 3 regression models were developed by using RGB values from 264 colour patches for each optical parameter to convert it in CIELAB values	3 commercially available Single Lens Reflex (SLR) digital camera:<br><br>1)Nikon D100 (Nikon Inc) with 105mm CCD macro lens, shutter speed 1/400, aperture F5, ISO 200, white balance direct sunlight.<br><br>2) Canon EOS D60 (Canon USA Inc.) with 100mm CMOS macro lens, shutter speed 1/125, aperture F4.5, ISO 200, white balance sunlight.<br><br>3) Sigma SD9 (Sigma Corporation of America with 50mm Foveon macro lens, shutter speed 1/125, aperture F6.7, ISO 200, white balance sunlight were used	• Moderate<br><br>(The cameras, shade tabs and colour patches were conveniently selected)	• The stability of the colour measurement setup showed mild system error and light source instability.<br>• There was a high correlation (r= 0.977) between measured and manufacturer reported CIELAB colour values. No significant differences (P=.3988) were observed at 'zero' time measurement values.<br>• All cameras with the current calibration model showed significant difference (P=<.0001) except Nikon D100 with PRM3 model which had colour accuracy of 1.79 (ΔE=0.09, P=.0039)	A spectroradiometer (PR 705, Photo Research Inc.) and 2 D-65 desktop lamps (Sol-Source, GretagMacbeth) were attached to an optical table (Mecom Inc) with 0º and 45º illumination. Spectral reflectance for all the measurements were set up from 380 to 780nm.	Camera calibrations, lens selection and illumination sources all affect the degree of colour accuracy, irrespective of the shade tab or skin patch chosen

			• The accuracy of the calibration model was determined by the colour difference obtain between predicted and measured shade tab colours					
Jarad,<br>2005 [91]	Comparative Study (In vitro)	To compare the accuracy of visual perception in conventional tooth colour matching against digital photographs	• Vita Lumin shade guide (VITA Zahnfabrik) was used of shades A2, A3, A3.5, B2, B3, B4, C1, C2 and C3<br>• A dental phantom manikin was attached with a tripod of 1.6m height.<br>• Each of 10 observers were asked to accurately match 27 shades, based on visual perception (n=270)<br>• The matched shades were analysed (using spectrophotometer) and compared against digital photographs.<br>• CIE LAB colour values were obtained from Adobe Photoshop Software, v.5.5. contingency and correlations between the conventional and computerised techniques were evaluated	Digital camera Nikon Coolpix 990 (Nikkon) with shutter speed 1/60, aperature F9, ISO 100, focal length 15.5m, a ring flash (SB-21b Ring Flash, Nikon) was used	• Low<br>(The shade tabs were conveniently selected.<br>The observers had varying experience in restorative dentistry)	• There was a statistically significant (P<.001) difference between conventional (43%) and computerised (61.1%) techniques	A phantom manikin was used to simulate clinical colour matching	Visual perception of accurate tooth colour is subjective and is prone to experience-based variations. Digital photographs were shown to be slightly more reliable
Cal,<br>2004 [92]	Comparative Study (In vitro)	To investigate the reliability of graphics software in evaluating the colour accuracy of photographs	• Adobe Photoshop 4.0* was used to create an image with red, green, blue, yellow and white circles for colour analysis reliability check<br>• Chromascop (Ivoclar Vivadent) shade guide was photographed against a black background in daylight.<br>• 3 shade guides from same manufacturer (X, Y and Z) were photographed against studio white background<br>• Image were analysed in Adobe Photoshop 4.0 software within 74pixel diameter and the L, RGB value were calculated	Camera fixed on a tripod within daylight and saved in TIFF format.<br>2 paraflash and 1 soft box lamp were used<br>Incomplete description of camera	• Low<br>(Daylight regulations were not described.<br>Use of 74-pixel space was not explained.<br>Other 3 shade guides actual name was not provided)	• L and RGB values produced the same coordinate points across all findings.<br>• Daylight images produced significantly different (P<0.01) images when compared to studio images.<br>• Blue and green showed no significant differences (P>.05) while red values were significantly different (P<0.001)	Images were taken and analysed for two different environmental conditions	Adobe Photoshop software can provide reliable colour values the environmental conditions are kept constant

Table S6: Summary of findings for Endodontics and Dental Aesthetics

Endodontics and Dental Aesthetics

Author name (year)	Study design	Aim of study	Measurable outcomes with evaluation	Photographic apparatus used with calibrations	Certainty evaluation with Cochrane GRADEpro	Study outcomes	Novelty of the study	Clinical implications of the study
Almeida,2021 [93]	Comparative Study (In Vivo)	To compare between conventional clinical and indirect digital photographic colour assessment techniques based on the World Dental Federation (FDI) criteria.	• Based on a set of predetermined eligibility criteria, 92 posterior composite restorations were selected.<br>• 2 observers (C1 and C2) scored cases within direct intraoral examinations independently under the same environmental condition.<br>• On the same day, 2 more observers (D1 and D2) photographed posterior restorations for scoring<br>• No images were edited for the correction of colour, brightness and contrast ratio.<br>• Observers used the GIMP-free software (Creative Commons License) to score the images based on the FDI criteria	Single Lens Reflex (SLR) digital camera Sony Alpha a65 (Sony Brazil) with a 100mm Marco lens (Sony Brazil), shutter speed 1/100, aperture F22, ISO 100, a ring flashlight EM-140 DG (Sigma Corporation) was used	• Moderate<br><br>(Incomplete description of environment was provided.<br><br>Inter-observer reliability was not performed)	• Agreement levels between 2 techniques were fair (0.21-0.40) for aesthetic properties and anatomical form and very good (0.81-1.00) for biological properties with no negative values.<br>• The classification varied significantly (P<.05) according to conventional or digital photographic assessments	FDI scoring criteria was used to classify the restoration from 1 to 5	Establishing a predetermined classification criteria can benefit clinical agreement between operators
Garg, 2020 [94]	Experimental study (In vivo)	A management protocol was described for fluorosis with resin infiltration technique which involved photographic and visual colour assessments	• 3 patients with diagnosed fluorosis in maxillary teeth were used as sample<br>• After periodontal scaling, in office bleaching technique was carried out with chemically activated Pola Office Plus (SDI, Victoria).<br>• At the end of the bleaching, Resin infiltration technique was applied with an applicator for 3 minutes and polymerized for 40 seconds with Bluephase C5 light (Ivoclar Vivadent) at 500 mW/cm^2<br>• Photographs of the following instances were taken: Preoperative, post-bleaching, post-resin infiltration and 12-month follow-up<br>• Visual analysis was performed and Adobe Photoshop CS5 (Adobe Inc) was used to calculate the central incisors CIELAB colour values<br>• Colour difference ΔE were calculated by using CIEDE2000 formula	Camera Nikon Coolpix S7000 (Nikon) with a macro lens F/3.4, aperture F3.5, focal length 4mm, no flash and auto white balance correction was used	• Moderate<br><br>(Only the central incisors were evaluated)	• *Subjective evaluation*: the masking of White Spot Lesion (WSL) and Dark spot (DS) by resin infiltration remained stable after 12 months<br>• *Digital Evaluation:* Highly significant differences (P<.001) were found in ΔE values between preoperative and interventional stages.<br>• No significant difference (P=.642) was found in ΔE values between post-operative and 12 months follow-up evaluation	Resin infiltration procedure was used to treat white spot lesion	Digital photography was able to evaluate significant colour changes immediately after treatment, which can serve as an important clinical assessment tool. However, the findings also suggest that the method may not be capable of detecting minute colour variations in the subsequent follow-ups
Kim, 2017 [95]	Cross-sectional study (In Vivo)	To compare the intra-rater and interrater reliability of the FDI criteria using photographic	• Clinical photographs of tooth coloured restoration were searched between 2014 and 2015 databases. 160 photographs were selected and they were cropped to 1500×1500 pixels.<br>• Before one week of colour evaluation, FDI criteria was introduced among 5	Single Lens Reflex (SLR) digital camera D3200 Nikon (Nikon Crop) with a 85mm Macro lens (AF-S DX Nikkor), aperture F3.5 was used	• Low<br><br>(Only the occlusal restorations were evaluated.	• All measurements showed significant differences (P<.001). Pa ranged from 0.41-0.57 and k was 0.09- 0.39.<br>• Highest level of reliability was obtained for marginal adaptation and lowest for surface staining	_	Interpretation of clinical photographs is subjected to large inter-rater variations among clinicians with

		colour matching technique	experienced (2-3 years) raters for training purposes.<br>• The pictures were displayed on a 19-inch computer monitor, resolution 1280×1024 pixels under a fluorescent light room. Raters were asked to score the restoration within 30 seconds.<br>• Scores 1,2 and 3 were marked as clinically acceptable while 4 and 5 were unacceptable.<br>• After one month, 2 raters were asked to score the images again under the same condition in random order.<br>• Interrater reliability was calculated by Proportion of agreement (Pa) and for intrarater, Pa and Cohen standard kappa (k) were evaluated		Only 2 of the 5 raters were conveniently selected for re-evaluation)	• *1 Month follow-up:* For rate 1, Pa and K value were 0.58-0.97 and 0.37-0.95 and for rater 2 Pa and K values were 0.53-0.73 and 0.35-0.58.		2-3 years of work experience
Rauber, 2017 [96]	Comparative study (In Vitro)	To explore the influence of tooth cavity depth on the digital acceptability of restoration colour	• Maxillary typodont with A3 colour was used.<br>• Reference teeth (RT) were prepared by creating a class IV lesion in central incisor.<br>• Lesion was restored using IPS Empress Direct composite resin (Ivoclar Vivadent) which used reproduce the palatal enamel.<br>• DA3 composite resin was applied to recreate the dentine and EA3 was used to create the labial enamel.<br>• Composite resin was light-cured. finishing and polishing was done with Sof-Lex disc (3M ESPE, Seefeld)<br>• 6 test teeth (TT) were prepared according to the following depths: 0.5mm, 0.7mm and 1.0mm.<br>• 3 teeth with class IV cavities were restored with A3.5 dentine resin to simulate moderate discolouration and was labelled as GA3.5-0.5, GA3.5-0.7, GA3.5-1.0.<br>• The remaining 3 teeth were restored with A4 to simulate severe discolouration and was labelled as GA4-0.5, GA4-0.7 and GA4-1.0.<br>• *Visual assessment:* Typodont was photographed with RT and TT. The photographs were printed on 10×10cm glossy photographic paper.<br>• A questionnaire was developed and was distributed among 120 observers: 40 lay people (LP), 40 dental student (DS) and 40 operative dentistry experts (ODE).	Digital camera (SLR) Nikon D60 (Nikon) with a 105mm marco lens (Nikon), shutter speed 1/200, ISO 200 and twin flash (Nikon) f/18 was used	• High<br><br>(The colour of the typodont was conveniently selected)	• 84.72% of 720 evaluations were classified as 'harmonic' (i.e., beautiful) and 15.28% as non-harmonic (i.e., not beautiful).<br>• H perception did not show any significant association (P=.331) but NH showed strong association (P<.001) among the observers.<br>• Harmonic evaluation outcomes: LP=87.5% than DS= 82.9%, and ODE= 83.8%<br>• Visual acceptability was significantly associated (P<.001) to teeth (central incisor) and restoration methods<br>• DA3.5 had the highest acceptance with depths of 0.5mm and 0.7mm.<br>• DA4 had better acceptance for 1.0mm cavities<br>• GA3.5 showed smallest colour difference (ΔE=2.6) while GA4-0.5, GA4-0.7 showed the largest difference (ΔE=8.2, ΔE=7.7)<br>• b* values increased in all groups while lightness values decreased in A4 groups	_	The aesthetic harmony of dental material with tooth surfaces can be judged by lay persons and experts alike when viewing the outcomes of cavity restoration in digital photographs.<br><br>The depth of cavity influenced the acceptability of different shades of restorations

			• Observers scored colour acceptability on a scale of<br>0 – 4. The procedure was carried out inside a standard room with white light (5500K)<br>• VITA Easyshade (Vident, Brea) spectrophotometer was used to calculate the CIELAB and ΔE values for both RT and TT specimens.					
Irawan, 2013 [97]	Comparative study (in vitro)	To digitally evaluate the colour stability of tooth coloured restorative material after bleaching procedure using digital photographs	• 3 groups of samples: Group 1 Filtex Z350 XT nanofilled composite (3M-ESPE), group 2: Estelite S sub-microfilled composite (Tokuyama Dental), and group 3: Ketac N100 (3M-ESPE) nano ionomer cement were fabricated on a plexiglass disc (6×2) mm diameter.<br>• 90 samples were made, and each group was divided again into 3 subgroups (A, B, C).<br>• Subgroup A was bleached with Opalescence home bleaching PF (Ultradent) for 1 hour/day. Subgroup B underwent Chair-side Whitening Boost (Ultradent) and subgroup 3 was treated with distilled water (Control) for 2 weeks<br>• The samples were photographed by using grey card and CIELAB values were calculated using Photoshop CS3 V10.0.<br>• Colour differences ΔE were evaluated for each group with and without bleaching procedure	Single Lens Reflex (SLR) digital camera Nikon D200 with two lights Philips F15TS 15 Watt was used at 45⁰ angle	• Moderate<br><br>(Only A2 shade restorative material was used.<br><br>Incomplete camera description was provided)	• Estellite S showed the highest (3.82±1.6) colour change followed by Ketac N100 (2.97±1.2). Least change was observed in Filtek Z350 XT (2.25 ±10)	_	Digital photographs are capable of differentiating colour changes on both micro-filled and nano-filled restorative materials
Takatsui, 2012 [98]	Comparative Study (In vitro)	To analyse the colour changes that occur while taking photographs using automatic and manual modes of camera	• The following shade tabs were used from Vita Lumin Vacuum (Zahnfabril Vita, Bad Sackingen) with 16 shade tabs: darker colour representing 2 shade tabs (A3.5, C4) and 2 clear colours representing (B1, B3) shade tabs<br>• The Vita shade guide tabs were photographed 5 times on automatic (Aut) and manual (Man) mode individually in front of a grey background. The images were saved as JEPG file.<br>• Adobe Photoshop 7.0 was used to evaluate CIELAB colour values and ΔE was evaluated<br>• *Evaluation of Acceptability:* Determined by using Johnston and kao's classification<br>• *Evaluation of similarity:* 4 shade tabs (Darker and Clear colour) were	Digital camera (SLR) Canon EOS Rebel X Si (Canon) with a macro lens EF 100mm f/2.8, Fluorescent light was used.<br><br>*Manual mode photography*: shutter speed 1/80, aperture diagram 1/16, ISO 100 was used<br><br>*Auto mode photography:* Shutter speed 1/60,	• Moderate<br><br>(Only one camera and brand were evaluated)	• *ΔE values for Automatic mode:* A3.5= 0.89, B1= 1.28, B3= 0.75 and C4= 0.60<br>• *ΔE values for Manual mode:* A3.5= 0.20, B1= 0.45, B3= 1.31 and C4= 1.47<br>• *Classification based on Johnston and Kao*: Only A3.5 presented unacceptable colour differences in both method<br>• A significant colour difference (P<.05) was observed for L* values both in automatic and manual mode.	Acceptability of the colour difference was classified based on the Johnston and Kao classification. Here, a visual threshold limit accompanied CIELAB colour spaces where Δ*E* <3.7 as was judged as clinically acceptable	A single camera evaluation suggested automatic and manual modes to have varying effects on lightness and colour accuracy based on the tooth shade being photographed

			photographed and analysed by 2 examiners.	aperture diagram 1/4, ISO 100 was used				
Athanasios, 2011<br><br>[99]	Comparative study (In vivo)	To evaluate the sensitivity of spectrophotometers and digital photography in accurately evaluating tooth colours	• 14 upper right central incisors of dental students were selected based on the predetermined exclusion and inclusion criteria<br>• The images were calibrated with a professional photographic 18% grey card (Qpcard) and CIELAB colour values were evaluated using Adobe Photoshop.<br>• A reflectance calibrated spectrophotometer (SpectroShade v.2.33) with D65 light source (6500K) at 45⁰ angle was used.<br>• CIELAB and ΔE values were calculated at the start (T0) and after 6 months (T1). A final follow up was conducted 1 week after T1 (T2).	Digital camera (SLR) Fujifilm FinePix S9500 with shutter speed 1/200, aperture F10, a ring flashlight (Sigma E-140 DG) and the ambient light was created by 16 tube lights	• Low<br><br>(Only the colour of the central incisors was evaluated)	• *Error in spectrophotometric evaluation:* L*=1.44, a*=0.43 and b*=0.62<br>• *Error in digital photo analyses:* L*= 0.97, a*= 0.67 and b*=1.25<br>• *Spectrophotometer:* Significant differences (P<.001) were found in all evaluations. Lightness increased by 2.23 while colour intensity decreased by 0.87. b values decreased by 1.16 over 6 months<br>• *Digital photography*: Significant difference (P=.047) was observed among all colour values<br>• No significant difference (P=.496) was observed for ΔE in spectrophotometric analysis but a significant difference (P<.001) was present in digital colour analysis	_	The study suggested calibrated photographs to be less error prone in colour analyses. However, digital photography was shown to be less consistent over long-term follow-ups
Yamanel, 2010 [100]	Comparative Study (In vitro)	To compare the colours obtained from a resin composite shade guide by using colourimeter and digital photography	• 10 shade tabs (A1, A2, A3.5, B1, B2, B3, C2, C3) from 2 nano-hybrid (Grandio, Voco GmbH; Premise, KerrHawa Kulzer) and 2 hybrid (Charisma Heraeus Kulzer, GmbH; Filtek Z250, 3M ESPE) guides were used<br>• The tabs were photographed 3 times from 20 cm distance at 45⁰ angle on a custom-made frame.<br>• Images were transferred into Adobe Photoshop CS2 and CIELAB values were evaluated.<br>• RGB values were transferred to CIELAB colour values with Easy RGB software (Logicol S.r.1, Trieste)<br>• CIELAB colours were re-evaluated using a calibrated digital intraoral colourimeter (shadeEye NCC Dental Chroma Meter, Shofu Inc).<br>• Colour differences (ΔE value) between the two systems were evaluated	Digital camera Fuji S20 Pro (Fujifilm) with shutter speed 1/80, aperture F11, 4 fluorescent tubes: 2 fluorescent tubes (Philips PL-C 18W/865) colour temperature 6500-K on lower socket and 2 fluorescent tube (Philips PL-C 18W/827) on upper socket was used	• Low<br><br>(Shade tabs were conveniently selected)	• L* values: There were significant differences (P<.05) between both colourimetric and digital image analysis method<br>• a* values: Significant differences were observed between digital image analysis (P=.029) and colourimetric method (P=.013).<br>• Except for Charisma (P=.017), no significant difference was observed for a* within the composite groups<br>• b* value: There were no significant differences for both digital photography (P=.487) and colourimeter (.096)<br>• ΔE values among the different composite resin showed significant difference (P<.001) with colourimeter but no significant difference with digital method (P=.099).	_	With certain exceptions, photography is unable to differentiate between the various composite resin shades. Furthermore, the colour values varies significantly from control colourimeter
Kuzekanani, 2009 [101]	Experimental Study (In vivo)	To digitally evaluate the optical changes occurring after application of	• 23 patients with diagnosed tetracycline discolouration were recruited<br>• Pre and post treatment images were photographed	Digital Camera 4500 Nikon Coolpix (Nikon) with 4.5 mega pixel and constant level of illuminated colour-	• Moderate	• A significant (P<.001) lightness effect was observed by the MBPI outcomes for pre and post bleaching treatment	Mean Blue Pixel Index (MBPI) was used to evaluate tooth colour change of tetracycline discoloured teeth which	Computerised evaluations of photographs can be used to monitor colour changes following

		photodynamic laser bleach within tetracycline infused, discoloured tooth	• Flowable composite resin were applied to protect the gum and exposed cervical root surface. Rhodamine-based bleaching gel was prepared and applied to the labial surface<br>• KTP laser treatment (Smart-lite, Deka) was carried out for 30 seconds at 0.8-1.0 Watt, spot size 6mm and delivered by 0.2mm quarts glass optical fibre for per teeth.<br>• The applied gel was removed by vacuum suction and the procedure was repeated for 3 more times. Rhodamine gel was applied for 24 minutes in each instance.<br>• After final cycle the teeth were irradiated for 15 seconds using sodium fluoride gel.<br>• After rinsing off the gel, post treatment photographs were taken alongside VITA B4 and B1 shade tabs as reference.<br>• DOTCAM was used to remove the reflection highlight. Adobe Photoshop (Adobe system Inc.) was used to evaluate the Mean Blue Pixel Intensity (MBPI).<br>• To visually identify cytokeratin in oral mucosa and tetracycline on tooth surface, teeth were irradiated in complete darkness with a mercury vapor lamp and before after laser treatment outcomes were photographed	corrected operating light was used<br><br>During photographs, the windows were blind folded	(Incomplete description of the camera apparatus was provided)	• 78% cases showed a significant (P<.001) increase in MBPI values (116 ±26 to 141 ±27) following laser treatment<br>• At a clinical level, ultraviolet mercury lamp visually confirmed the reduction of tetracycline	underwent laser bleaching	laser-based bleaching therapy in patients undergoing treatment for systemic disorders
Jarad, 2008 [102]	Experimental Study (In Vivo and In vitro)	To evaluate and validate the colour matching ability of digital photographs and CIELAB values in composite restoration	• 9 Munshell colour chip were photographed and RGB values were evaluated and converted to CIEXYZ23 using SigmaScan Pro (Systat Software Inc.). ΔE value were calculated<br>• 3 cavities were prepared on the labial surface of the tooth and restored with composite material by using the shades that had the lowest ΔE values based on digital images.<br>• 10 observers evaluated the colour match, between the composite restorations based on acceptability and best match.<br>• A custom-made shade (CSG) guide was made by using composite material (Spectrum, Dentsply) with 7×3 mm diameter and cured under LED composite light (SmarLite PS, Dentsply).<br>• The prepared discs were photographed next to 10 extracted central incisors placed in a phantom head. 10 photographs were	*Digital camera:* (SLR) Fuji S1 Pro (FujiFilm) with 105mm macro lens (Sigma 105 Ex Macro, Sigma), shutter speed 1/125, aperture F22, ISO 350 was used<br><br>*Repeatability check:* 5 different camera settings:<br>1) Shutter speed 1/120, aperture F22, Flash ¼, distance 30cm, in dark room with tripod<br><br>2) Shutter speed 1/120, aperture F22, Flash ¼,	• Moderate<br><br>(The colour chips, composite material was conveniently selected.<br><br>Only central incisor was used.<br><br>Experts from dentistry were selected as observers)	• There was a significant correlation (r= 0.97, P<0.01) present between CIELAB colour values of Munsell chips calculated from digital image and manufacturer provided colour values of Munsell chips<br>• 6 out of 10 observers rated the composite with the lowest ΔE value to have the best colour match	A custom-made composite shade guide was evaluated	Photograph based colour analysis can yield accuracy comparable to the manufacturer provided data

			taken and CIELAB values were evaluated using SigmaScan Pro software. ΔE values were subsequently evaluated<br>• *For repeatability check:* From CSG, 5 different composite discs (A1, A3.5, B2, B4, C4) were photographed within 5 different camera settings.	distance 20cm, in dark room with tripod.<br><br>3) Shutter speed 1/120, aperture F11, Flash 1/16, distance 30cm, in light room with tripod<br><br>4) Shutter speed 1/120, aperture F22, Flash 1/4, distance 30cm, in light room with tripod<br><br>5) Shutter speed 1/120, aperture F22, Flash 1/4, distance 30cm, in light room with camera handheld				
Lee, 2007 [103]	Experimental Study (In vivo)	To evaluate the effectiveness of 10% and 35% carbamide peroxide bleaching gel by using visual and digital photometric method	• 55 extracted and unrestored molar and premolar teeth were selected.<br>• 5 experienced observers visually compared and scored the pre-bleaching shades with standard tab (Vitapan 3D Master system; Vita Zahnfabrik)<br>• The specimens were then longitudinally sectioned and photographed Infront of a matte metal background with a nut.<br>• The sectioned samples were randomly divided into 2 groups (Group A and Group B) and bleached with the following way: Group A was bleached with 10% carbamide peroxide gel (Opalescence, Ultradent) 8 hours daily for 2 weeks and group B was bleached with 35% carbamide peroxide gel for half an hour only for 5 days 0.2cc of gel was applied to the crown of each specimen.<br>• The specimens were kept moist with damp gauze and kept in an incubator at 37ºC.<br>• After bleaching, visual analysis was done by the 5 observers.<br>• 5 digital images were captured and manipulated in Adobe Photoshop V6.0.	Digital camera 995 ikon Coolpix (Nikon Corporation) was used	• Moderate<br><br>(Camera apparatus details were not sufficiently provided)	• The mean difference between 10% and 35% carbamide solution among the observers was 0.15 which was not statistically significant (P=.846).<br>• Mean significant difference (P<.002) in RGB was observed for both in 10% and 35% carbamide solutions<br>• 35% carbamide solution was most effective (P=.036) for bleaching dark teeth based on the luminosity	A matte metal nut was placed in the pictures to ensure homogenous colour intensity	Photographs can be used to differentiate the strength of bleaching agent used during clinical procedures. This is more pronounced when evaluating teeth of a darker shade

			• Mean luminosity and RGB values were recorded from the pre and post bleaching photographs					
Gadhia, 2006 [104]	Experimental study (In vitro and In vivo)	A custom-made stain shade guide was produced to perform stain analysis procedure	• As staining samples: Perspex blocks and acrylic teeth (Cosmo HXL Shade A1; Dentsply) were used.<br>• The specimens (Set 1-8) were stained in following way: 1st immersed into saliva for 2 minutes, then placed into distilled water for 2 seconds, after which the specimens were immersed into 0.2% chlorhexidine solution for 2 minutes followed by standard tea solution for 60 minutes.<br>• Immersion was carried out in 8 cycles and every cycle; 1 set samples were removed. A set of samples were immersed into only water and labelled as control specimen<br>• A spectrophotometer was used to evaluate the Perpex samples colour values, which was then used to develop a stain guide by staining the acrylic tooth samples with the obtained colour values.<br>• 10 pictures with extrinsic staining were randomly selected and screened on a computer using MGI Photo Suite (V8.1).<br>• Based on the staining intensity, teeth were scored by 2 observers according to "Lobene Stain Index".<br>• Another set of scores were obtained from the custom-made shade guide<br>• *Clinical assessments:*10 volunteers were randomly screened for extrinsic staining and 2 observers visually assessed the patients twice, based on staining area and intensity.<br>• Observers scored the stain by using the Lobene Index and developed stain shade guide	_	• Moderate<br><br>(Acrylic shades were conveniently selected.<br><br>The study relied heavily on staining by caffeine-based tea solution.)	• At the sixth cycle, the maximum optical density (OD= 2.5) and labelled as '3 shade' tab and base optical density (OD=0) was labelled as '0 shade'<br>• Observers showed fair to good agreement (0.38 to 0.79) for Lobene Index and moderate to very good agreement (0.57 to 0.93) for custom made shade guide<br>• Intra-examiner reproducibility was improved by using custom made shade guide compared to Lobene Index<br>• Higher agreement (0.89) was observed among the observers for using custom made shade guide	A custom-made shade guide was developed for stain intensity analysis.<br><br>Lobene Staining index was used as control	Photographic optical density can serve as a reliable digital method of assessing the severity of tooth staining
Lath, 2006 [105]	Comparative study (In vitro)	To compare the suitability of digital image analysis and spectrophotometer for in-vitro stain removal	• *Staining procedure:* 18 acrylic blocks were cut into 30×10×5 mm diameter.<br>• 3 volunteers carried out the staining procedure on the blocks.<br>• The blocks were immersed into the following solutions: saline solution for 2 minutes followed by rinsing with deionized water for another 2 minutes. The blocks were then immersed into chlorhexidine for 2 minutes and again rinsed with deionized water for 2 minutes. Finally, blocks were	Digital camera Kodak Nikon DCS410 (Nikon) 1.5 mega-pixel 32 bit qith a macro lens Elicar single-reflex lens, shutter speed 1/10, aperture F11, 4 fluorescent natural colour tubes Philps 'TL'20 W/05	• High<br><br>(Environment in which photographs were taken was not described)	• Inter-operator reliability was 0.999 for both digital imaging system and spectrophotometer<br>• A strong negative correlation coefficient 0.976 was observed for the two methods in evaluating stain build up and removal<br>• Significant (P= .01) correlation was observed in stain removal for specimen A (0.962), B (0.998) and C (0.817).	_	Digital photographs are capable of clinically monitoring dental stain removal

			immersed into tea (caffeine) solution for 1 hour at room temperature followed by deionized water rinse.<br>• Stains were then measured using Cecil 1000 single beam spectrophotometer (Cecil Instruments Ltd)<br>• *Stain removal procedure:* 15g of two whitening toothpastes (A and B) and 60ml deionized water (Control, C) were used for 1 minute. Procedure was carried out for 5 times<br>• The blocks were photographed using grey background and saved in TIFF format. Adobe photoshop was used to measure L values<br>• *Repeatability and validity check*: 10 measurements were taken from each block.<br>• Absorbance and lightness (L) were measured with spectrophotometer and digital photographs.	(Koninklijke Philips Electronics) was used			
Guan, 2005 [106]	Experimental study (In vitro)	Developed a colour calibration algorithm to digitally evaluate the human teeth colour and validate it while comparing with traditional colour analysis techniques	• A system was developed and calibrated to evaluate the tooth whiteness by brushing and bleaching procedure<br>• 18 extracted teeth were stored in phosphate-buffered saline solution containing thymol.<br>• Each tooth was brushed for 2 minutes using an electronic toothbrush (Oral B Plaque Remover) and toothpaste (Boots Freshmint Fluoride Toothpaste)<br>• *bleaching technique:* the teeth were placed within a freshly prepared 43% urea hydrogen peroxide solution (Sigma, U-1753) which was equivalent to 15% hydrogen peroxide (H_2O_2)<br>• An algorithm was developed to evaluate the colour change measurements for each treatment using both digital photographs and spectrophotometer (Minolta CM2600d)	Digital camera Kodak Nikon DCS410 with shutter speed 1/10, aperture F11, 2 natural colour fluorescent tubes (Philips, TL'D 20W/05) and 2 ultraviolet fluorescent tubes (Phlips, TL 20W/05) was used	• Moderate<br><br>(Brushing technique variations were not considered.<br><br>Camera temperature settings were not mentioned.)	• 2 min brushing with toothpaste had little effect on tooth whitening for both image (P<.31) and spectrophotometric analysis (P<.22)<br>• Bleaching with 43% hydrogen peroxide showed a significant (P<.05) whitening effect with CIE image analysis both for the spectrophotometer and digital image.<br>• CIE whiteness index was more marked in digital photography than spectrophotometer	An algorithm was developed to evaluate the whiteness parameters	Photographic colour evaluations may be less effective in determining minute whiteness changes brought upon by daily brushing practices, but can be effective in observing chemical based bleaching services
Tsuzuki, 2002 [107]	Comparative Study (In vivo and In vitro)	To evaluate the effectiveness of intra-oral CCD (Charge-Couple Device) camera for forensic examination of	• Jaw opening on a dry skull was fixed (20mm anteriorly and 10mm posteriorly) to simulate the situation of difficult mouth opening on a cadaver.<br>• Commercially available CCD camera, Cristal Cam (GC Crop) with 250,000 pixels was used to examined and photographed the teeth.	Single Lens Reflex (SLR) digital camera NikonF2 (Nippon Kogaku K.K) with a 120mm Nikon macro lens (Medical-Nikkor, Nippon Kogaku K.K) was used	• Low	• It was possible to easily differentiate gold and silver colour metal restoration using CCD derived images<br>• Odontologists faced difficulty in distinguishing between carious lesions, discolouration and aesthetic restoration	CCD cameras were used in forensic odontology	CCD and digital cameras were shown capable of identifying features in forensic cases. However, the cameras were unable to distinguish between

		caries and restoration	• Digital File Printer, SP-X1 (Scalar Crop) was used for image construction and edited using Adobe Photoshop V5.0 (Adobe System Inc)<br>• The skull was again photographed using a digital camera for establishing a comparison with CCD camera<br>• *Clinical assessment:* A female subject was instructed to bite on a 20mm block and the teeth were photographed using both methods.<br>• The buccal and occlusal views were transferred to 3 forensic odontologists and asked to construct a dental chart					caries and discolouration
Bentley, 1999 [108]	Cross-sectional study (In vivo, In-vitro)	To analyse the ability of photographic and computerised techniques in recording the colour changes brought upon during nightguard vital bleaching (NGVB) procedure	• 3 photos of subject's maxillary teeth were captured at base line, 7 days and 14 days after NGVB with 10% Carbamide Peroxide (CP).<br>• B1 and C4 shade tabs were considered internal standard for calibration<br>• For reproducibility check 2 shade guides (Vita Lumin, Vident), were photographed 6 times in front of white and green backgrounds and digitised to RGB PICT images using a scanner (Microtek 35t)<br>• Image processing and reflection removal were done on Adobe Photoshop software.<br>• The RGB and CIELAB values were extracted from the resultant histograms	Camera (Dental Eye-II, Yashica) with 100nm f/4 lens, 35 mm film (Kodachrome 64, Eastman Kodak Co.) and a electronic flash illuminator ring-light was used	• Low<br><br>(Participants were conveniently selected.<br><br>Incomplete description of the environment was provided.)	• Both clinical evaluation and computerised analysis were able to distinguish the lightness change after 7 days of whitening.<br>• D3 shade tab-included photographs showed excellent reproducibility (0.5 ±.03)<br>• RGB and CIELAB values in the single shade guide photographs showed that the blue channel had the best correlation (r=.77) with mean pixel intensity (MPI)<br>• There was a highly significant MPI difference (P= 9.1×10^{-11}) when different backgrounds and different time periods were evaluated	Photographic technique was used to evaluate the colour	Photographic image processing can allow for clinical monitoring of bleaching outcomes. The procedure is however sensitive to the environment under which the photographs are taken as well as the time difference from when treatment commenced

Table S7: Summary of findings for Prosthodontics

Prosthodontics

Author name (year)	Study design	Aim of study	Measurable outcomes with evaluation	Photographic apparatus used with calibrations	Certainty evaluation with Cochrane GRADEpro	Study outcomes	Novelty of the study	Clinical implications of the study
Yousof, 2020 [109]	Cohort study (In Vivo)	To evaluate validity of colour mixing analysis method based on masticatory performance	• 20 volunteers (10 natural dentition and 10 denture wearers) were selected<br>• Each subject was asked to chew coloured (Red- green) wafer within 1-25 masticatory cycles. The cycle was repeated 15 times with 1-minute rest interval.<br>• A 1-mm thick wafer was made and scanned using flatbed scanner (CanoScan 4400F, Canon).<br>• 600 images were analysed and merged using an image-analysis programme (ImageJ 1.51 m, US national institute of health).<br>• CIELAB colour space were used to detect L*, a*, b* values. Segmentation of the images were done and segmentation perimeter, area, region of interest (ROI) were measured<br>• Masticatory index was analysed using Mastimeter plug-in.<br>• Hue-Saturation-Intensity were calculated using ViewGum 1.4 software	-	• Low<br><br>(The proposed method was not compared to any gold standard)	• Increased masticatory cycles produced elevated geometric depression with reduced a* value<br>• Mastimeter produced significantly better (P<.001) mastication analyses than ViewGum software.<br>• Complete denture wearers demonstrated 55% less mastication ability in the evaluations.	Masticatory tendency of prosthetics was evaluated using open-source software	Open-source digital colour analyses can be used to test prosthetic masticatory properties
Sam, 2019 [110]	Compara tive study (In Vitro)	To assess the difference in colours between shade tab and glazed porcelain	• 40 glazed metal ceramic porcelain were compared against VITA classic shade guide and photographed<br>• Image Colour Summarizer 0.76 software was used to analyse CIELAB colour cluster values	Canon rebel t6i camera with 28-135mm macrolens under natural lighting condition with ISO-6400, aperture f/22 and shutter speed 1/200.	• Low<br><br>(Glazed material photographed under natural light could introduce possible reflection-based errors during photo capture.<br><br>The results could have been influenced by the porcelain thickness and metal properties)	• a* produced significant differences (P<0.01) while L* (P=0.059) and b* differences (P=0.677) were insignificant.	Prosthetic photographs were divided into image clusters prior to CIELAB analyses	Photographs can aid colour accurate crown restorations that contrast gingiva, lip and patient’s facial features
Sadek, 2018 [111]	Compara tive study	To evaluate staining properties of prosthetic clasps	• 10 in-vitro specimens were made and equally distributed for immersion into coffee solution and distilled water.	Incomplete description of camera was given	• Low<br><br>(As the study evaluated the colour of the clasp	• Acetal clasps were found to be most colour stable in photographic comparisons	Digital photography was used to test the colour stability of prosthetic	Digital photographs and virtual colours can detect prosthetic

		when subjected to caffeine.	• Photographs were taken at 3-day intervals for 4 weeks<br>• A calibrated digital camera with 8 LED lights and image processing software (Image J 1.43U) were used to register the colour values.		from the exthetic point of view but the whole procedure was carried out upon a study model, no human sample was added)	• Cobalt Chromimum metal clasps showed poorest aesthetics in photographs	clasps during materials testing	colour changes influenced by caffeine.<br><br>Acetal clasps were photographically most colour degradation resistant.
Kim, 2016 [112]	Comparative study (In vivo)	To evaluate fluorescence intensities of different composite resin restoration using Quantitative Light-induced Fluorescence (QLF) photography.	• Image analysis software (Image J,1.47) converted the RGB values of of the photographs into 8-bit greyscale image. And tooth fluorescence images were converted to monochrome image.<br>• Greyscale evaluations were made accordingly	All photographs were taken using digital camera with QLF lens (QLF-D BiluminatorTM 2+, Inspektor Research Systems)<br><br>For white-light: 250 ISO, shutter speed of 1/13s and 14.0 aperture were used.<br><br>For fluorescent images, ISO 1600, shutter speed of 1/15 s, aperture value of 13.0 were used	• Low<br><br>(Tooth samples were conveniently selected.<br><br>The blue lights present within the study contains blue lights may not be suitable for inducing highest fluorescent intensities.<br><br>The filter on QLF-D technology sometimes filters out extra wavelength resulting in greater fluorescence intensity)	• All six composite resins showed different in fluorescence intensities	Digital camera was used to record the fluorescence emitted from tooth-restoration complex under a visible light source	Digital cameras can be used to test material fluorescence in clinically relevant simulations
Lakhanpal, 2016 [113]	Comparative study (In Vitro)	To assess the shade matching ability of three different devices for metal ceramic crown restoration	• 20 extracted premolars were used as sample. A spectrophotometer was used to obtain CIELAB values from the middle third of the tooth and photographed in a dark room<br>• 40 images were taken with and without polarizer. MATLAB software was used to extract the CIELAB values. A 4 mm area was cropped to standardize the measurement area	• Single-lens Reflex (SLR) digital camera Canon EOS 1100D camera with 18-55 macrolens, a ring diode flash-light (Meike FC100) and two polarizers (Hoya 52mm) were used for image capture.	• Low<br><br>(Only premolars were evaluated for the study)	• L* and a* values produced significant differences in spectrophotometer, digital camera and polarizers (P<.001)<br>• No significant difference was observed for b* values among all the groups (P>.05) for both method	Polarisation dental imaging modality and image analysis was used to eliminate light reflections on the tooth surface	Photography can produce decreased edge-loss and acceptable ΔE values when salivary reflections are controlled
Bilt, 2012 [114]	Cohort study (In vivo)	To evaluate prosthetic masticatory tendency using photography and virtual colour analyses	• 60 participants with dentures were made to chew coloured wax in levels of 5-20 strokes<br>• After chewing, the wax pattern was scanned and processed using Adobe Photoshop, CS3<br>• The images were exported as 8-bit histograms to analyse the mixing index	-	• Moderate<br><br>(The study did not include the spatial information of the wax which may have influenced different frequencies of light intensities to be present)	• Digital image analysis was able to differentiate all levels of chewing strokes while human observers were able to differentiate them only after 20 strokes	Masticatory tendency of prosthetics was evaluated using virtual colour differentiation and visual analysis	Digital colour analyses can be used to test prosthetic masticatory properties

			• Human observers were asked to rate the wax as control for comparison to the digital colour analyses					
Vafaii, 2012 [115]	Comparative study (In Vivo)	To compare the accuracy of colour matching and defect contour detection between direct vision and digital photographs	• A patient requiring porcelain fuse to metal crown (PFM) was selected<br>• A prosthodontist selected the shade for the crown using 3D master shade guide.<br>• T3K (Ticonium) and Vita VMK95 (Vident) were used to construct 10 PFM shells with intentional defects<br>• 20 observers with healthy eye vision (Ishihara test) evaluated the samples, stain and contour deficiencies by means of shade guides<br>• All the samples were photographed and printed on light sensitive papers (Noritso).<br>• After 2-3 weeks of direct visualisation, contour defect and need for stain were evaluated from photographs<br>• Colour difference between the two shade selection methods were evaluated	Digital camera Canon S2 IS (Canon) with light source.	• Very Low<br><br>(Incomplete camera details were provided.<br><br>The lighting apparatus was not clearly defined.<br><br>The observers had varying experience in restorative dentistry.<br><br>The parameters used as standard were unclear)	• Colour difference scores were significantly lower in direct vison (ΔE=7.41 ±13.37) than digital images (ΔE= 11.14 ±13.03)<br>• There was 18.2% agreement in direct method and 7.2% in digital image method<br>• For contour defect detection, both methods showed significant differences (P<1%) in buccolingual surface, inciso-gingival height and error in line angle. However, there was no significant difference (P>1%) in incisal and occlusal edges	_	Detection of surface defects are easier done using colours obtained from photographs
Won-suk Oh, 2010 [116]	Comparative study (In Vitro)	To validate the use of digital Photo Colourimetric Method (PCM) for tooth colour matching in comparison with visual analysis	• 2 Vitapan Classical Shade tabs (Vita Zahnfabrik) A and B were arranged from lightest to darker shades according to the manufacturer's instructions and pair matched.<br>• The image of the 3 paired groups were photographed within a colour matching booth (GTI ColourMatcher, GTI) using D65 daylight in front of a white background<br>• Eye-One instrument (Eye-One Match, Gretag-Macbeth) was used to measure the CIELAB Values. First the values of the A group were measured to serve as reference. Then the values of Group B were evaluated. In the meantime, the software automatically calculated the colour difference ΔE<br>• For visual analysis, 2 prosthodontists (Observer A and B) independently selected 3 shade tabs from Vitapan Classical Shade guide and ranked them based on the patient's target tooth.<br>• 48 participants were photographed (anterior teeth) alongside the selected shade tabs	Single-lens Reflex (SLR) digital camera Nikon D70s with a 105mm Macro lens, shutter speed 1/125, aperture F20, bilateral flash (R1 TTL Ring Light Flash, Nikon) at 45º angel was used	• Moderate<br><br>(The shade tabs were conveniently selected.<br><br>Only the colour values of the anterior teeth were evaluated)	• The ΔE value between the duplicate shade guide tooth was 2.3 which was an 88% correct match<br>• A1 and C1 were incorrectly matched with B1 and D2<br>• There is no significant difference (P=.05) between the correct shade matching of duplicate shade guide and human teeth.<br>• Observers agreed (75%) to using PCM methods for human teeth	_	Photographed methods of colour matching have varying degrees of inaccuracies. However, specialists agree to their applicability for human tooth colour matching

			• ΔE values between the best matching tabs with the target tooth and the PCM method on a digital image were evaluated				
Cal, 2007 [117]	Comparative study (In vitro)	To evaluate staining ability of different mouth rinse solutions by applying digital colour analysis	• Chlorhexidine gluconate, benzyde-amine hydrochloride, benzydamine hydrochloride (hybrid) and distilled water were used with coloured acrylic resin.<br>• A digital camera was used to take images of the sample and later on software Adobe Photoshop 6.0 was used to evaluate L*, a*, b* values before and after 12 hours of immersion.	Incomplete description of camera was given	• High<br><br>(Shade and colour of the acrylic resins were selected randomly)	• In general, all mouth rinse solutions discoloured lighter shades of acrylic resin (ΔE=5.5) more than the darker shades (ΔE=2.9)<br>• In lighter shades, chlorhexidine gluconate mouth rinse caused more colour change (ΔE=5.7) than benzyde-amine hydrochloride (ΔE=4.9)<br>• Whereas, in case of darker shades, benzyde-amine hydrochloride (ΔE=2.9) caused more colour change than chlorhexidine gluconate mouth rinse (ΔE=2.6)<br>• Hybrid mouth rinse caused highest colour change on both lighter (ΔE=7.3) and darker shades (ΔE=4.1)	Digital photography was used to test the colour stability of acrylic resin during materials testing	Digital photographs and virtual colours can detect prosthetic colour changes influenced by mouth rinse
Wozniak, 1981 [118]	Comparative study (In vitro)	To evaluate colour changes of two commercially available acrylic resins by using photographic technique	• Acrylic resin disks were immersed in caffeine of varying temperatures<br>• IDL Colour Eye colourimeter were used against Vitrolite tile to obtain colourimetric values which were converted to CIELAB using PDP-12 computer. The values were also converted into Munsell Hue and chroma value<br>• Photographs were taken in 0, 5, 10, and 15 days of immersion period using Calumet view camera with a grey card<br>• Macbeth Transmission Densitometer was used to measure optical density.	Incomplete description of camera was given.<br><br>4 types of filters, No. 94 (420-490 nm), No. 93 (510-590 nm). No. 92 (620-700 nm) and No. 106 (420-700 nm) were evaluated for the photographs	• Low<br><br>(Temperature control or variations were not specified)	• For all staining solutions, cold cure acrylic resins showed higher ΔE values than heat cured acrylic resins.<br>• Tea produced the largest ΔE values<br>• Coffee produced the greatest optical density change in acrylic.<br>• Filter no. 93 (510-590 nm) showed the best result for colour change detection	One of the first recorded studies of optical density calculation and colour degradation evaluation using camera photographs	Digital colour analysis techniques in dental material research have been simplified substantially over the last few decades.<br><br>Cold cure acrylic showed greatest staining in cameras due to greater porosities
Mccarty, 1976 [119]	Case series (In Vivo)	To visually compare the usefulness of infrared and Ektachrome intraoral photography as a diagnostic aid	• 6 patients undergoing radio therapy treatment were evaluated for oral mucosa changes.<br>• The patients' buccal regions, hard and soft palates were photographed weekly using Infrared and Ektachrome film<br>• As manufacturer instructions, the infrared photographs were made using yellow filter. Both with and without filter photographs were taken<br>• A pair of projectors (Kodak Carousel, Kodak) were used to visually compare	Nikon camera 2x tele-extender with 50mm lens and a ring flashlight was used.<br><br>*Infrared film:* shutter speed was 1/60 and aperture F16.5 was used<br><br>*Ektachrome film:* aperture F22 was used	• Moderate<br><br>(Inadequate camera apparatus was provided)	• Both Infrared and Ektachrome films could not differentiate between irradiated and nonirradiated oral mucosa.	Infrared films were used to photograph irradiated oral tissue	Infrared films were not capable of differentiating oral irradiated zones from healthy tissue

between the photographs obtained from infrared and Ektachrome slides.

Table S8: Summary of findings for Maxillofacial Prostheses

Maxillofacial Prostheses

Author name (year)	Study design	Aim of study	Measurable outcomes with evaluation	Photographic apparatus used with calibrations	Certainty evaluation with Cochrane GRADEpro	Study outcomes	Novelty of the study	Clinical implications of the study
Mulcare, 2019 [120]	Cohort study (In vitro)	Suitability of a mobile phone colourimeter for shade matching during fabrication of silicone prostheses.	• 'e-skin' Spectrocolourimeter (Spectromatch) was used as control. A smartphone was placed at distance of 25, 30 and 35 mm using the jig.<br>• Evaluation of trueness and accuracy of software-based colour detection in comparison to standard spectrophotometric values.	Mobile phone camera (iphone 5s) with D65 fluorescent illumination in a Munsell 8 grey light box was used	• Moderate<br><br>(An unclear description of the custom-made jig was given.<br><br>The smartphone, silicone material and pigments used were conveniently selected)	• White background is more accurate than the black background.<br>• For obtaining a true colour measurement, the mobile phone should be placed more than 25 mm away from the prosthesis	Used a smartphone-based colourimeter software and a custom-made calibration jig to standardise the experiment	The clinician can use their existing smartphones during colour matching of the prosthetic silicone elastomer
Lagouvardo, 2018 [121]	Cross-sectional study (In vitro)	Perceptibility and acceptability of facial skin colour difference as seen on digital monitors	• 81 digital skin colour photos (4.9 × 6.0cm) were created and randomly shown to the observers on a 21.5' 1080p colour monitor screen from a distance 57-60 cm.<br>• Visual analysis to evaluate perceptibility and acceptability	_	• Moderate<br><br>(Different age groups and genders have varying tolerance to colour changes. Such changes were not controlled in the study)	• Individual Δa^* and Δb^* values of up to 2.0 and 2.5 units was deemed acceptable by the observers.<br>• ΔE values of more than 1.8 was not acceptable.<br>• Male participants are more sensitive to Δb^* than females	Computer generated prosthetic skin colours were used in the evaluation	subjective acceptability should be obtained from the patient prior to supplying the prosthesis
Sohaib, 2018 [122]	Experimental study (In vivo)	To optimise the colour profile accuracy during additive manufacturing of facial prosthesis	• 3D photogrammetry was used to scan the prostheses and convert to 3D models<br>• Following fluorescent illumination lamps: CIE daylight D65 and D50, illuminant A and three types of CIE fluorescent illuminants (F2, F7, F11).<br>• Medical grade silicon powder (ZP 15E) was used in the 3D printer (Spectrum Z510) for manufacture of the prosthesis.<br>• For optimisation ColourChecker Digital SG chart was 3D printed and values for each colour patch was noted using CM700d spectrophotometer<br>• PANTONE skin shade guide to was used to validate profile optimisation.<br>• The quality of the colour reproduction in 3D facial prosthesis was assessed by using a perceptual error formula such as CIEDE2000 and spectral analysis using RMSE and SSV.	3dMD imaging system with camera-built flash-light, colour temperature 5500k and D65 illuminate was used	• Low<br><br>(Maximum constancy depends on the choice of illumination.<br><br>A limited number of lighting conditions were evaluated in the current study.<br><br>Skin samples were only chosen from Caucasian and Chinese subjects)	• 3D TPS method produce more accurate colours than 3rd order polynominal regression when 3D printing facial prostheses<br>• There were errors in both colourimetric and spectral assessments for optimisation<br>• Author stated that under extreme external conditions the measurement would be representative as there would be different physiological and physical conditions embedded to influence facial colour accuracy	A colour consistency index was rapid prototyped to evaluate prosthetic colour difference in different light illumination.	The accuracy of facial skin colour is greatly dependent on individual emotions and the type of hardware used to collect the data

Pricop,<br>2016 [123]	Case report (In vivo)	Use of photographic images in the manufacturing of iris for ocular prostheses	• Healthy eye image was captured, and a photo editing software was used to remove the light reflection and for colour correction. The iris was then printed onto photographic papers<br>• Cyanoacrylate adhesive was used to attach the printed iris to the prosthesis<br>• The printed iris technique was compared with the conventional technique	Crop sensor DSLR camera with 100mm micro lens, natural light and a grey card was used	• Low<br>(The study did not consider operator's experience in conventionally painting the prosthesis<br>Incomplete camera details were provided)	• 100µm laminating pouch can prevent colour distortion during printing the iris.<br>• Additional polymerisation resulted in colour distortion of the printed iris.	Photographed images were printed instead of conventionally painting the iris onto the prosthesis	The appearance of printed iris is comparable to the healthy contralateral eye
Grant,<br>2015 [124]	Case report (In vivo)	Applied digital technologies to make a nasal prosthesis for a 4-year-old child	• Stereophotogrammetry was used to capture full face images of patient and donor<br>• Donor nasal and mid-face soft tissue geometry were isolated using (Magics; Materialise)<br>• Mould was designed using binder-jetting additive manufacturing technique (ProJet 460 plus; 3D Systems), with cyanoacrylate resin (Apollo 5005 Cyanoacrylate), and sealed with 2 coats of clear acrylic resin<br>• After that conventional method was carried out make the nasal prosthesis<br>• Article compared between conventional procedure and digital procedure	3dMD cranial system stereophotogrammetry; 3dMD	• Very low<br>(Shade mixing relied predominantly on conventional visual perception)	• The 3D image was shown to be modular, could be resized and reshaped to make another prosthesis for the same patient during growth	The model nose template for a child patient was obtained from another donor	Digital workflow greatly reduces the stress and anxiety in paediatric patients<br>The process allows for repeated prosthetic manufacturing with minimal efforts
Buzayan,<br>2015 [125]	Case report (In vivo)	Use digital photographic image for manufacturing iris for ocular prosthesis	• Healthy iris image was captured<br>• Graphic software (Paint Shop Pro X4 version 14.0.0.322) was used to adjust colour, brightness, contrast, and hue<br>• The printed iris disc was fixed with scleral blank by using cyanoacrylate adhesive<br>• Printed iris was compared with the conventional iris painting procedure	Digital camera (Canon EOS Digital Rebel) was used	• Low<br>(Incomplete camera details were provided.<br>Duplication of the iris depends upon quality of the printer ink, printer paper type and skills to use the software including digital photography technique.)	• Anterior curvature of the scleral blank was preserved which provide less adjustments of try-in at the insertion visit.	A printed photographic disc was directly pasted onto acrylic scleral blank.	The proposed technique is less time consuming and required less artistic skills than conventional painting technique
Xiao,<br>2014 [126]	Experimental study (In vitro)	To 3D print maxillofacial prostheses with accurate skin colour	• Photogrammetry was used for data capture and conversion to monochrome model<br>• Camera RGB were transformed to CIELAB values by Matlab and Materialise 3-matic software was used for texture mapping<br>• A Z Corp Z510 (3D Systems Inc.) 3D colour printer was used<br>• Minolta CM-2600d spectrophotometer was used for colour measurement	3dMD photogrammetry facial system (3dMD, Atlanta)	• Low<br>(Only two prostheses; one nose and one ear were validated with this proposed technique)	• The colour difference was approximately 3-4 ΔE^*_{ab} for the 3D printed soft tissue prosthesis	A custom colour profile was developed for 3D printing.<br>The accuracy was validated by 3D printing facial prostheses using starch and colour 3D printer	A 3D colour chart can aid in more accurate prosthesis printing when used with a colour 3D printer

			• Author printed a 3D colour chart which was then compared with the original colour chart reference values				
Tan, 2013 [127]	Case-control study (In vivo)	To evaluate participants' colour detection thresholds to colour changes in Asian, African, Caucasian facial skin and computer-generated colour patches.	• a Gretag Macbeth Mini ColourChecker was added to the frame of the photograph for colour calibration. The photographs were digitally stored<br>• To produce the patches with even skin colouration, Matlab was used to recreate the colour spaces and Gaussian blur was added to the edges. Colour patches were made based on the ethnicity's groups within the study<br>• Images were shown to the participants as digital images on a 4:5 aspect ratio computer screen<br>• Evaluate any differences in sensitivity among participants when viewing human face skin versus computer generated colour patches.	Munsell N5 grey and illuminated with 3 Verivide F20 T 12/ D65 daylight bulbs were used	• High<br>(Only one Asian population was included as participants.<br>The participants were selected randomly and accepted all age ranges)	• The participants found cross-ethnic facial colour variation difficult to detect.<br>• Differences in computer generated colour patches were harder to detect than facial photographs	The skin samples were captured by photographs. The images were used to software calibrate and create similar digital skin colour patches.	Facial skin colour show variation in different physical health, emotional and person's ability to differentiate colour change and must be kept in consideration when designing prostheses based on photographic colours
Xiao, 2013 [128]	Experimental study (In vitro)	This study developed a colour reproduction profile for accurate and automatic processing of soft tissue in advanced manufacture technology.	• A 2D model was 3D printed and evaluated for variations using Minolta CM-2600d spectrophotometer<br>• A 240 Macbeth colour chart RGB values was used to map the colours virtually as LAB using 3Matic and VRML 2.0 visualisation package<br>• A profile was generated which was 3D printed (ZCrop Z510) and evaluated<br>• CIELAB values, chroma and hue were measured to evaluate colour consistency upon repeated production	_	• Low<br>(Selected skin shades were chosen for evaluation of reliability and repeatability)	• Less than 3.0 units of colour difference was acceptable for making the soft tissue prosthesis<br>• Extremely dark or bright skin colour produced inaccurate values<br>• The system struggled to maintain reliable colour outputs	A colour profile was developed to directly link human colour perception to software-based colour using a mathematical model based on 240 training colours.	A 3D colour chart can aid in more accurate prosthesis printing when used with a colour 3D printer
Seo, 2011 [129]	Cohort study (In vitro)	To evaluate the effects of light filters and white balance on digital camera images within dental practice	• 2 MacBeth colour charts (Gretag MacBeth and QPcard 201) were photographed<br>• Tungsten lamp (Eye Reflector Photo Lamp, 500W, 230 V, base E27; Iwasaki Electric Co. Ltd.,) and four colour temperature filters (200 double CTB, 201 full CTB, 202 1/2 CTB, and 203 1/4 CTB;) were used<br>• CIELAB colour values obtained by photographs were compared between different colour filters	Single-lens Reflex (SLR) digital camera (EOS-350D; Canon Inc.) with a macro lens (SP AF 90 mm F/2.8 Di MACRO 1:1) were used	• Moderate<br>(The study design cannot be reproduced for dental prostheses)	• Tungsten lights with colour temperature conversion filters produced daylight-like light, and resulted in clinically accurate photographs<br>• Custom white balance with grey cards introduce complexity in clinical photography when used in different clinics	The study used 4 different colour temperature filters and 2 different colour charts	Colour filters are necessary to obtain clinically accurate images
Delallea, 2011[130]	Experimental study (In vivo)	Modification of the existing techniques of colour detection on skin tissue to	• Calibration was made from device dependent RGB to a standard device-independent sRGB. MacBeth ColourChecker was used for linear equation	Video recorder with an incorporated white LED light source, resolution (2048 × 1536), frame rate 20 fps was used	• Very low<br>(For proper shading correction is based on adequate lighting	• The ΔE values were <3.0 and deemed acceptable by the standards of human perceptibility<br>• The calibration may be greatly affected by high and low intensity light sources	A new light modelling technique was proposed to improve shade correction of skin texture	The technique can prove beneficial in follow up evaluations and in order to detect recurring neoplasm such as melanoma

		allow for more accurate colours and reduced noise.	• Shading correction was done by acquiring a white patch image combined with sRGB planes and polynominal regression.<br>• Camera accuracy was tested by using a Chromameter CR400 Konica Minolta as a reference device<br>• The modification was compared against the previous techniques by camera-based images of 10 skin patches		condition and standardisation.<br><br>The current study made no mention of light control)			
Jain,<br>2010 [131]	Case report (In vivo)	Used digital photographic technique for fabrication of an iris for ocular prosthesis	• Healthy iris image was captured<br>• Graphic software (Paint Shop Pro X4 version 14.0.0.322) was used to adjust colour, brightness, contrast and hue<br>• Photoshop 7.0; Adobe system Inc. was used to adjust colour, brightness, contrast and hue<br>• The printed iris disc was fixed with scleral blank by using cyanoacrylate adhesive<br>• Printed iris was compared with the conventional iris painting procedure	Digital camera (Nikon COOLPIX P90), 12.1megapixel, 24× optical zoom was used	• Moderate<br><br>(Special digital photography equipment and setting were needed for image adjustment which will be a limitation for making iris digitally)	• Common technique for fabrication of an eye prosthesis were paper iris disk technique and black iris disk technique	A printed photographic iris disc was directly pasted to acrylic scleral blank	Digital images provide an aesthetically pleasing replicant iris in a less time. and requires less colour adjustment
Pladellorens,<br>2008 [132]	Experimental (In Vivo)	To develop and evaluate a system to detect skin colour variations	• A grey card was used to control pixel sensitivity and non-uniform illumination<br>• The skin colour was first analysed using CIELAB coordinates with individual ITA parameters. Then, system stretched histogram equalisation was used for spot detection. Finally, processing was done by rhomboid erosion followed by square structural element dilation<br>• Spectrophotometer MINOLTA CM-2002 (Minolta Camera Co.) was used for colour measurements<br>• 30 measurements were taken from different samples and values of the device were compared against spectrophotometric values	An RGB CCD camera with an 8-bit depth (AVT Marlin F033C, AVT GmbH) was used	• Very low<br><br>(The samples were not properly defined.<br><br>The device accuracy was limited to an area of between 1 and 300mm^2 due to lack of illuminance of the edges.)	• The device scored slightly higher than the control.<br>• Spot detection was effective either as area of dark pixels in mm^2 or identifying red pixel squares of certain values within the skin shade.	A device and associated algorithm was proposed to evaluate skin colour and facial spots	The proposed technique can aid in superior cosmetic accuracy and aid in both facial prosthetic and dermatological clinical management
Tao,<br>2003 [133]	Experimental study (In vivo)	Developed and implemented a colour system for detection of human face within the colour image.	• 250 samples were taken, and the cluster of chromatic colour space was presented by a Gaussian model *N (m, C)*. A chroma chart was obtained from grey scale image.<br>• The samples were matched and cross-correlation value between template and skin was used to isolate facial skin from environment<br>• The algorithm was tested against other face detection methods	_	• Very low<br><br>(The ethnic groups were conveniently selected for the study)	• Real time face detection speed was 0.8 second/image<br>• The threshold value for classifying a region as human face required a cross-correlation value of greater than 0.6<br>• The algorithm was comparatively less accurate than other methods of face detection	An algorithm to detect facial colour in real time	Real time facial skin colour detection methods are reliable but with varying levels of accuracy.<br><br>RGB colour space is not reliable for accurate facial skin tone identification.

Table S9: Summary of findings for Periodontics and Oral Pathology

Periodontics and Oral Pathology

Author name (year)	Study design	Aim of study	Evaluation methods and measurable outcomes	Photographic apparatus used with calibrations	Certainty evaluation with Cochrane GRADEpro	Study outcomes	Novelty of the study	Clinical implications of the study
Ghanoum, 2019 [134]	Experimental study (In vitro)	To create a 3D stereo photogrammetric panoramic model using an intraoral camera	• An intra-oral camera was used to capture the image of teeth and gum on an STL with light modification done in Blender software.<br>• Shape from Shading (SFS) algorithm was used to obtain matric representation from the teeth and gum<br>• To correct the surface 'normal', a cross product was applied between the normal surface and light direction vector.<br>• Mean filtering was performed using window size 9 and the image was converted to grey scale<br>• KAZE algorithm was used to extract normal features.<br>• Image stitching was done by flexible warps with location dependent homograph.<br>• The resultant models were compared using structural similarity index	The details of the intra oral camera was not provided	• Low<br><br>(SFS cannot always provide accurate information on the surface normal.<br><br>The study relied on software-based simulations of intraoral lighting conditions)	• The generated images were very similar to ground truth (0.820-0.885).	Intraoral camera photographs were used to create digital models	Intraoral camera images can be stitched to produce 3D models provided light reflections and abnormal oral cavity contours are controlled
Mayer, 2017 [135]	Cohort study (In vivo)	To use digital photographs to monitor gingival condition changes following periodontal therapy	• 53 Caucasian patients with periodontal diseases were selected<br>• Maxillary anterior segments were photographed before and after 3.5 months of periodontal treatment.<br>• Maxillary lateral incisor's buccal attachment was selected to evaluate the colour<br>• Digital colour analysis was performed using CIELAB colour space by using a software	Single-lens reflex (SLR) digital camera with 60mm macro lens, shutter speed 1/125s and aperture F20	• Low<br><br>(A single tooth was analysed which might not justly reflect the rest of the dentition.<br><br>Only one race of samples was selected.<br><br>White balance and colour calibration methods were not mentioned.<br><br>Software used for colour evaluation was not mentioned)	• Δa^* values were within 3.37 indicating a reduction in inflammation (redness)<br>• ΔE values for all the patients were within the accepted threshold 3.7	Gingival index was evaluated from photographs based on gingival colour	Photographs can accurately index gingival inflammation

Berdouse, 2015 [136]	Experimental study (Both in vitro and in vivo)	To assess carious lesion on the occlusal tooth surface using coloured photographs in machine learning	• 91 posterior extracted teeth and 12 in-vivo teeth were selected and photographed<br>• The image was analysed and scored by two observers using International Caries Detection and Assessment System (ICDAS II). Outline of the pre-cavitated lesions (PCL) and cavitated occlusal lesions (COL) were carried out in MATLAB v7.12 to detect and classify caries<br>• Images were converted to grey-scale, PCL was segmented using K-means algorithm. COL was segmented within red, green and blue channel<br>• Selection range were 100<PCL<13,000 and 30<COL<900 and colour channel results were fused<br>• Caries classification was done by feature extraction, feature selection, and classification of pixel and region	Digital camera Canon Rebel XTi 10.1 MP with 100mm Macro EF lens and flash Macro Ring Light MR14 EX.<br>*In vitro images photography*: Digital camera O Olympus E-500; 8 MP with Olympus 50 mm macro lens and an Olympus 2xTeleconverter (EC-20). Shutter speed was 1/125 s and aperture F45 and Starblitz (Macrolite-1000 Auto) ring flashlight was used	• Low<br>(Different camera and setups were used for in-vivo and in-vitro photographs)	• The proposed method has 80% sensitivity, 74% precision and 80% accuracy in detection stage<br>• Random forest classifier showed the best result (83% accuracy).	Implemented a machine learned automated caries diagnostic system (ACD)	Computerised diagnostics provide more consistent, unbiased and accurate decision within a shorter time and trained as appropriate
Torlakovic, 2012 [137]	Experimental study (In vivo)	To determine which clinical condition predicted white spot lesions better in photographs	• Twenty-three premolars had orthodontic bands attached to influence plaque accumulation. The teeth were extracted after 7 weeks, and buccal surface were sectioned and photographed<br>• ImageJ software produced comparative colour histograms subtracting white spot lesion colour values from healthy enamel colour values<br>• SkyScan software determined the 3D volume of the white lesions from microcomputed tomography.	Digital camera D3X; Nikon with 200mm lens, shutter speed 1/125, aperture F/16 no flash was used	• Moderate<br>(The outcomes were influenced by patient dependent variables such as brushing habits, that were not controlled in the study)	• After 7 weeks, 30% of the sample did not develop clinical white spot lesion<br>• Microcomputed tomography grey scale values (0- 1.2931) were lower than healthy enamel	White spot lesions were captured from 3D models	Microcomputed tomography and photographic visualisation can predict the penetration of white spot lesions into enamel
Iwami, 2007 [138]	Experimental study (In vitro)	To visually evaluate the objectivity of caries removal by using a caries detector dye	• Caries detector dye was applied by three operators to dentin caries of 32 newly extracted molar teeth<br>• The dentin surface with colour matching sticker were photographed<br>• Adobe Photoshop V5.0 were used to calculate the CIELAB values of the dentine surface along with the colour matching sticker.<br>• Matrix elements were calculated using least square formula to correct the colour values. Colour measurements were taken by using colourimeter	Photographed by using CCD camera. The camera was placed in 45º angle and two light sources (Cold Spot, PCS-HRX-150; Nippon)	• Moderate<br>(Only carious molar teeth were selected. The samples were not equally distributed among the operators.<br>The samples were selected based on operators' visual judgement)	• Average ΔE value was within 4.7±2.9–14.6 ± 5.2 while average ΔL value was 2.5±2.1– 13.6±5.9 and were both similar to the colourimetry analysis.<br>• There was a significant difference (P<.05) present between intra and inter operator reproducibility	Photographic analyses were carried out on carious dentin coated with detector dye	Photographic evaluations suggest that caries detector dyes are less reliable in detecting carious extent with pulpal involvement

Table S10: Summary of findings for Orthodontics

Orthodontics

Author name (year)	Study design	Aim of study	Measurable outcomes with evaluation	Photographic apparatus used with calibrations	Certainty evaluation with Cochrane GRADEpro	Study outcomes	Novelty of the study	Clinical implications of the study
Berssenbrügge, 2015 [139]	Experimental study (In vivo)	Development of techniques to investigate facial asymmetry using shape and colour	• Facial surface was measured by Fringe projection technique which<br>• White balance was corrected by white light projection<br>• To reduce the colour noise Gaussian blur filter with filter radius 2 pixels were used<br>• Interactive closest point algorithm was used to mirror the point cloud and register into original cloud<br>• sRGB colour values were transformed into CIECLAB values by using 10º standard observer and D65 illuminant<br>• Visual analysis was done to evaluate symmetry and attractiveness. This was achieved by showing the image to the observers. At first, observers were asked to rate the image based on a visual analogue scale for its symmetry and after 1 week the same observers were asked to rate the image based on its attractiveness	One coloured video camera stationed in the centre and 2 monochrome cameras with a projector	• Moderate<br>(Ears and areas above hairline were excluded due to increased complexity<br>The subject age range was selected conveniently)	• Colour asymmetry index and geometric asymmetric index showed significant (P=0.017) correlation (r=0.433).<br>• On the other hand, geometric asymmetric index and symmetry assessments showed highest significant (P<0.001) correlation (r=0.378)	facial asymmetry was calculated from 3D scans on the basis of geometry and colour constancy	Virtual diagnostic tools for facial aesthetics can help in designing of craniofacial prosthesis
Jayaratn, 2012 [140]	Cohort study (In vivo)	To evaluate the feasibility of 3-D photos and CBCT images using root-mean-square error identification of an appropriate facial region for 3-D image registration	• 3D image obtained and processed by stereophotography<br>• CBCT scans were taken and surface registration was done with Iterative Closest Point (ICP) algorithm<br>• RMS values were compared with the colour maps	3dMDface stereophotography system (3dMD)	• High<br>(Inability to produce the same facial expression and facial muscle movement introduces variability and errors)	• Root-mean-square error of 0.739mm was evident for whole face	The use of a root-mean-square error with ICP to determine the accuracy differences in photography and CBCT images	3D models can assess bone and soft tissue thickness during diagnosis and treatment planning
Jayaratn, 2010 [141]	Case Series (In vivo)	To explore the application of colour maps in cranio-maxillofacial region	• Before-After 3D images were obtained by interpolating CT images with stereophotogrammetry data. The surgical effects were evaluated using colour-based discrepancies	*Software*: 3dMDpatient, 3dMDvultus (3dMD, Atlanta) and Maxilim (Medicim, Mechelen) was used for creating colour maps	• Very low<br>(Majority of the software for colour mapping is not scientifically validated)	• Soft tissue position between CBCT and 3D photo of + 5mm to – 5mm produced clinically noticeable changes<br>• For accurate colour mapping of anatomical landmarks in non-growing patients, the ideal reference points were the forehead, root of the nose, and zygoma due to early maturation	Distance between two superimposed images pre- and post-treatment was represented in a colour-mapped graphical format.	A Critical evaluation of facial asymmetry can be done with colours to maximise diagnostic information.<br>Colour maps can be a useful tool to identify the regions that may not be obvious otherwise

Brough,<br>2010 [142]	Cohort study (In vivo)	To evaluate whether maxillary canines' size or shape influence the perception of photographic smile attractiveness for patients where canines are used to substitute missing maxillary lateral incisors.	• Photographs of patients' smiles were obtained where maxillary canine was digitally modified and replaced by lateral incisors<br>• Photoshop CS2 software was used to create a bilateral symmetrical image.<br>• 31 images were created and divided into 4 groups. Every group had 1 altered variable (canine gingival height, crown tip height, width, and shade)<br>• Images were printed on standard colour photographic paper	Incomplete camera details were provided	• Low<br>(Only patients with hypodontia were selected for the study<br>120 observers were selected as participants including orthodontists, dentist and lay persons)	• Dentists and orthodontists suggested 1.5 mm narrow, rounded tip canines, with dark shade and low gingival margins to be photographically acceptable.<br>• Perception of photographic acceptability amongst lay persons varied greatly	Photographs were modified by software to produce various tooth shapes and sizes	Darker shade canines negatively affect photographic smile attractiveness.<br>Tooth morphology variations of within 0.5mm are acceptable and somewhat undetectable in photographs

Table S11: Details of the colour analysis devices that were used as criterion standards

Author, Year	Device used	Brand name	Company name	Function
Mahn, 2020 [58]	Spectrophotometer	VITA Easyshade®	VITA Zahnfabrik	Tooth and ceramic colour registration
Albert, 2019 [61]	Spectrophotometer	VITA Easyhade®	VITA Zahnfabrik	Tooth and ceramic colour registration
Mulcare, 2019 [120]	Spectrocolourimeter	Spectromatch	Spectrocolourimeter, Spectromatch, UK	Used for skin colour measurement
Yoon, 2018 [71]	Colourimeter	ShadeEye-NCC	ShadeEye-NCC chroma meter SHOFU Dental GmbH	Tooth and ceramic colour analysis
Mehl, 2017 [74]	Spectrophotometer	1. VITA Easyshade®<br>2. VITA Easyshade® Advance 4.0<br>3. SpectroShade device<br>4. SPECTROSHADE MICRO II	1. VITA Zahnfabrik<br>2. VITA Zahnfabrik<br>3. SpectroShade<br>4. MTH Optic Research AG	1. Tooth and ceramic colour registration<br>2. Tooth colour registration<br>3. Capture the image of face and mouth, also colour mapping can be done<br>4. Capture the mouth image perform the colour analysis within the image
Miyajiwala, 2017 [75]	Spectrophotometer	VITA Easyshade®	VITA Zahnfabrik	Tooth and ceramic colour registration
Rauber, 2017 [96]	Spectrophotometer	VITA Easyshade®	VITA EasyShade®, Vident, Brea, California	Used for matching tooth shade and also it can scan a tooth to find a matching shade. based on internationally established tooth shade system
Lakhanpal, 2016 [113]	Spectrophotometer	Datacolour® 650	Datacolour® 650 spectrophotometer	Used to evaluate the colour both in laboratory and production setting
Culic, 2014 [80]	Spectrophotometer	VITA Easyshade® Advanced	VITA Zahnfabrik	Tooth colour registration
Xiao, 2014 [126]	Spectrophotometer	Konica Minolta CM-2600d	Konica Minolta Investment ltd. China	This device was used for non-dental purposes.
Xiao, 2013 [128]	Spectrophotometer	Konica Minolta CM-2600d	Konica Minolta Investment ltd. China	The device was used for non-dental purposes.
Lasserre, 2011 [83]	Spectrophotometer	VITA Easyshade®	VITA Zahnfabrik	Tooth and ceramic colour registration

Delallea, 2011 [130]	Chromameter/ colourimeter	Chromameter CR-400	Chromameter CR400 Konica Minolta Investment ltd. China	This device was used for non-dental purposes g
Caglar, 2010 [87]	Colourimeter	ShadeEye-NCC	ShadeEye-NCC chroma meter SHOFU Dental GmbH	Tooth and ceramic colour analysis
Yamanel, 2010 [100]	Colourimeter	ShadeEye-NCC	ShadeEye-NCC chroma meter SHOFU Dental GmbH	Tooth and ceramic colour analysis
Schropp, 2009 [88]	Spectrophotometer	VITA Easyshade®	VITA EasyShade®, Vident, Brea, California	Colour determination of CAD/Cam material, ceramic restoration, tooth colour measurement
Pladellorens, 2008 [132]	Spectrophotometer	Konica Minolta CM-2002	Konica Minolta Investment ltd. China	This devices can be used for a variety of purposes including industrial applications
Iwami, 2007 [138]	Colourimeter	Konica Minolta CR-100	Konica Minolta Investment ltd. China	Food industry
Gadhia, 2006 [104]	Spectrophotometer	No name was provided by the author	_	_
Guan, 2005 [106]	Spectrophotometer	Konica Minolta CM-2600d	Konica Minolta Investment ltd. China	The device was used for non-dental purposes. Can measure the colour from leather, ball, paper
Wozniak, 1981 [118]	Colourimeter	IDL Colour Eye	Web information unavailable	Web information unavailable

Table S12: Newcastle-Ottawa scoring: reasons for further exclusion of papers

Author name	Article title	Score	Reason for exclusion
Almeida, 2021 [93]	Digital photography vs. clinical assessment of resin composite restorations	0	Authors did not compare non-proximity digital methods with measurements from a spectrophotometer, colourimeter nor conventional visual methods.
Garg, 2020 [94]	Masking white fluorotic spots using resin infiltration and its quantification by computerised photographic analysis: a 12-month follow-up study	1	Authors did not describe adequately the PICO 'Comparison' group.
Murro, 2020 [59]	The relationship of tooth shade and skin tone and its influence on the smile attractiveness	2	Authors adequately described defined group characteristics, but they did not use quantitative measurements to compared non-proximity methods with contact nor conventional visual methods
Yousof, 2020 [109]	Quantitative evaluation of masticatory performance with two-colour mixing ability test: development of a new digital method	1	Authors did not describe adequately n the characteristics of the PICO 'Comparison' group
Justiawan, 2019 [62]	Comparative analysis of a colour matching system for teeth recognition	0	Authors did not compare non-proximity digital methods with measurements from a spectrophotometer, colourimeter nor conventional visual methods.
Albert, 2019 [61]	Colour assessment in dental prostheses using smart phones ls	0	Authors did not compare non-proximity digital methods with measurements from a spectrophotometer, colourimeter nor conventional visual methods.
Lazar, 2019 [64]	The accuracy of dental shade matching using cross-polarised photography	0	Authors did not compare non-proximity digital methods with measurements from a spectrophotometer, colourimeter nor conventional visual methods.
Lin, 2019 [65]	An efficient image processing methodology based on fuzzy decision for dental shade matching	2	Authors adequately defined group characteristics but they compared non-proximity methods with contact or conventional visual methods without quantitative measurements
Sam, 2019 [110]	Evaluation of variation in shade in metal-ceramic restoration from the shade tab	1	Authors did not describe adequately the characteristics of the PICO 'Comparison' group
Ghanoum, 2019 [134]	Frame stitching in human oral cavity environment using intraoral camera	2	Authors adequately described defined group characteristics, but they did not use quantitative measurements to compared non-proximity methods with contact nor conventional visual methods
Sampaio, 2019 [66]	Variability of colour matching with different digital photography techniques and a grey reference card	1	Authors did not describe adequately the PICO 'Comparison' group
Mulcare, 2019 [120]	Suitability of a mobile phone colourimeter application for use as an objective aid when matching skin colour during the fabrication of a maxillofacial prosthesis	0	Authors did not compare non-proximity digital methods with measurements from a spectrophotometer or colourimeter nor conventional visual methods

Maddula, 2018 [67]	Comparison of shade matching photos taken with grey card and without grey card	1	Authors did not describe adequately of the PICO ‘Comparison’ group
Lam, 2018 [68]	Mapping intraoral photographs on virtual teeth model	0	Authors did not compare non-proximity digital methods with measurements from a spectrophotometer, colourimeter nor conventional visual methods
Sampaio, 2018 [69]	Dental shade guide variability for Hues B, C and D using Cross-polarized photography	0	Authors did not compare non-proximity digital methods with measurements from a spectrophotometer or colourimeter nor conventional visual methods
Kim, 2018 [70]	A digital shade-matching device for dental colour determination using the support vector machine algorithm	1	Authors did not describe adequately the PICO ‘Comparison’ group
Sohaib, 2018 [122]	Colour quality of facial prostheses in additive manufacturing	0	Authors did not compare non-proximity digital methods with measurements from a spectrophotometer or colourimeter nor conventional visual methods
Sadek, 2018 [111]	Different materials used as denture retainers and their colour stability	1	Authors did not describe adequately the PICO ‘Comparison’ group
Mayer, 2017 [135]	Photometric CIELAB analysis of the gingiva: a novel approach to assess response to periodontal therapy	1	Authors did not describe adequately the characteristics of the PICO ‘Comparison’ group
Luo, 2017 [73]	The in vitro and in vivo reproducibility of a video-based digital imaging system for tooth colour measurement	2	Authors adequately described defined group characteristics, but they did not use quantitative measurements to compared non-proximity methods with contact or conventional visual methods without quantitative measurements
Tam, 2017 [76]	Accurate shade image matching by using a smartphone camera	0	Authors did not compare non-proximity digital methods with measurements from a spectrophotometer, colourimeter nor conventional visual methods
Carney, 2016 [77]	A novel regression model from RGB image data to spectroradiometric correlates optimized for tooth coloured shades	1	Authors did not describe adequately the characteristics of the PICO ‘Comparison’ group
Kim, 2016 [112]	Differences in the intensity of light-induced fluorescence emitted by resin composites	0	Authors did not compare non-proximity digital methods with measurements from a spectrophotometer, colourimeter nor conventional visual methods
Gurrea, 2016 [78]	Evaluation of dental shade guide variability using Cross-polarised photography	1	Authors did not describe adequately the characteristics of the PICO ‘Comparison’ group
Hein, 2016 [79]	The use of a standardized grey reference card in dental photography to correct the effects of five commonly used diffusers on the colour of 40 extracted teeth	1	Authors did not describe adequately the characteristics of the PICO ‘Comparison’ group
Pricop, 2016 [123]	Original alternative technique of iris fabrication for ocular prostheses	2	Authors adequately described defined group characteristics, but they did not use quantitative measurements to compared non-proximity methods with contact or conventional visual methods
Grant, 2015 [124]	Digital capture, design, and manufacturing of a facial prosthesis: Clinical report on a pediatric patient	0	Authors did not compare non-proximity digital methods with measurements from a

			spectrophotometer, colourimeter nor conventional visual methods
Berdouses, 2015 [136]	A computer-aided automated methodology for the detection and classification of occlusal caries from photographic colour images	1	Authors did not describe adequately the characteristics of the PICO 'Comparison' group
Buzayan, 2015 [125]	Ocular defect rehabilitation using photography and digital imaging: a clinical report	0	Authors did not compare non-proximity digital methods with measurements from a spectrophotometer, colourimeter nor conventional visual methods
Irawan, 2013 [97]	In-vitro computer analysis of digital images on tooth- coloured restorative material using L*a*b* values	1	Authors did not describe adequately the PICO 'Comparison' group
Tan, 2013 [127]	Colour detection thresholds in faces and colour patches	0	Authors did not compare non-proximity digital methods with measurements from a spectrophotometer, colourimeter nor conventional visual methods
Takatsui, 2012 [98]	CIE L*a*b*: comparison of digital images obtained photographically by manual and automatic modes	1	Authors did not describe adequately the PICO 'Comparison' group
Torlakovic, 2012 [137]	Clinical colour intensity of white spot lesions might be a better predictor of enamel demineralisation depth than traditional clinical grading	1	Authors did not describe adequately the characteristics of the PICO 'Comparison' group
Jayaratn, 2012 [140]	Study comparing the accuracy of cone beam computed tomography with f 3-D stereophotographic images?	0	Authors did not compare non-proximity digital methods with measurements from a spectrophotometer, or colourimeter nor conventional visual methods
Vafaii, 2012 [115]	Evaluation of colour and contour matching accuracy with digital photography and direct vision	2	Authors adequately described defined group characteristics, but they did not use quantitative measurements to compared non-proximity methods with contact nor conventional visual methods
Bilt, 2012 [114]	Digital image processing compared with visual assessment of chewed two-colour wax	0	Authors did not compare non-proximity digital methods with measurements from a spectrophotometer, or colourimeter nor conventional visual methods
Seo, 2011 [129]	Better understanding of digital photography for skin colour measurement	1	Authors did not describe adequately the characteristics of the PICO 'Comparison' group
Denissen, 2010 [86]	Photometric assessment of tooth colour using commonly available software	0	Authors did not compare non-proximity digital methods with measurements from a spectrophotometer or colourimeter nor conventional visual methods
Jayaratn, 2010 [141]	Three-dimensional colour maps: for assessing craniofacial changes	0	Authors did not compare non-proximity digital methods with measurements from a spectrophotometer or colourimeter nor conventional visual methods
Brough, 2010 [142]	Canine substitution for missing maxillary lateral incisors: The influence of canine morphology, size, and shade on perceptions of smile attractiveness	2	Authors adequately described defined group characteristics, but they did not use quantitative measurements to compared non-proximity methods with contact nor conventional visual methods

Jain, 2010 [131]	Prosthetic rehabilitation of ocular defect using digital photography	0	Authors did not compare non-proximity digital methods with measurements from a spectrophotometer, colourimeter nor conventional visual methods
Kuzekanani, 2009 [101]	Quantitative analysis of KTP laser photodynamic bleaching of tetracycline-discoloured teeth	0	Authors did not compare non-proximity digital methods with measurements from a spectrophotometer or colourimeter nor conventional visual methods
Pladellorens, 2008 [132]	A device for the colour measurement and detection of spots on the skin	0	Authors did not compare non-proximity digital methods with measurements from a spectrophotometer or colourimeter nor conventional visual methods
Smith, 2008 [89]	In vitro and in vivo validation of a mobile non-contact camera-based digital imaging system for tooth colour measurement	1	Authors did not describe adequately the characteristics of the PICO 'Comparison' group
Cal, 2007 [117]	Digital analysis of staining on acrylic resins caused by mouth rinses	1	Authors did not describe adequately the PICO 'Comparison' group
Lee, 2007 [103]	Subjective and photometric determination of bleaching outcomes	1	Authors did not describe adequately the PICO 'Comparison' group
Cal, 2004 [92]	Application of a digital technique in evaluating the reliability of shade guides	1	Authors did not describe adequately the PICO 'Comparison' group
Tao, 2003 [133]	A colour-based face detection system using multiple templates	2	Authors adequately described group characteristics, but they did not use quantitative measurements to compare non-proximity methods with contact nor conventional visual methods
Tsuzuki, 2002 [107]	Evaluation of intraoral charge-coupled device (CCD) camera for dental examination for forensic purposes	1	Authors did not describe adequately the PICO 'Comparison' group
Bentley, 1999 [108]	Quantitation of vital bleaching by computer analysis of photographic images	1	Authors did not describe adequately the PICO 'Comparison' group
Wozniak, 1981 [118]	Photographic assessment of colour changes in cold and heat-cure resins	2	Authors adequately defined group characteristics but they compared non-proximity methods with contact or conventional visual methods without quantitative measurements
Mccarty, 1976 [119]	Intraoral infrared colour photography of radiotherapy patients	0	Authors did not compare non-proximity digital methods with measurements from a spectrophotometer nor colourimeter nor conventional visual methods

Table S13: Camera oriented preparation used for shade taking

Modifiable factors	Authors	Most frequently used calibration	Less frequently used
**Digital camera lens**	Mahn, 2020; He, 2019; Lazar, 2019; Sampaio, 2019; Lam, 2018; Sampaio, 2018; Labban, 2017; Gurrea, 2016; Culic, 2014; Tung, 2010; Schropp, 2008; Smith, 2008; Wee, 2005; Almeida, 2020; Garg, 2020; Kim, 2017; Rauber, 2017; Takatsui, 2012; Jarad, 2008; Lath, 2006; Tsuzuki, 2002; Bentley, 1999; Sam, 2019; Kim, 2016; Lakhanpal, 2016; Won-suk Oh, 2010; Maccarty, 1976; Pricop, 2016; Seo, 2011; Mayer, 2017; Berdouse, 2015; Torlakovic, 2012. **(n = 32)**	50 mm to 105 mm Macro lens (81.25% of authors)<br><br>Lens manufacturer:<br>• Nikon (43.75%)<br>• Canon (28.13%)	1. 120 mm to 200 mm Macro lens (9.38 % of authors)<br>2. 18-28mm/others (3.13% of authors)<br><br>Lens manufacturer:<br>• Sony (3.13%),<br>• Sigma (6.25%),<br>• others (12.5%)
**Focal length**	Mahn, 2020; He, 2019; Lazar, 2019; Sampaio, 2019; Lam, 2018; Sampaio, 2018; Labban, 2017; Gurrea, 2016; Culic, 2014; Tung, 2010; Schropp, 2008; Smith, 2008; Wee, 2005; Almeida, 2020; Garg, 2020; Kim, 2017; Rauber, 2017; Takatsui, 2012; Jarad, 2008; Lath, 2006; Tsuzuki, 2002; Bentley, 1999; Sam, 2019; Kim, 2016; Lakhanpal, 2016; Won-suk Oh, 2010; Maccarty, 1976; Pricop, 2016; Seo, 2011; Mayer, 2017; Berdouse, 2015; Torlakovic, 2012. **(n = 32)**	f13 to f22 (46.88% of authors)	f2.8 to f11 (31.25% of authors)
**White balance control**	Mahn, 2020; He, 2019; Sampaio, 2019; Maddula, 2018; Luo, 2017; Miyajiwala, 2017; Hein, 2016; Tung, 2010; Denissen, 2010; Seo, 2011; Mayer, 2017; Berssenbrügge, 2015; Irawan, 2013; Tam, 2012; Sluzker, 2011; Wozniak, 1981; Pricop, 2016; Pladellorens, 2008; Tao, 2003. **(n = 19)**	White/Grey reference cards (68.42% of authors)	Colour checkers (5.26% of authors)
**Colour temperature**	Kim, 2018; Yoon, 2016; Tam, 2016; Tam, 2012; Lasserre, 2011; Caglar, 2009; Schropp, 2008; Yamanel, 2010; Guan, 2003; Wozniak, 1981; Sohaib, 2018; Seo, 2011. **(n = 12)**	5332K to 6500K (58.33% of authors)	1. 2700K to 4800 K (41.67% of authors)<br>2. Others (16.67% of authors)
**Illumination source**	Mahn, 2020; Murro, 2019; Liu, 2019; Labban, 2017; Miyajiwa la, 2017; Carney, 2016; Tam, 2016; Caglar, 2009; Jarad, 2005; Rauber, 2017; Yamanel, 2010; Jarad, 2008; Won-suk Oh, 2010; Albert, 2019. **(n = 14)**	1. External Ring Flash system (21.43% of authors)<br>2. External Twin Flash system (14.29% of authors)	1. Natural light/daylight (21.43% of authors)<br>2. External flashlight (14.29% authors)<br>3. Fluorescent lamps (21.43% authors)
**Light and colour filters**	Mahn, 2020; Albert, 2019; He, 2019; Lazar, 2019; Sampaio, 2019; Sampaio, 2018; Luo, 2017; Gurrea, 2016; Smith, 2008; Kim, 2016; Wozniak, 1981; Maccarty, 1976; Seo, 2011. **(n = 13)**	Polarizing or cross polarising filters (57.14% of authors)	1. Other types of filters (42.86% of authors)<br>2. Not specified (7.14% of authors)

**Background of the subject**	Lindsey, 2010; Schropp, 2008; Smith, 2008; Cal, 2004; Takatsui, 2012; Lath, 2006; Bentley, 1999; Won-suk Oh, 2010; Mulcare, 2018. **(n = 9)**	1. White background (33.3% of authors)<br>2. Grey background (33.3% of authors)	1. Black background (22.2% of authors)<br>2. Achromatic or green backgrounds (22.2% of authors)
**Computerised colour calibration**	Mahn, 2020; Liu, 2019; Lazar, 2019; Sampaio, 2019; Maddula, 2018; Lam, 2018; Sampaio, 2018; Miyajiwala, 2017; Gurrea, 2016; Culic, 2014; Tam, 2012; Tung, 2010; Denissen , 2010; Caglar, 2009; Schropp, 2008; Jarad, 2005; Wee, 2005; Cal, 2004; Almeida, 2020; Yamanel, 2010; Jarad, 2008; Bentley, 1999; Yousof, 2019; Sam, 2019; Sadek, 2018; Kim, 2016; Lakhanpa l, 2016; Won-suk Oh, 2010; Cal, 2007; Xiao, 2014; Buzayan, 2014; Tan, 2013; Xiao, 2013; Jain, 2010; Ghanoum, 2019; Berssenbrügge, 2015;Torlakovic, 2012; Jayaratn, 2010; Brough, 2010. **(n = 39)**	Adobe Photoshop photo editing software (Adobe Photoshop Inc and Adobe Photoshop Lightroom Inc) (38.46% of authors)	1. Matlab (12.82% of authors)<br>2. Image-J software (10.26% of authors)<br>3. Paint Shop Pro (PSP) Graphic software (7.69% of authors)<br>4. Macbeth colour checker, Easy RGB, colour meter (5.13%)<br>5. Others (28.21% of authors)

Percentage is based on the number of authors that implemented the calibration to their research design. Some authors successfully used two or more calibrations and therefore resulted in an overlap in % evaluations

References:

[1] N. A. Baykan and N. Yılmaz, “Case study in effects of color spaces for mineral identification,” *Sci. Res. essays*, vol. 5, no. 11, pp. 1243–1253, 2010.

[2] J. Souza, M. Fuentes, E. Baena, and L. Ceballos, “One-year clinical performance of lithium disilicate versus resin composite CAD/CAM onlays,” *Odontology*, vol. 109, no. 1, pp. 259–270, 2021.

[3] M. Sanchez-Garcia, R. Martinez-Cantin, and J. J. Guerrero, “Semantic and structural image segmentation for prosthetic vision,” *PLoS One*, vol. 15, no. 1, p. e0227677, 2020.

[4] S. H. Kabil, M. F. Haridy, and M. R. Farid, “Effect of high light intensity bleaching protocol versus descending light intensities bleaching protocol on post bleaching teeth sensitivity: A randomized clinical trial,” *Open Access Maced. J. Med. Sci.*, vol. 7, no. 13, p. 2173, 2019.

[5] B. Askarian, F. Tabei, G. A. Tipton, and J. W. Chong, “Smartphone-Based Method for Detecting Periodontal Disease,” in *2019 IEEE Healthcare Innovations and Point of Care Technologies,(HI-POCT)*, IEEE, 2019, pp. 53–55.

[6] C. A. Villavicencio-Espinoza, M. H. Narimatsu, and A. Y. Furuse, “Using cross-polarized photography as a guide for selecting resin composite shade,” *Oper. Dent.*, vol. 43, no. 2, pp. 113–120, 2018.

[7] L. G. Gavinho, S. A. Araujo, S. K. Bussadori, J. V. P. Silva, and A. M. Deana, “Detection of white spot lesions by segmenting laser speckle images using computer vision methods,” *Lasers Med. Sci.*, vol. 33, no. 7, pp. 1565–1571, 2018.

[8] F. Rojas Vizcaya, “Retrospective 2-to 7-year follow-up study of 20 double full-arch implant-supported monolithic zirconia fixed prostheses: measurements and recommendations for optimal design,” *J. Prosthodont.*, vol. 27, no. 6, pp. 501–508, 2018.

[9] I. Brokos, M. Stavridakis, and I. Krejci, “Digital photographic procedure for comprehensive two-dimensional tooth shade analysis,” *Compend. Contin. Educ. Dent.*, vol. 38, no. 8, pp. e1–e4, 2017.

[10] S. Hein, J. Tapia, and P. Bazos, “eLABor_aid: a new approach to digital shade

management," *Int J Esthet Dent*, vol. 12, no. 2, pp. 186–202, 2017.

[11] E. A. McLaren, J. Figueira, and R. E. Goldstein, "A technique using calibrated photography and photoshop for accurate shade analysis and communication," *Compendium*, vol. 38, no. 2, pp. 106–113, 2017.

[12] N. Sharmili, N. Swapna, and G. Ramakrishna, "Comparative analysis of image processing algorithms for visual prosthesis," in *2017 International Conference on Communication and Signal Processing (ICCSP)*, IEEE, 2017, pp. 1120–1124.

[13] S. V Pavlov *et al.*, "Electro-optical system for the automated selection of dental implants according to their colour matching," *Przegląd Elektrotechniczny*, vol. 93, no. 3, pp. 121–124, 2017.

[14] C. Coachman, M. A. Calamita, and N. Sesma, "Dynamic documentation of the smile and the 2D/3D digital smile design process," *Int J Periodontics Restor. Dent*, vol. 37, no. 2, pp. 183–193, 2017.

[15] Y. Chen, J. Fu, D. Chu, R. Li, and Y. Xie, "An image-processing strategy to extract important information suitable for a low-size stimulus pattern in a retinal prosthesis," *Biomed. Eng. Tech.*, vol. 62, no. 6, pp. 591–598, 2017.

[16] T. B. Brandão and A. J. Vechiato Filho, "Technique for preserving pupil size when photographing the iris for ocular prostheses," *J. Prosthet. Dent.*, vol. 115, no. 4, p. 509, 2016.

[17] J. Watson and M. M. Hatamleh, "Complete integration of technology for improved reproduction of auricular prostheses," *J. Prosthet. Dent.*, vol. 111, no. 5, pp. 430–436, 2014.

[18] S. Elhabian, A. Abdelrahim, A. Farag, D. Tasman, W. Aboelmaaty, and A. Farman, "Clinical crowns shape reconstruction-an image-based approach," in *2013 IEEE 10th International Symposium on Biomedical Imaging*, IEEE, 2013, pp. 93–96.

[19] J. F. Walder, K. Freeman, M. J. Lipp, O. F. Nicolay, and G. J. Cisneros, "Photographic and videographic assessment of the smile: objective and subjective evaluations of posed and spontaneous smiles," *Am. J. Orthod. Dentofac. Orthop.*, vol. 144, no. 6, pp. 793–801, 2013.

[20] A. Salat, W. Devoto, and J. Manauta, "Achieving a precise color chart with common

computer software for excellence in anterior composite restorations," *Eur J Esthet Dent*, vol. 6, no. 3, pp. 280–296, 2011.

[21] M. M. Hatamleh and D. C. Watts, "Porosity and color of maxillofacial silicone elastomer," *J. Prosthodont. Implant. Esthet. Reconstr. Dent.*, vol. 20, no. 1, pp. 60–66, 2011.

[22] S. Kim-Pusateri, J. D. Brewer, E. L. Davis, and A. G. Wee, "Reliability and accuracy of four dental shade-matching devices," *J. Prosthet. Dent.*, vol. 101, no. 3, pp. 193–199, 2009.

[23] S. F. Rosenstiel, M. Pappas, M. T. Pulido, and R. G. Rashid, "Quantification of the esthetics of dentists' before and after photographs," *J. Dent.*, vol. 37, pp. e64–e69, 2009.

[24] C. H. Silva-Lovato, A. M. G. Totti, H. F. O. Paranhos, and V. G. Totti, "Evaluation of a Computerized Method for Denture Biofilm Quantification: Inter-Examiner Reproducibility," *J. Prosthodont. Implant. Esthet. Reconstr. Dent.*, vol. 18, no. 4, pp. 332–336, 2009.

[25] J. D. Griffin Jr, "Use of digital photography to improve composite resin selection and material placement," *Pract. Proced. Aesthetic Dent.*, vol. 20, no. 6, pp. 359–364, 2008.

[26] S. J. Chu, "Clinical steps to predictable color management in aesthetic restorative dentistry," *Dent. Clin. North Am.*, vol. 51, no. 2, pp. 473–485, 2007.

[27] J. D. Griffin Jr, "Assessing aesthetic composite veneer placement via digital photography," *Pr. Proced Aesthet Dent*, vol. 19, no. 5, pp. 289–294, 2007.

[28] L. J. Stumpel 3rd, "Simplifying the correction of the digital image in shade communication.," *J. Prosthet. Dent.*, vol. 92, no. 2, pp. 202–203, 2004.

[29] L. M. Sykes, A. M. Parrott, C. P. Owen, and D. R. Snaddon, "Applications of rapid prototyping technology in maxillofacial prosthetics," *Int. J. Prosthodont.*, vol. 17, no. 4, 2004.

[30] W. M. Bengel, "Digital photography and the assessment of therapeutic results after bleaching procedures," *J. Esthet. Restor. Dent.*, vol. 15, pp. S21–S32, 2003.

[31] D. Y. Ng, J. P. Allebach, M. Analoui, and Z. Pizio, "Non-contact imaging colorimeter

for human tooth color assessment using a digital camera (vol 47, pg 531, 2003)," *J. IMAGING Sci. Technol.*, vol. 48, no. 4, pp. IV–IV, 2004.

[32] W. K. Dancy, P. Yaman, J. B. Dennison, W. J. O'BRIEN, and M. E. Razzoog, "Color measurements as quality criteria for clinical shade matching of porcelain crowns," *J. Esthet. Restor. Dent.*, vol. 15, no. 2, pp. 114–122, 2003.

[33] S. Phelan, "Use of photographs for communicating with the laboratory in indirect posterior restorations," *Journal-Canadian Dent. Assoc.*, vol. 68, no. 4, pp. 239–242, 2002.

[34] D. Dirksen, C. Runte, Z. Böröcz, C. Thomas, G. von Bally, and F. Bollmann, "Three-dimensional quantification of color-marked occlusal paths on anatomically oriented casts," *J. Prosthet. Dent.*, vol. 85, no. 2, pp. 156–161, 2001.

[35] M.-C. Juan, M. Alcañiz, C. Monserrat, V. Grau, and C. Knoll, "Computer-aided periodontal disease diagnosis using computer vision," *Comput. Med. imaging Graph.*, vol. 23, no. 4, pp. 209–217, 1999.

[36] D. Inaba, O. Takagi, and J. Arends, "A computer-assisted videodensitometric method to visualize mineral distributions in in vitro and in vivo formed root caries lesions," *Eur. J. Oral Sci.*, vol. 105, no. 1, pp. 74–80, 1997.

[37] R. C. Swineford, A. O. Wist, and S. L. Herr, "Digital transillumination technique for incipient lesions on teeth," in *Clinical Applications of Modern Imaging Technology II*, SPIE, 1994, pp. 201–207.

[38] M. I. Gazi, "Photographic assessment of the antiplaque properties of sanguinarine and chlorhexidine," *J. Clin. Periodontol.*, vol. 15, no. 2, pp. 106–109, 1988.

[39] H.-J. Chung, S.-A. Lim, H.-K. Lim, and S.-K. Jung, "Perceptual and quantitative analysis of discoloration of orthodontic elastomeric chains by food," *BMC Oral Health*, vol. 23, no. 1, pp. 1–8, 2023.

[40] O. Ginesin, H. Zigdon-Giladi, E. Gabay, E. E. Machtei, E. Mijiritsky, and Y. Mayer, "Digital photometric analysis of gingival response to periodontal treatment," *J. Dent.*, vol. 127, p. 104331, 2022.

[41] F. Rashid *et al.*, "Color variations during digital imaging of facial prostheses subjected to unfiltered ambient light and image calibration techniques within dental clinics: An

in vitro analysis," *PLoS One*, vol. 17, no. 8, p. e0273029, 2022.

[42] G. J. Jorquera *et al.*, "A comparison of ceramic crown color difference between different shade selection methods: visual, digital camera, and smartphone," *J. Prosthet. Dent.*, vol. 128, no. 4, pp. 784–792, 2022.

[43] N. Sirintawat, T. Leelaratrungruang, P. Poovarodom, S. Kiattavorncharoen, and P. Amornsettachai, "The accuracy and reliability of tooth shade selection using different instrumental techniques: An in vitro study," *Sensors*, vol. 21, no. 22, p. 7490, 2021.

[44] J. L. B. de Abreu, C. S. Sampaio, E. B. Benalcazar Jalkh, and R. Hirata, "Analysis of the color matching of universal resin composites in anterior restorations," *J. Esthet. Restor. Dent.*, vol. 33, no. 2, pp. 269–276, 2021.

[45] D. L. Duong, M. H. Kabir, and R. F. Kuo, "Automated caries detection with smartphone color photography using machine learning," *Health Informatics J.*, vol. 27, no. 2, p. 14604582211007530, 2021.

[46] K. Ebeid, A. Sabet, and A. Della Bona, "Accuracy and repeatability of different intraoral scanners on shade determination," *J. Esthet. Restor. Dent.*, vol. 33, no. 6, pp. 844–848, 2021.

[47] S. Hein, D. Modrić, S. Westland, and M. Tomeček, "Objective shade matching, communication, and reproduction by combining dental photography and numeric shade quantification," *J. Esthet. Restor. Dent.*, vol. 33, no. 1, pp. 107–117, 2021.

[48] J. Jauregui Ulloa, R. Salazar-Gamarra, A. M. M. Mesquita, F. Aguirre, and L. L. Dib, "A Digital Method to Fabricate the Ocular Portion of An Orbital Prosthesis with A Smartphone Camera, Color Calibration and Digital Printing," *J. Prosthodont.*, vol. 30, no. 1, pp. 91–94, 2021.

[49] A. Mohammadi, Z. Bakhtiari, F. Mighani, and F. Bakhtiari, "Validity and reliability of tooth color selection by smartphone photography and software applications," *J. Indian Prosthodont. Soc.*, vol. 21, no. 3, p. 281, 2021.

[50] J. T. Newton, S. S. Subramanian, S. Westland, A. K. Gupta, W. Luo, and A. Joiner, "The impact of tooth colour on the perceptions of age and social judgements.," *J. Dent.*, vol. 112, p. 103771, 2021.

[51] F. B. Raza, V. A. Kumar, A. Jacob, and A. An, "Assessment of a smartphone-based

software application as a potential digital tool in tooth shade selection: A prospective clinical study," *Quintessence Int. (Berl).*, vol. 52, no. 7, pp. 608–617, 2021, doi: 10.3290/j.qi.b1044157.

[52] S. Shehadeh, L. Levi, and D. Shamir, "Clinical evaluation of a novel radiofrequency-based toothbrush for teeth whitening and reduction of teeth stains: A pilot study," *Clin. Exp. Dent. Res.*, vol. 7, no. 6, pp. 1096–1102, 2021.

[53] M. Soldo, D. Illeš, R. Ćelić, and D. Knezović Zlatarić, "Assessment of Color Parameters on Maxillary Right Central Incisors Using Spectrophotometer and RAW Mobile Photos in Different Light Conditions," *Acta Stomatol. Croat.*, vol. 54, no. 4, pp. 353–362, 2020.

[54] L. Kalman, "Development of a novel dental shade determination application," *J. Dent. Res. Dent. Clin. Dent. Prospects*, vol. 14, no. 1, p. 73, 2020.

[55] K. Kelkar, E. Dogra, V. Bhat, D. Prasad, and C. Hegde, "A comparison between visual, digital photography and polarizing filter photography for shade selection," *Indian J. Dent. Res.*, vol. 31, no. 5, pp. 712–717, 2020, doi: 10.4103/ijdr.IJDR_286_19.

[56] S. P. Suganya, P. Manimaran, D. Saisadan, C. D. Kumar, D. Abirami, and V. Monnica, "Spectrophotometric evaluation of shade selection with digital and visual methods," *J. Pharm. Bioallied Sci.*, vol. 12, no. Suppl 1, p. S319, 2020.

[57] V. Rutkūnas, J. Dirsė, and V. Bilius, "Accuracy of an intraoral digital scanner in tooth color determination," *J. Prosthet. Dent.*, vol. 123, no. 2, pp. 322–329, 2020.

[58] E. Mahn, S. C. Tortora, B. Olate, F. Cacciuttolo, J. Kernitsky, and G. Jorquera, "Comparison of visual analog shade matching, a digital visual method with a cross-polarized light filter, and a spectrophotometer for dental color matching," *J. Prosthet. Dent.*, 2020.

[59] B. Di Murro, G. Gallusi, R. Nardi, A. Libonati, V. Angotti, and V. Campanella, "The relationship of tooth shade and skin tone and its influence on the smile attractiveness," *J. Esthet. Restor. Dent.*, vol. 32, no. 1, pp. 57–63, 2020.

[60] W. He, C. J. Park, S. Byun, D. Tan, C. Y. Lin, and W. Chee, "Evaluating the relationship between tooth color and enamel thickness, using twin flash photography,

cross-polarization photography, and spectrophotometer," *J. Esthet. Restor. Dent.*, vol. 32, no. 1, pp. 91–101, 2020.

[61] C. J. Albert, E. N. Da Silva, M. M. Penteado, D. R. de Lima, E. T. Kimpara, and E. S. Uemura, "Color assessment in dental prostheses: the use of smartphones as process tools," *Brazilian Dent. Sci.*, vol. 22, no. 4, pp. 573–577, 2019.

[62] D. A. W. Justiawan, R. P. Hadi, A. P. Nurhayati, K. Prayogo, R. Sigit, and Z. Arief, "Comparative Analysis of Color Matching System for Teeth Recognition Using Color Moment," *Med. Devices (Auckland, NZ)*, vol. 12, p. 497, 2019.

[63] Y. Liu *et al.*, "The development of a 3D colour reproduction system of digital impressions with an intraoral scanner and a 3D printer: a preliminary study," *Sci. Rep.*, vol. 9, no. 1, pp. 1–10, 2019.

[64] R. Lazar, B. Culic, C. Gasparik, C. Lazar, and D. Dudea, "The accuracy of dental shade matching using cross-polarization photography.," *Int. J. Comput. Dent.*, vol. 22, no. 4, pp. 343–351, 2019.

[65] T.-L. Lin *et al.*, "An efficient image processing methodology based on fuzzy decision for dental shade matching," *J. Intell. Fuzzy Syst.*, vol. 36, no. 2, pp. 1133–1142, 2019.

[66] C. S. Sampaio, P. J. Atria, R. Hirata, and G. Jorquera, "Variability of color matching with different digital photography techniques and a gray reference card," *J. Prosthet. Dent.*, vol. 121, no. 2, pp. 333–339, 2019.

[67] R. T. Maddula, A. Abraham, P. Ariga, and A. R. Jain, "Comparison of shade matching photos taken with gray card and without gray card.," *Drug Invent. Today*, vol. 10, no. 9, 2018.

[68] W. Y. H. Lam, R. T. C. Hsung, L. Y. Y. Cheng, and E. H. N. Pow, "Mapping intraoral photographs on virtual teeth model," *J. Dent.*, vol. 79, pp. 107–110, 2018.

[69] C. S. Sampaio *et al.*, "Dental shade guide variability for hues B, C, and D using cross-polarized photography," *Int J Periodontics Restor. Dent*, vol. 38, pp. 113–118, 2018.

[70] M. Kim *et al.*, "A digital shade-matching device for dental color determination using the support vector machine algorithm," *Sensors*, vol. 18, no. 9, p. 3051, 2018.

[71] H. Yoon, J. Bae, J. Park, Y. Chun, M. Kim, and M. Kim, "A study on possibility of

clinical application for color measurements of shade guides using an intraoral digital scanner," *J. Prosthodont.*, vol. 27, no. 7, pp. 670–675, 2018.

[72] N. Labban, H. Al-Otaibi, A. Alayed, K. Alshankiti, and M. A. Al-Enizy, "Assessment of the influence of gender and skin color on the preference of tooth shade in Saudi population," *Saudi Dent. J.*, vol. 29, no. 3, pp. 102–110, 2017.

[73] W. Luo *et al.*, "The in vitro and in vivo reproducibility of a video-based digital imaging system for tooth colour measurement," *J. Dent.*, vol. 67, pp. S15–S19, 2017.

[74] A. Mehl, G. Bosch, C. Fischer, and A. Ender, "In vivo tooth-color measurement with a new 3D intraoral scanning system in comparison to conventional digital and visual color determination methods," *Int J Comput Dent*, vol. 20, no. 4, pp. 343–361, 2017.

[75] J. S. Miyajiwala, M. G. Kheur, A. H. Patankar, and T. A. Lakha, "Comparison of photographic and conventional methods for tooth shade selection: A clinical evaluation," *J. Indian Prosthodont. Soc.*, vol. 17, no. 3, p. 273, 2017.

[76] W.-K. Tam and H.-J. Lee, "Accurate shade image matching by using a smartphone camera," *J. Prosthodont. Res.*, vol. 61, no. 2, pp. 168–176, 2017.

[77] M. N. Carney and W. M. Johnston, "A novel regression model from RGB image data to spectroradiometric correlates optimized for tooth colored shades," *J. Dent.*, vol. 51, pp. 45–48, 2016.

[78] J. Gurrea *et al.*, "Evaluation of Dental Shade Guide Variability Using Cross-Polarized Photography.," *Int. J. Periodontics Restorative Dent.*, vol. 36, no. 5, 2016.

[79] S. Hein and M. Zangl, "The use of a standardized gray reference card in dental photography to correct the effects of five commonly used diffusers on the color of 40 extracted human teeth," *Int J Esthet Dent*, vol. 11, no. 2, pp. 246–259, 2016.

[80] B. Culic, V. Prejmerean, C. Gasparik, C. Culic, C. Dragos, and D. Dudea, "In vitro evaluation of new dental software for shade matching," in *Key Engineering Materials*, Trans Tech Publ, 2014, pp. 360–365.

[81] J. Montero, C. Gómez-Polo, J. A. Santos, M. Portillo, M. C. Lorenzo, and A. Albaladejo, "Contributions of dental colour to the physical attractiveness stereotype," *J. Oral Rehabil.*, vol. 41, no. 10, pp. 768–782, 2014.

[82] W. K. Tam and H. J. Lee, “Dental shade matching using a digital camera,” *J. Dent.*, vol. 40, pp. e3–e10, 2012.

[83] J.-F. Lasserre, I.-S. Pop-Ciutrila, and H.-A. Colosi, “A comparison between a new visual method of colour matching by intraoral camera and conventional visual and spectrometric methods,” *J. Dent.*, vol. 39, pp. e29–e36, 2011.

[84] O.-H. Tung, Y.-L. Lai, Y.-C. Ho, I.-C. Chou, and S.-Y. Lee, “Development of digital shade guides for color assessment using a digital camera with ring flashes,” *Clin. Oral Investig.*, vol. 15, no. 1, pp. 49–56, 2011.

[85] D. T. Lindsey and A. G. Wee, “Assessing tooth color differences in digital facial portraits,” *J. Dent. Res.*, vol. 89, no. 11, pp. 1254–1258, 2010.

[86] H. Denissen and A. Dozic, “Photometric assessment of tooth color using commonly available software,” *Eur J Esthet Dent*, vol. 5, no. 2, pp. 204–215, 2010.

[87] A. Caglar, K. Yamanel, K. Gulsahi, B. Bagis, and M. Özcan, “Could digital imaging be an alternative for digital colorimeters?,” *Clin. Oral Investig.*, vol. 14, no. 6, pp. 713–718, 2010.

[88] L. Schropp, “Shade matching assisted by digital photography and computer software,” *J. Prosthodont. Implant. Esthet. Reconstr. Dent.*, vol. 18, no. 3, pp. 235–241, 2009.

[89] R. N. Smith *et al.*, “The in vitro and in vivo validation of a mobile non-contact camera-based digital imaging system for tooth colour measurement,” *J. Dent.*, vol. 36, pp. 15–20, 2008.

[90] A. G. Wee, D. T. Lindsey, S. Kuo, and W. M. Johnston, “Color accuracy of commercial digital cameras for use in dentistry,” *Dent. Mater.*, vol. 22, no. 6, pp. 553–559, 2006.

[91] F. D. Jarad, M. D. Russell, and B. W. Moss, “The use of digital imaging for colour matching and communication in restorative dentistry,” *Br. Dent. J.*, vol. 199, no. 1, pp. 43–49, 2005.

[92] E. Cal, M. Sonugelen, P. Guneri, A. Kesercioglu, and T. Kose, “Application of a digital technique in evaluating the reliability of shade guides,” *J. Oral Rehabil.*, vol. 31, no. 5, pp. 483–491, 2004.

[93] C. V. V. B. de Almeida *et al.*, “Digital photography vs. clinical assessment of resin composite restorations,” *Odontology*, vol. 109, no. 1, pp. 184–192, 2021.

[94] S. A. Garg and S. M. Chavda, “Color masking white fluorotic spots by resin infiltration and its quantitation by computerized photographic analysis: a 12-month follow-up study,” *Oper. Dent.*, vol. 45, no. 1, pp. 1–9, 2020.

[95] D. Kim, S.-Y. Ahn, J. Kim, and S.-H. Park, “Interrater and intrarater reliability of FDI criteria applied to photographs of posterior tooth-colored restorations,” *J. Prosthet. Dent.*, vol. 118, no. 1, pp. 18–25, 2017.

[96] G. B. Rauber, J. K. Bernardon, L. C. C. Vieira, and L. N. Baratieri, “Evaluation of a technique for color correction in restoring anterior teeth,” *J. Esthet. Restor. Dent.*, vol. 29, no. 5, pp. 309–316, 2017.

[97] B. Irawan, S. M. Masudi, H. X. Han, N. F. Othman, and M. K. Alam, “In-Vitro Computer Analysis of Digital Images on Tooth-Colored Restorative Material Using L* a* b* Values,” *Int. Med. J.*, vol. 20, no. 5, pp. 623–625, 2013.

[98] F. Takatsui, M. F. de Andrade, M. P. Neisser, L. A. B. Barros, and L. de C. M. Loffredo, “CIE L* a* b*: comparison of digital images obtained photographically by manual and automatic modes,” *Braz. Oral Res.*, vol. 26, no. 6, pp. 578–583, 2012.

[99] A. E. Athanasiou, “Sensitivity of digital dental photo CIE L* a* b* analysis compared to spectrophotometer clinical assessments over 6 months,” *J Dent*, vol. 24, pp. 300–304, 2011.

[100] K. Yamanel, A. Caglar, M. Oezcan, K. Gulsah, and B. Bagis, “Assessment of color parameters of composite resin shade guides using digital imaging versus colorimeter,” *J. Esthet. Restor. Dent.*, vol. 22, no. 6, pp. 379–388, 2010.

[101] M. Kuzekanani and L. J. Walsh, “Quantitative analysis of KTP laser photodynamic bleaching of tetracycline-discolored teeth,” *Photomed. Laser Surg.*, vol. 27, no. 3, pp. 521–525, 2009.

[102] F. D. Jarad, S. S. Albadri, and L. H. Mair, “The use of objective digital matching to achieve an esthetic composite restoration.,” *J. Clin. Dent.*, vol. 19, no. 1, pp. 9–13, 2008.

[103] K.-Y. Lee, D. J. Setchell, A. N. Stokes, N. T. Frankel, and D. R. Moles, “Subjective

and photometric determination of bleaching outcomes.," *Quintessence Int. (Berl).*, vol. 38, no. 1, 2007.

[104] K. Gadhia, R. Shah, D. Swaminathan, S. Wetton, and J. Moran, "Development of a stain shade guide to aid the measurement of extrinsic dental stain," *Int. J. Dent. Hyg.*, vol. 4, no. 2, pp. 98–103, 2006.

[105] D. L. Lath, C. Johnson, R. N. Smith, and A. H. Brook, "Measurement of stain removal in vitro: a comparison of two instrumental methods," *Int. J. Dent. Hyg.*, vol. 4, no. 3, pp. 129–132, 2006.

[106] Y. H. Guan, D. L. Lath, T. H. Lilley, D. R. Willmot, I. Marlow, and A. H. Brook, "The measurement of tooth whiteness by image analysis and spectrophotometry: a comparison," *J. Oral Rehabil.*, vol. 32, no. 1, pp. 7–15, 2005.

[107] T. Tsuzuki *et al.*, "Evaluation of intraoral CCD camera for dental examination in forensic inspection," *Leg. Med.*, vol. 4, no. 1, pp. 40–46, 2002.

[108] C. Bentley, R. H. LEONARD, C. F. NELSON, and S. A. BENTLEY, "Quantitation of vital bleaching by computer analysis of photographic images," *J. Am. Dent. Assoc.*, vol. 130, no. 6, pp. 809–816, 1999.

[109] Y. Yousof, N. M. Salleh, and F. Yusof, "Quantitative Evaluation of Masticatory Performance with Two-Color Mixing Ability Test: Development of a New Digital Method.," *Int. J. Prosthodont.*, vol. 33, no. 2, 2020.

[110] P. Sam, V. Suresh, and D. Ganapathy, "Evaluation of variation in shade in metal-ceramic restoration from the shade tab.," *Drug Invent. Today*, vol. 11, no. 9, 2019.

[111] S. A. Sadek, W. M. Dehis, and H. Hassan, "Different Materials Used as Denture Retainers and Their Colour Stability," *Open access Maced. J. Med. Sci.*, vol. 6, no. 11, p. 2173, 2018.

[112] B.-R. Kim, S.-M. Kang, G.-M. Kim, and B.-I. Kim, "Differences in the intensity of light-induced fluorescence emitted by resin composites," *Photodiagnosis Photodyn. Ther.*, vol. 13, pp. 114–119, 2016.

[113] S. Lakhanpal and M. S. Neelima, "Accuracy of Three Shade-matching Devices in Replicating the Shade of Metal Ceramic Restorations: An in vitro Study.," *J. Contemp. Dent. Pract.*, vol. 17, no. 12, pp. 1003–1008, 2016.

[114] A. Van Der BILT, C. M. Speksnijder, R. de Liz Pocztaruk, and J. H. Abbink, "Digital image processing versus visual assessment of chewed two-colour wax in mixing ability tests," *J. Oral Rehabil.*, vol. 39, no. 1, pp. 11–17, 2012.

[115] F. Vafaii, F. Soltani, M. Kadkhodazadeh, Z. Moshiri, and M. Khoshhal, "Evaluation of color and contour matching accuracy with digital photography and direct vision," *Res. J. Med. Sci.*, vol. 6, no. 2, pp. 46–50, 2012.

[116] W. Oh, J. Pogoncheff, and W. J. O'Brien, "Digital computer matching of tooth color," *Materials (Basel).*, vol. 3, no. 6, pp. 3694–3699, 2010.

[117] E. CAL, P. Güneri, and T. Kose, "Digital analysis of mouthrinses' staining characteristics on provisional acrylic resins," *J. Oral Rehabil.*, vol. 34, no. 4, pp. 297–303, 2007.

[118] W. T. Wozniak, T. P. Muller, R. Silverman, and J. B. Moser, "Photographic assessment of colour changes in cold and heat-cure resins," *J. Oral Rehabil.*, vol. 8, no. 4, pp. 333–339, 1981.

[119] G. A. McCarty Jr, "Intraoral infrared color photography of radiotherapy patients," *J. Prosthet. Dent.*, vol. 35, no. 3, pp. 327–331, 1976.

[120] D. C. Mulcare and T. J. Coward, "Suitability of a mobile phone colorimeter application for use as an objective aid when matching skin color during the fabrication of a maxillofacial prosthesis," *J. Prosthodont.*, vol. 28, no. 8, pp. 934–943, 2019.

[121] P. Lagouvardo, N. Spyropoulou, and G. Polyzois, "Perceptibility and acceptability thresholds of simulated facial skin color differences," *J. Prosthodont. Res.*, vol. 62, no. 4, pp. 503–508, 2018.

[122] A. Sohaib, K. Amano, K. Xiao, J. M. Yates, C. Whitford, and S. Wuerger, "Colour quality of facial prostheses in additive manufacturing," *Int. J. Adv. Manuf. Technol.*, vol. 96, no. 1–4, pp. 881–894, 2018.

[123] M. Pricop, H. Balescu, S. Rosu, H. Urechescu, and Z. Crainiceanu, "Original Alternative Technique of Iris Fabrication for Ocular Prostheses," *Mater. Plast.*, vol. 53, no. 1, pp. 79–81, 2016.

[124] G. T. Grant, C. Aita-Holmes, P. Liacouras, J. Garnes, and W. O. Wilson Jr, "Digital capture, design, and manufacturing of a facial prosthesis: Clinical report on a pediatric

patient," *J. Prosthet. Dent.*, vol. 114, no. 1, pp. 138–141, 2015.

[125] M. M. Buzayan, Y. T. Ariffin, N. Yunus, and W. A. A. B. Mahmood, "Ocular defect rehabilitation using photography and digital imaging: A Clinical Report," *J. Prosthodont.*, vol. 24, no. 6, pp. 506–510, 2015.

[126] K. Xiao, F. Zardawi, R. van Noort, and J. M. Yates, "Developing a 3D colour image reproduction system for additive manufacturing of facial prostheses," *Int. J. Adv. Manuf. Technol.*, vol. 70, no. 9–12, pp. 2043–2049, 2014.

[127] K. W. Tan and I. D. Stephen, "Colour detection thresholds in faces and colour patches," *Perception*, vol. 42, no. 7, pp. 733–741, 2013.

[128] K. Xiao, F. Zardawi, R. van Noort, and J. M. Yates, "Color reproduction for advanced manufacture of soft tissue prostheses," *J. Dent.*, vol. 41, pp. e15–e23, 2013.

[129] S. H. Seo, J. H. Kim, J. W. Kim, Y. C. Kye, and H. H. Ahn, "Better understanding of digital photography for skin color measurement: With a special emphasis on light characteristics," *Ski. Res. Technol.*, vol. 17, no. 1, pp. 20–25, 2011.

[130] A. Delalleau, J.-M. Lagarde, and J. George, "An a priori shading correction technique for contact imaging devices," *IEEE Trans. image Process.*, vol. 20, no. 10, pp. 2876–2885, 2011.

[131] S. Jain, S. Makkar, S. Gupta, and A. Bhargava, "Prosthetic rehabilitation of ocular defect using digital photography: a case report," *J. Indian Prosthodont. Soc.*, vol. 10, no. 3, pp. 190–193, 2010.

[132] J. Pladellorens *et al.*, "A device for the color measurement and detection of spots on the skin," *Ski. Res. Technol.*, vol. 14, no. 1, pp. 65–70, 2008.

[133] T. Wang, J.-J. Bu, and C. Chen, "A color based face detection system using multiple templates," *J. Zhejiang Univ. A*, vol. 4, no. 2, pp. 162–165, 2003.

[134] M. Ghanoum, A. M. Ali, S. Elshazly, I. Alkabbany, and A. A. Farag, "Frame stitching in human oral cavity environment using intraoral camera," in *2019 IEEE International Conference on Image Processing (ICIP)*, IEEE, 2019, pp. 1327–1331.

[135] Y. Mayer, O. Ginesin, and E. E. Machtei, "Photometric CIELAB analysis of the gingiva: a novel approach to assess response to periodontal therapy," *J. Periodontol.*,

vol. 88, no. 9, pp. 854–859, 2017.

[136] E. D. Berdouses, G. D. Koutsouri, E. E. Tripoliti, G. K. Matsopoulos, C. J. Oulis, and D. I. Fotiadis, "A computer-aided automated methodology for the detection and classification of occlusal caries from photographic color images," *Comput. Biol. Med.*, vol. 62, pp. 119–135, 2015.

[137] L. Torlakovic, I. Olsen, C. Petzold, H. Tiainen, and B. Øgaard, "Clinical color intensity of white spot lesions might be a better predictor of enamel demineralization depth than traditional clinical grading," *Am. J. Orthod. Dentofac. Orthop.*, vol. 142, no. 2, pp. 191–198, 2012.

[138] Y. Iwami, N. Hayashi, H. Yamamoto, M. Hayashi, F. Takeshige, and S. Ebisu, "Evaluating the objectivity of caries removal with a caries detector dye using color evaluation and PCR," *J. Dent.*, vol. 35, no. 9, pp. 749–754, 2007.

[139] P. Berssenbrügge *et al.*, "Measuring facial symmetry: a perception-based approach using 3D shape and color," *Biomed. Eng. Tech.*, vol. 60, no. 1, pp. 39–47, 2015.

[140] Y. S. N. Jayaratne, C. P. J. McGrath, and R. A. Zwahlen, "How accurate are the fusion of cone-beam CT and 3-D stereophotographic images?," *PLoS One*, vol. 7, no. 11, p. e49585, 2012.

[141] Y. S. N. Jayaratne, R. A. Zwahlen, J. Lo, and L. K. Cheung, "Three-dimensional color maps: a novel tool for assessing craniofacial changes," *Surg. Innov.*, vol. 17, no. 3, pp. 198–205, 2010.

[142] E. Brough, A. N. Donaldson, and F. B. Naini, "Canine substitution for missing maxillary lateral incisors: the influence of canine morphology, size, and shade on perceptions of smile attractiveness," *Am. J. Orthod. Dentofac. Orthop.*, vol. 138, no. 6, pp. 705-e1, 2010.
