# Supplementary material for: Digital Shade Matching in Dentistry: A Systematic Review
Source: Dent J (Basel). 2023 Oct 27;11(11):250. doi: 10.3390/dj11110250 (PMC10670912; doi:10.3390/dj11110250)
Supplement: Supplementary file 1 [file dentistry-11-00250-s001.zip › File S2 Table S14 and S15.pdf]

Table S14

Quality of study analysis using
Cochrane GRADEpro

Colorimetry in Dentistry

Question: Digital photo compared to conventional technique in color sciences

Certainty assessment							№ of patients		Effect		Certainty	Importance
**№ of studies**	**Study design**	**Risk of bias**	**Inconsistency**	**Indirectness**	**Imprecision**	**Other considerations**	**Digital photo**	**conventional technique**	**Relative (95% CI)**	**Absolute (95% CI)**		
**Color assessment in dental prostheses: the use of smartphones as process tools**												
1	observational studies	not serious	not serious	not serious	not serious	all plausible residual confounding would reduce the demonstrated effect		-	-	-	⊕⊕⊕◯ MODERATE	
**Application of a digital technique in evaluating the reliability of shade guides**												
1	observational studies	not serious	not serious	not serious	serious [a]	all plausible residual confounding would reduce the demonstrated effect			not estimable		⊕⊕◯◯ LOW	

Certainty assessment							№ of patients		Effect		Certainty	Importance
№ of studies	Study design	Risk of bias	Inconsistency	Indirectness	Imprecision	Other considerations	Digital photo	conventional technique	Relative (95% CI)	Absolute (95% CI)		
**In vitro evaluation of new dental software for shade matching**												
1	observational studies	serious [b]	not serious	not serious	serious [c]	strong association all plausible residual confounding would reduce the demonstrated effect			not estimable		⊕⊕◯◯ LOW	
**A novel regression model from RGB image data to spectroradiometric correlates optimized for tooth colored shades**												
1	observational studies	not serious	not serious	not serious	not serious	strong association			not estimable		⊕⊕⊕◯ MODERATE	
**Photometric assessment of tooth color using commonly available software**												
1	observational studies	serious [d]	not serious	not serious	not serious	strong association			not estimable		⊕⊕◯◯ LOW	

Certainty assessment							№ of patients		Effect		Certainty	Importance
**№ of studies**	**Study design**	**Risk of bias**	**Inconsistency**	**Indirectness**	**Imprecision**	**Other considerations**	**Digital photo**	**conventional technique**	**Relative (95% CI)**	**Absolute (95% CI)**		
**The relationship of tooth shade and skin tone and its influence on the smile attractiveness**												
1	observational studies	serious [e]	not serious	not serious	not serious	strong association all plausible residual confounding would reduce the demonstrated effect			not estimable		⊕⊕⊕◯ MODERATE	
**Evaluation of dental shade guide variability using cross-polarized photography**												
1	observational studies	serious [f]	not serious	not serious	not serious	none			not estimable	-	⊕◯◯◯ VERY LOW	

CI: Confidence interval

Explanations

a. • Daylight regulations were not described • use of 74-pixel space was not explained.

b. • The reference shade tab was conveniently selected

c. • Environmental factor control methods were not adequately described

d. • Only measured the color of the premolar tooth

e. • The study was conducted within the same ethnic group • There were multiple uncategorized variations within the shade guide

f. • Shade tabs were conveniently selected

Question: Digital photo compared to conventional technique in color sciences

Certainty assessment							№ of patients		Effect		Certainty	Importance
**№ of studies**	**Study design**	**Risk of bias**	**Inconsistency**	**Indirectness**	**Imprecision**	**Other considerations**	**Digital photo**	**conventional technique**	**Relative (95% CI)**	**Absolute (95% CI)**		
**Evaluating the relationship between tooth color and enamel thickness, using twin flash photography, cross-polarization photography, and spectrophotometer**												
1	observational studies	not serious	not serious	not serious	not serious	strong association			not estimable		⨁⨁⨁◯ MODERATE	
**The use of a standardized gray reference card on dental photography to correct the effects of five commonly used diffusers on the color of 40 extracted human tooth**												
1	observational studies	serious [a]	not serious	not serious	not serious	strong association			not estimable		⨁⨁◯◯ LOW	
**The use of digital imaging for colour matching and communication in restorative dentistry**												

Certainty assessment							№ of patients		Effect		Certainty	Importance
№ of studies	Study design	Risk of bias	Inconsistency	Indirectness	Imprecision	Other considerations	Digital photo	conventional technique	Relative (95% CI)	Absolute (95% CI)		
1	observational studies	serious [b]	not serious	not serious	not serious	strong association			not estimable		⊕⊕◯◯ LOW	
**The accuracy of dental shade matching using cross-polarization photography**												
1	observational studies	not serious	not serious	not serious	not serious	strong association			not estimable		⊕⊕⊕◯ MODERATE	
**Assessment of the influence of gender and skin color on the preference of tooth shade in Saudi population**												
1	observational studies	serious [c]	not serious	not serious	serious [d]	strong association all plausible residual confounding would reduce the demonstrated effect			not estimable		⊕⊕◯◯ LOW	
**Mapping intraoral photographs on virtual teeth model**												

Certainty assessment							№ of patients		Effect		Certainty	Importance
№ of studies	Study design	Risk of bias	Inconsistency	Indirectness	Imprecision	Other considerations	Digital photo	conventional technique	Relative (95% CI)	Absolute (95% CI)		
1	observational studies	not serious	not serious	not serious	not serious	strong association			not estimable		⨁⨁⨁◯ MODERATE	
**Comparison of visual analog shade matching, a digital visual method with a cross-polarized light filter, and a spectrophotometer for dental color matching**												
1	observational studies	not serious	not serious	not serious	not serious	strong association			not estimable		⨁⨁⨁◯ MODERATE	

CI: Confidence interval

Explanations

a. • The sample characteristics were not clearly described

b. • The shade tabs were conveniently selected • The observers had varying experience in restorative dentistry

c. • Teeth shades were conveniently selected • Focused on only one ethnic group • Different lighting conditions and background were used while taking the photographs

d. • Different lighting conditions and background were used while taking the photographs

Question: Digital photo compared to conventional technique in color sciences

Certainty assessment							№ of patients		Effect		Certainty	Importance
№ of studies	Study design	Risk of bias	Inconsistency	Indirectness	Imprecision	Other considerations	Digital photo	conventional technique	Relative (95% CI)	Absolute (95% CI)		
**Comparison of shade matching photos taken with gray card and without gray card**												
1	observational studies	serious [a]	not serious	not serious	serious [b]	all plausible residual confounding would reduce the demonstrated effect			not estimable		⨁◯◯◯ VERY LOW	
**The in vitro and in vivo reproducibility of a video-based digital imaging system for tooth colour measurement**												
1	observational studies	serious [c]	not serious	not serious	not serious	strong association			not estimable		⨁⨁◯◯ LOW	
**Assessing Tooth Color Differences in Digital Facial Portraits**												
1	observational studies	serious [d]	not serious	not serious	not serious	strong association			not estimable		⨁⨁◯◯ LOW	

Certainty assessment							№ of patients		Effect		Certainty	Importance
№ of studies	Study design	Risk of bias	Inconsistency	Indirectness	Imprecision	Other considerations	Digital photo	conventional technique	Relative (95% CI)	Absolute (95% CI)		
**In-vivo tooth color-measurement with a new 3D intraoral scanning system in comparison to conventional digital and visual color determination method**												
1	observational studies	serious [e]	not serious	not serious	not serious	strong association all plausible residual confounding would reduce the demonstrated effect			not estimable		⨁⨁⨁◯ MODERATE	
**Comparison of photographic and conventional methods for tooth shade selection: A clinical evaluation**												
1	observational studies	serious [f]	not serious	not serious	not serious	strong association all plausible residual confounding would reduce the demonstrated effect			not estimable		⨁⨁⨁◯ MODERATE	

Certainty assessment							№ of patients		Effect		Certainty	Importance
№ of studies	Study design	Risk of bias	Inconsistency	Indirectness	Imprecision	Other considerations	Digital photo	conventional technique	Relative (95% CI)	Absolute (95% CI)		
**Contributions of dental colour to the physical attractiveness stereotype**												
1	observational studies	serious [g]	not serious	not serious	not serious	none			not estimable		⊕◯◯◯ VERY LOW	
**Variability of color matching with different digital photography techniques and a reference card**												
1	observational studies	not serious	not serious	not serious	not serious	strong association			not estimable		⊕⊕⊕◯ MODERATE	

CI: Confidence interval

Explanations

a. • Incomplete description of camera settings were provided

b. • Incomplete description of camera settings were provided • Statistical analyses and values were not provided

c. • The shade guide was conveniently selected • Incomplete camera description was provided • For in vivo analysis only central incisor was used as sample

d. • The study was conducted between 2 specific ethnic groups

e. • The teeth were conveniently selected for color analysis

f. • Only the middle third of the maxillary right central incisor was evaluated • Daylight regulations were not described

g. • Full description of the camera setup was not provided • Ethnic variations were not considered • Dental student observers were used that may not represent the general population

Question: Digital photo compared to conventional technique in color sciences

Certainty assessment							№ of patients		Effect		Certainty	Importance
№ of studies	Study design	Risk of bias	Inconsistency	Indirectness	Imprecision	Other considerations	Digital photo	conventional technique	Relative (95% CI)	Absolute (95% CI)		
**Dental shade guide variability for hues B, C and D using cross- polarized photography**												
1	observational studies	not serious	not serious	not serious	not serious	none			not estimable		⨁⨁◯◯ LOW	
**Shade Matching Assisted by Digital Photography and Computer Software**												
1	observational studies	not serious	not serious	not serious	not serious	none			not estimable		⨁⨁◯◯ LOW	
**The in vitro and in vivo of a mobile non-contact camera-based digital imaging system for tooth color measurement**												
1	observational studies	not serious	not serious	not serious	not serious	strong association			not estimable		⨁⨁⨁◯ MODERATE	

Certainty assessment							№ of patients		Effect		Certainty	Importance
№ of studies	Study design	Risk of bias	Inconsistency	Indirectness	Imprecision	Other considerations	Digital photo	conventional technique	Relative (95% CI)	Absolute (95% CI)		
**Dental shade matching using a digital camera**												
1	observational studies	serious [a]	not serious	not serious	not serious	all plausible residual confounding would reduce the demonstrated effect			not estimable		⊕⊕◯◯ LOW	
**Accurate shade image matching by using a smartphone camera**												
1	observational studies	serious [b]	not serious	not serious	not serious	strong association all plausible residual confounding would reduce the demonstrated effect			not estimable		⊕⊕⊕◯ MODERATE	
**Development of digital shade guides for color assessment using a digital camera with ring flashes**												

Certainty assessment							№ of patients		Effect		Certainty	Importance
№ of studies	Study design	Risk of bias	Inconsistency	Indirectness	Imprecision	Other considerations	Digital photo	conventional technique	Relative (95% CI)	Absolute (95% CI)		
1	observational studies	not serious	not serious	not serious	not serious	strong association			not estimable		⊕⊕⊕◯ MODERATE	
**A Study on Possibility of Clinical Application for Color Measurements of Shade Guides Using an Intraoral Digital Scanner**												
1	observational studies	serious [c]	not serious	not serious	not serious	strong association			not estimable		⊕⊕◯◯ LOW	

CI: Confidence interval

Explanations

a. • The shade tab was conveniently selected

b. • A color temperature meter would be needed for doing SVM color analysis because a constant illumination is required

c. • The shade tabs were conveniently selected and only includes 5 tabs • The measurements were taken by a single observer

Question: Digital photo compared to conventional technique in color sciences

Certainty assessment							№ of patients		Effect		Certainty	Importance
№ of studies	Study design	Risk of bias	Inconsistency	Indirectness	Imprecision	Other considerations	Digital photo	conventional technique	Relative (95% CI)	Absolute (95% CI)		
**The development of a 3D color reproduction system of digital impressions with an intraoral scanner and a 3D printer: a preliminary study**												
1	observational studies	not serious	not serious	not serious	not serious	strong association all plausible residual confounding would reduce the demonstrated effect			not estimable		⨁⨁⨁⨁ HIGH	

Color accuracy of commercial digital cameras for use in dentistry

1	observational studies	serious [a]	not serious	not serious	not serious	strong association all plausible residual confounding would reduce the demonstrated effect			not estimable		⊕⊕⊕◯ MODERATE	

A Digital Shade-Matching Device for Dental Color Determination Using the Support Vector Machine Algorithm

1	observational studies	not serious	not serious	not serious	not serious	strong association			not estimable		⊕⊕⊕◯ MODERATE	

Comparative Analysis of Color Matching System for Teeth Recognition Using Color Moment

1	observational studies	serious [b]	not serious	not serious	not serious	strong association			not estimable		⊕⊕◯◯ LOW	

An efficient image processing methodology based on fuzzy decision for dental shade matching

1	observational studies	not serious	not serious	not serious	not serious	all plausible residual confounding would reduce the demonstrated effect			not estimable		⊕⊕⊕◯ MODERATE	

Could digital imaging be an alternative for digital colorimeters?

1	observational studies	not serious	not serious	not serious	not serious	all plausible residual confounding would reduce the demonstrated effect			not estimable		⨁⨁⨁◯ MODERATE	

A comparison between a new visual method of color matching by intraoral camera and conventional visual and spectrometric methods

1	observational studies	not serious	not serious	not serious	not serious	strong association all plausible residual confounding would reduce the demonstrated effect			not estimable		⨁⨁⨁⨁ HIGH	

CI: Confidence interval

Explanations

a. • The cameras, shade tabs and color patches were conveniently selected

b. • Racial and geographic dental shade variations were not considered during the design

Endodontics and Dental Esthetics

Question: Digital photo compared to conventional technique in endodontics and restorative dentistry

Certainty assessment							№ of patients		Effect		Certainty	Importance
№ of studies	Study design	Risk of bias	Inconsistency	Indirectness	Imprecision	Other considerations	Digital photo	conventional technique	Relative (95% CI)	Absolute (95% CI)		
**Digital photography vs. clinical assessment of resin composite restorations**												
1	observational studies	serious [a]	not serious	not serious	not serious	strong association all plausible residual confounding would reduce the demonstrated effect			not estimable		⊕⊕⊕◯ MODERATE	
**Color Masking White Fluorotic Spots by Resin Infiltration and Its Quantitation by Computerized Photographic Analysis: A 12-month Follow-up Study**												
1	observational studies	not serious	not serious	not serious	not serious	strong association			not estimable		⊕⊕⊕◯ MODERATE	

Certainty assessment							№ of patients		Effect		Certainty	Importance
№ of studies	Study design	Risk of bias	Inconsistency	Indirectness	Imprecision	Other considerations	Digital photo	conventional technique	Relative (95% CI)	Absolute (95% CI)		
**Interrater and intrarater reliability of FDI criteria applied to photographs of posterior tooth-colored restorations**												
1	observational studies	serious [b]	not serious	not serious	not serious	strong association			not estimable		⊕⊕◯◯ LOW	
**Evaluation of a technique for color correction in restoring anterior teeth**												
1	observational studies	not serious	not serious	not serious	not serious	strong association all plausible residual confounding would reduce the demonstrated effect			not estimable		⊕⊕⊕⊕ HIGH	
**In-Vitro Computer Analysis of Digital Images on ToothColored Restorative Material Using L*a*b* Values**												
1	observational studies	not serious	not serious	not serious	not serious	strong association			not estimable		⊕⊕⊕◯ MODERATE	

Certainty assessment							№ of patients		Effect		Certainty	Importance
№ of studies	Study design	Risk of bias	Inconsistency	Indirectness	Imprecision	Other considerations	Digital photo	conventional technique	Relative (95% CI)	Absolute (95% CI)		
**CIE L*a*b*: comparison of digital images obtained photographically by manual and automatic modes**												
1	observational studies	serious [c]	not serious	not serious	not serious	strong association all plausible residual confounding would reduce the demonstrated effect			not estimable		⊕⊕⊕◯ MODERATE	
**Sensitivity of digital dental photo CIE L*a*b* analysis compared to spectrophotometer clinical assessments over 6 months**												
1	observational studies	not serious	not serious	not serious	not serious	none			not estimable		⊕⊕◯◯ LOW	

CI: Confidence interval

Explanations

a. • Incomplete description of environment was provided • Inter-observer reliability was not performed

b. • Only the occlusal restorations were evaluated • Only 2 of the 5 raters were conveniently selected for re-evaluation

c. • Only one camera and brand were evaluated

Question: Digital photo compared to conventional technique in endodontics and restorative dentistry

Certainty assessment							№ of patients		Effect		Certainty	Importance
№ of studies	Study design	Risk of bias	Inconsistency	Indirectness	Imprecision	Other considerations	Digital photo	conventional technique	Relative (95% CI)	Absolute (95% CI)		
**Assessment of Color Parameters of Composite Resin Shade Guides Using Digital Imaging versus Colorimeterjerd_370**												
1	observational studies	serious [a]	not serious	not serious	not serious	all plausible residual confounding would reduce the demonstrated effect			not estimable		⨁⨁◯◯ LOW	
**Quantitative Analysis of KTP Laser Photodynamic Bleaching of Tetracycline-Discolored Teeth**												
1	observational studies	not serious	not serious	not serious	not serious	strong association			not estimable		⨁⨁⨁◯ MODERATE	

**The Use of Objective Digital Matching to Achieve Aesthetic Composite Restoration**												
1	observational studies	serious [b]	not serious	not serious	not serious	strong association all plausible residual confounding would reduce the demonstrated effect			not estimable		⊕⊕⊕◯ MODERATE	
**Subjective and photometric determination of bleaching outcomes**												
1	observational studies	not serious	not serious	not serious	not serious	strong association			not estimable		⊕⊕⊕◯ MODERATE	
**Development of a stain shade guide to aid the measurement of extrinsic dental stain**												
1	observational studies	serious [c]	not serious	not serious	not serious	strong association all plausible residual confounding would reduce the demonstrated effect			not estimable		⊕⊕⊕◯ MODERATE	

Measurement of stain removal in vitro: a comparison of two instrumental methods

1	observational studies	not serious	not serious	not serious	not serious	strong association all plausible residual confounding would reduce the demonstrated effect			not estimable		⊕⊕⊕⊕ HIGH	

The measurement of tooth whiteness by image analysis and spectrophotometry: a comparison

1	observational studies	not serious	not serious	not serious	not serious	all plausible residual confounding would reduce the demonstrated effect			not estimable		⊕⊕⊕◯ MODERATE	

CI: Confidence interval

Explanations

a. • Shade tabs were conveniently selected

b. • The color chips, composite materials were conveniently selected • Only central incisor was used • Experts from dentistry were selected as observers

c. • Acrylic shades were conveniently selected • The study relied heavily on staining by caffeine-based tea solution

Question: Digital photo compared to conventional technique in endodontics and restorative dentistry

Certainty assessment							№ of patients		Effect		Certainty	Importance
**№ of studies**	**Study design**	**Risk of bias**	**Inconsistency**	**Indirectness**	**Imprecision**	**Other considerations**	**Digital photo**	**conventional technique**	**Relative (95% CI)**	**Absolute (95% CI)**		
**Evaluation of intraoral CCD camera for dental examination in forensic inspection**												
1	observational studies	not serious	not serious	not serious	not serious	none			not estimable		⊕⊕◯◯ LOW	
**Quantification of vital bleaching by computer analysis of photographic images**												
1	observational studies	serious [a]	not serious	not serious	not serious	all plausible residual confounding would reduce the demonstrated effect			not estimable		⊕⊕◯◯ LOW	

CI: Confidence interval

Explanations

a. • Participants were conveniently selected

Maxillofacial Prostheses

Question: Digital photo compared to conventional technique in maxillofacial prosthesis

Certainty assessment							№ of patients		Effect		Certainty	Importance
№ of studies	Study design	Risk of bias	Inconsistency	Indirectness	Imprecision	Other considerations	Digital photo	conventional technique	Relative (95% CI)	Absolute (95% CI)		
**Perceptibility and acceptability thresholds of simulated facial skin color differences**												
1	observational studies	serious [a]	not serious	not serious	not serious	strong association all plausible residual confounding would reduce the demonstrated effect			not estimable		⨁⨁⨁◯ MODERATE	IMPORTANT

Suitability of a Mobile Phone Colorimeter Application for Use as an Objective Aid when Matching Skin Color during the Fabrication of a Maxillofacial Prosthesis

1	observational studies	serious [b]	not serious	not serious	not serious	strong association all plausible residual confounding would reduce the demonstrated effect			not estimable		⨁⨁⨁◯ MODERATE	

Original Alternative Technique of Iris Fabrication for Ocular Prostheses

1	observational studies	not serious	not serious	not serious	not serious	none		-	-	-	⨁⨁◯◯ LOW	

Better understanding of digital photography for skin color measurement: With a special emphasis on light characteristics

1	observational studies	serious [c]	not serious	not serious	not serious	strong association all plausible residual confounding would reduce the demonstrated effect			not estimable		⨁⨁⨁◯ MODERATE	

Colour quality of facial prostheses in additive manufacturing

1	observational studies	not serious	not serious	not serious	not serious	none			not estimable		⊕⊕◯◯ LOW	

Colour detection thresholds in faces and colour patches

1	observational studies	not serious	not serious	not serious	not serious	strong association all plausible residual confounding would reduce the demonstrated effect			not estimable	-	⊕⊕⊕⊕ HIGH	

A color based face detection system using multiple templates

1	observational studies	serious [d]	not serious	not serious	serious [e]	all plausible residual confounding would reduce the demonstrated effect			not estimable		⊕◯◯◯ VERY LOW	

CI: Confidence interval

Explanations

a. Different age groups and genders have varying tolerance to colour changes. Such changes were not controlled in the study

b. An unclear description of the custom-made jig The smartphone used, silicone material and pigments used were conveniently selected

c. The study design cannot be reproduced for dental prostheses

d. The ethnic groups were conveniently selected for the study

e. RGB colour space is not reliable for accurate facial skin tone identification.

Question: Digital photo compared to conventional technique in maxillofacial prosthesis

Certainty assessment							№ of patients		Effect		Certainty	Importance
**№ of studies**	**Study design**	**Risk of bias**	**Inconsistency**	**Indirectness**	**Imprecision**	**Other considerations**	**Digital photo**	**conventional technique**	**Relative (95% CI)**	**Absolute (95% CI)**		
**Developing a 3D colour image reproduction system for additive manufacturing of facial prostheses**												
1	observational studies	not serious	not serious	not serious	serious [a]	strong association			not estimable		⨁⨁◯◯ LOW	

Color reproduction for advanced manufacture of soft tissue prostheses

1	observational studies	not serious	not serious	not serious	serious [b]	none			not estimable		⊕◯◯◯ VERY LOW	

An a priori Shading Correction Technique for Contact Imaging Devices

1	observational studies	serious [b]	not serious	not serious	serious [c]	none			not estimable		⊕◯◯◯ VERY LOW	

A device for the color measurement and detection of spots on the skin

1	observational studies	serious [d]	not serious	not serious	not serious	all plausible residual confounding would reduce the demonstrated effect			not estimable		⊕⊕◯◯ LOW	

Ocular Defect Rehabilitation Using Photography and Digital Imaging: A Clinical Report

1	observational studies	serious [e]	not serious	not serious	not serious	strong association		-	-	-	⊕⊕◯◯ LOW	

Digital capture, design, and manufacturing of a facial prosthesis: Clinical report on a pediatric patient

1	observational studies	serious [f]	not serious	not serious	serious [g]	none		-	-	-	⊕◯◯◯ VERY LOW	

Prosthetic Rehabilitation of Ocular Defect Using Digital Photography: A Case Report

1	observational studies	not serious	not serious	not serious	not serious	all plausible residual confounding would reduce the demonstrated effect		-	-	-	⨁⨁⨁◯ MODERATE	

CI: Confidence interval

Explanations

a. Only two prostheses; one nose and one ear were validated with this proposed technique

b. For proper shading correction is based on adequate lighting condition and standardisation. The current study made no mention of light control.

c. The device accuracy was limited to an area of between 1 and 300mm2 due to lack of illuminance of the edges.

d. The samples were not properly defined. The device accuracy was limited to an area of between 1 and 300mm2 due to lack of illuminance of the edges.

e. Incomplete camera details were provided. Duplication of the iris depends upon quality of the printer ink, printer paper type and skills to use the software including digital photography technique

f. Shade mixing relied predominantly on conventional visual perception

g. Color matched with utilizing visual perception

Prosthodontics

Question: Digital photo compared to conventional technique in prosthodontics

Certainty assessment							№ of patients		Effect		Certainty	Importance
№ of studies	Study design	Risk of bias	Inconsistency	Indirectness	Imprecision	Other considerations	Digital photo	conventional technique	Relative (95% CI)	Absolute (95% CI)		
**Quantitative Evaluation of Masticatory Performance with Two-Color Mixing Ability Test: Development of a New Digital Method**												
1	observational studies	serious [a]	not serious	not serious	not serious	strong association			not estimable		⨁⨁◯◯ LOW	
**Evaluation of variation in shade in metal-ceramic restoration from the shade tab**												
1	observational studies	serious [b]	not serious	not serious	not serious	all plausible residual confounding would reduce the demonstrated effect			not estimable		⨁⨁◯◯ LOW	

Different Materials Used as Denture Retainers and Their Colour Stability

1	observational studies	not serious	not serious	not serious	not serious	none			not estimable		⨁⨁◯◯ LOW	

Differences in the intensity of light-induced fluorescence emitted by resin composites

1	observational studies	serious [c]	not serious	not serious	not serious	strong association			not estimable		⨁⨁◯◯ LOW	

Accuracy of Three Shade-matching Devices in Replicating the Shade of Metal Ceramic Restorations: An in vitro Study

1	observational studies	not serious	not serious	not serious	not serious	none			not estimable		⨁⨁◯◯ LOW	

Digital image processing versus visual assessment of chewed two-colour wax in mixing ability tests

1	observational studies	not serious	not serious	not serious	not serious	all plausible residual confounding would reduce the demonstrated effect			not estimable		⨁⨁⨁◯ MODERATE	

Digital analysis of mouth rinse staining characteristics on provisional acrylic resins

1	observational studies	not serious	not serious	not serious	not serious	strong association all plausible residual confounding would reduce the demonstrated effect			not estimable		⨁⨁⨁⨁ HIGH	

CI: Confidence interval

Explanations

a. The proposed method was not compared to any gold standard

b. •Glazed material photographed under natural light could introduce possible reflection-based errors during photo capture. •The results could have been influenced by the porcelain thickness and metal properties

c. •Tooth samples were conveniently selected •The blue lights present within the study contains blue lights may not be suitable for inducing highest fluorescent intensities. •The filter on QLF-D technology sometimes filters out extra wavelength resulting in greater fluorescence intensity

Question: Digital photo compared to conventional technique in prosthodontics

Certainty assessment							№ of patients		Effect		Certainty	Importance
№ of studies	Study design	Risk of bias	Inconsistency	Indirectness	Imprecision	Other considerations	Digital photo	conventional technique	Relative (95% CI)	Absolute (95% CI)		
**Photographic assessment of colour changes in cold and heat-cure resins**												
1	observational studies	serious [a]	not serious	not serious	not serious	all plausible residual confounding would reduce the demonstrated effect			not estimable		⊕⊕◯◯ LOW	
**Evaluation of color and contour matching accuracy with digital photography and direct vision**												
1	observational studies	serious [b]	not serious	not serious	serious [c]	all plausible residual confounding would reduce the demonstrated effect			not estimable		⊕◯◯◯ VERY LOW	

Digital Computer Matching of Tooth Color

1	observational studies	not serious	not serious	not serious	not serious	strong association			not estimable		⊕⊕⊕◯ MODERATE	

Intraoral infrared color photography of radiotherapy patients

1	observational studies	not serious	not serious	not serious	not serious	all plausible residual confounding would reduce the demonstrated effect		-	-	-	⊕⊕⊕◯ MODERATE	

CI: Confidence interval

Explanations

a. • Temperature control or variations were not specified

b. • The observers had varying experience in restorative dentistry • The parameters used as standard were unclear

c. • Incomplete camera details were provided • The lighting apparatus was not clearly defined

Periodontics and Oral Pathology

Question: Digital photo compared to conventional technique in periodontology

Certainty assessment							№ of patients		Effect		Certainty	Importance
№ of studies	Study design	Risk of bias	Inconsistency	Indirectness	Imprecision	Other considerations	Digital photo	conventional technique	Relative (95% CI)	Absolute (95% CI)		
**FRAME STITCHING IN HUMAN ORAL CAVITY ENVIRONMENT USING INTRAORAL CAMERA**												
1	observational studies	serious [a]	not serious	not serious	not serious	all plausible residual confounding would reduce the demonstrated effect			not estimable		⨁⨁◯◯ LOW	
**Photometric CIELAB Analysis of the Gingiva: A Novel Approach to Assess Response to Periodontal Therapy**												
1	observational studies	serious [b]	not serious	not serious	not serious	all plausible residual confounding would reduce the demonstrated effect			not estimable		⨁⨁◯◯ LOW	

CI: Confidence interval

Explanations

a. •SFS cannot always provide accurate information on the surface normal. •The study relied on software-based simulations of intraoral lighting conditions

b. •A single tooth was analysed which might not justly reflect the rest of the dentition •Only one race of samples was selected •White balance and colour calibration methods were not mentioned •Software used for color evaluation was not mentioned

Question: Digital photo compared to conventional technique in oral pathology

Certainty assessment							№ of patients		Effect		Certainty	Importance
№ of studies	Study design	Risk of bias	Inconsistency	Indirectness	Imprecision	Other considerations	Digital photo	conventional technique	Relative (95% CI)	Absolute (95% CI)		
**A computer-aided automated methodology for the detection and classification of occlusal caries from photographic color images**												
1	observational studies	serious [a]	not serious	not serious	not serious	strong association	68/103 (66.0%)		not estimable		⨁⨁◯◯ LOW	

**Clinical color intensity of white spot lesions might be a better predictor of enamel demineralization depth than traditional clinical grading**												
1	observational studies	serious [b]	not serious	not serious	not serious	strong association all plausible residual confounding would reduce the demonstrated effect			not estimable		⨁⨁⨁◯ MODERATE	
**Evaluating the objectivity of caries removal with a caries detector dye using color evaluation and PCR**												
1	observational studies	serious [c]	not serious	not serious	not serious	strong association all plausible residual confounding would reduce the demonstrated effect			not estimable		⨁⨁⨁◯ MODERATE	

CI: Confidence interval

Explanations

a. Different camera and setups were used for in-vivo and in-vitro photographs

b. The outcomes were influenced by patient dependent variables such as brushing habits, that were not controlled in the study

c. •only carious molar teeth were selected •The samples were not equally distributed among the operators •The samples were selected based on operators' visual judgement

Orthodontics

Question: Digital photo compared to conventional technique for orthodontics and Dentofacial orthopedics

Certainty assessment							№ of patients		Effect		Certainty	Importance
**№ of studies**	**Study design**	**Risk of bias**	**Inconsistency**	**Indirectness**	**Imprecision**	**Other considerations**	**Digital photo**	**conventional technique**	**Relative (95% CI)**	**Absolute (95% CI)**		
**Measuring facial symmetry: a perception-based approach using 3D shape and color**												
1	observational studies	serious [a]	not serious	not serious	not serious	strong association all plausible residual confounding would reduce the demonstrated effect			not estimable		⊕⊕⊕◯ MODERATE	
**Three-Dimensional Color Maps: A Novel Tool for Assessing Craniofacial Changes**												
1	observational studies	serious [b]	not serious	not serious	not serious	none		-	-	-	⊕◯◯◯ VERY LOW	

How Accurate Are the Fusion of Cone-Beam CT and 3-D Stereophotographic Images?

1	observational studies	not serious	not serious	not serious	not serious	strong association all plausible residual confounding would reduce the demonstrated effect			not estimable		⊕⊕⊕⊕ HIGH	

Canine substitution for missing maxillary lateral incisors: The influence of canine morphology, size, and shade on perceptions of smile attractiveness

1	observational studies	serious [c]	not serious	not serious	not serious	strong association			not estimable		⊕⊕◯◯ LOW	

CI: Confidence interval

Explanations

a. • Above hairline and ear were excluded by indicating as a complex surface to record the details • There has been a selective age range was given who used as an observer

b. • Author describe that majority of the software for color maps is not scientifically validated in clinical trials

c. • 120 observers were selected as participants including orthodontists, dentist and lay people • For image patients having hypodontia were selected

Table S15

Joanna Briggs Institute (JBI) critical appraisal results

Colorimetry in Dentistry

JBI CRITICAL APPRAISAL CHECKLIST FOR CASE REPORTS

Reviewer____________________ Date 1.11.2020

Author Albert et al Year 2019 Record Number 1

	Yes	No	Unclear	Not applicable
1. Were patient's demographic characteristics clearly described?	☐	☐	☑	☐
2. Was the patient's history clearly described and presented as a timeline?	☐	☐	☑	☐
3. Was the current clinical condition of the patient on presentation clearly described?	☐	☐	☑	☐
4. Were diagnostic tests or assessment methods and the results clearly described?	☐	☐	☑	☐
5. Was the intervention(s) or treatment procedure(s) clearly described?	☑	☐	☐	☐
6. Was the post-intervention clinical condition clearly described?	☐	☐	☑	☐
7. Were adverse events (harms) or unanticipated events identified and described?	☐	☑	☐	☐
8. Does the case report provide takeaway lessons?	☑	☐	☐	☐

Overall appraisal: Include ☑ Exclude ☐ Seek further info ☐

Comments (Including reason for exclusion)

© JBI, 2020. All rights reserved. JBI grants use of these tools for research purposes only. All other enquiries should be sent to jbisynthesis@adelaide.edu.au.

JBI CRITICAL APPRAISAL CHECKLIST FOR QUASI-EXPERIMENTAL STUDIES

Reviewer________________________ Date 11.11.2020

Author Culic et al. Year 2014 Record Number 2

	Yes	No	Unclear	Not applicable
1. Is it clear in the study what is the 'cause' and what is the 'effect' (i.e. there is no confusion about which variable comes first)?	☑	☐	☐	☐
2. Were the participants included in any comparisons similar?	☑	☐	☐	☐
3. Were the participants included in any comparisons receiving similar treatment/care, other than the exposure or intervention of interest?	☑	☐	☐	☐
4. Was there a control group?	☐	☑	☐	☐
5. Were there multiple measurements of the outcome both pre and post the intervention/exposure?	☐	☑	☐	☐
6. Was follow up complete and if not, were differences between groups in terms of their follow up adequately described and analyzed?	☐	☐	☐	☑
7. Were the outcomes of participants included in any comparisons measured in the same way?	☑	☐	☐	☐
8. Were outcomes measured in a reliable way?	☑	☐	☐	☐
9. Was appropriate statistical analysis used?	☑	☐	☐	☐

Overall appraisal: Include ☑ Exclude ☐ Seek further info ☐

Comments (Including reason for exclusion)

____

____

____

© JBI, 2020. All rights reserved. JBI grants use of these tools for research purposes only. All other enquiries should be sent to jbisynthesis@adelaide.edu.au.

JBI CRITICAL APPRAISAL CHECKLIST FOR QUASI-EXPERIMENTAL STUDIES

Reviewer____________________ Date 11.11.2020

Author Carney et al Year 2016 Record Number_________

	Yes	No	Unclear	Not applicable
1. Is it clear in the study what is the 'cause' and what is the 'effect' (i.e. there is no confusion about which variable comes first)?	☑	☐	☐	☐
2. Were the participants included in any comparisons similar?	☑	☐	☐	☐
3. Were the participants included in any comparisons receiving similar treatment/care, other than the exposure or intervention of interest?	☐	☐	☑	☐
4. Was there a control group?	☐	☑	☐	☐
5. Were there multiple measurements of the outcome both pre and post the intervention/exposure?	☐	☑	☐	☐
6. Was follow up complete and if not, were differences between groups in terms of their follow up adequately described and analyzed?	☐	☐	☐	☑
7. Were the outcomes of participants included in any comparisons measured in the same way?	☑	☐	☐	☐
8. Were outcomes measured in a reliable way?	☑	☐	☐	☐
9. Was appropriate statistical analysis used?	☐	☐	☑	☐

Overall appraisal: Include ☑ Exclude ☐ Seek further info ☐

Comments (Including reason for exclusion)

© JBI, 2020. All rights reserved. JBI grants use of these tools for research purposes only. All other enquiries should be sent to jbisynthesis@adelaide.edu.au.

JBI CRITICAL APPRAISAL CHECKLIST FOR QUASI-EXPERIMENTAL STUDIES

Reviewer_______________________________________ Date 11.11.2020

Author Denissen et al Year 2010 Record Number_________

	Yes	No	Unclear	Not applicable
1. Is it clear in the study what is the 'cause' and what is the 'effect' (i.e. there is no confusion about which variable comes first)?	☑	☐	☐	☐
2. Were the participants included in any comparisons similar?	☑	☐	☐	☐
3. Were the participants included in any comparisons receiving similar treatment/care, other than the exposure or intervention of interest?	☐	☑	☐	☐
4. Was there a control group?	☐	☑	☐	☐
5. Were there multiple measurements of the outcome both pre and post the intervention/exposure?	☐	☑	☐	☐
6. Was follow up complete and if not, were differences between groups in terms of their follow up adequately described and analyzed?	☐	☐	☑	☐
7. Were the outcomes of participants included in any comparisons measured in the same way?	☑	☐	☐	☐
8. Were outcomes measured in a reliable way?	☑	☐	☐	☐
9. Was appropriate statistical analysis used?	☑	☐	☐	☐

Overall appraisal: Include ☑ Exclude ☐ Seek further info ☐

Comments (Including reason for exclusion)

____

____

____

© JBI, 2020. All rights reserved. JBI grants use of these tools for research purposes only. All other enquiries should be sent to jbisynthesis@adelaide.edu.au.

JBI CRITICAL APPRAISAL CHECKLIST FOR ANALYTICAL CROSS SECTIONAL STUDIES

Reviewer________________________ Date 12.11.2020

Author Murro et al Year 2019 Record Number_________

	Yes	No	Unclear	Not applicable
1. Were the criteria for inclusion in the sample clearly defined?	☑	☐	☐	☐
2. Were the study subjects and the setting described in detail?	☐	☐	☑	☐
3. Was the exposure measured in a valid and reliable way?	☑	☐	☐	☐
4. Were objective, standard criteria used for measurement of the condition?	☑	☐	☐	☐
5. Were confounding factors identified?	☐	☐	☑	☐
6. Were strategies to deal with confounding factors stated?	☐	☐	☑	☐
7. Were the outcomes measured in a valid and reliable way?	☐	☑	☐	☐
8. Was appropriate statistical analysis used?	☑	☐	☐	☐

Overall appraisal: Include ☑ Exclude ☐ Seek further info ☐

Comments (Including reason for exclusion)

____

____

____

© JBI, 2020. All rights reserved. JBI grants use of these tools for research purposes only. All other enquiries should be sent to jbisynthesis@adelaide.edu.au.

JBI CRITICAL APPRAISAL CHECKLIST FOR CASE CONTROL STUDIES

Reviewer____________________ Date 15.11.2020

Author Gurrea et al. Year 2016 Record Number_________

	Yes	No	Unclear	Not applicable
1. Were the groups comparable other than the presence of disease in cases or the absence of disease in controls?	☐	☐	☐	☑
2. Were cases and controls matched appropriately?	☑	☐	☐	☐
3. Were the same criteria used for identification of cases and controls?	☐	☐	☑	☐
4. Was exposure measured in a standard, valid and reliable way?	☑	☐	☐	☐
5. Was exposure measured in the same way for cases and controls?	☑	☐	☐	☐
6. Were confounding factors identified?	☑	☐	☐	☐
7. Were strategies to deal with confounding factors stated?	☑	☐	☐	☐
8. Were outcomes assessed in a standard, valid and reliable way for cases and controls?	☑	☐	☐	☐
9. Was the exposure period of interest long enough to be meaningful?	☐	☐	☐	☑
10. Was appropriate statistical analysis used?	☑	☐	☐	☐

Overall appraisal: Include ☑ Exclude ☐ Seek further info ☐

Comments (Including reason for exclusion)

© JBI, 2020. All rights reserved. JBI grants use of these tools for research purposes only. All other enquiries should be sent to jbisynthesis@adelaide.edu.au.

JBI CRITICAL APPRAISAL CHECKLIST FOR QUASI-EXPERIMENTAL STUDIES

Reviewer______________________ Date 15.11.2020

Author Hung He et al. Year 2019 Record Number_________

	Yes	No	Unclear	Not applicable
1. Is it clear in the study what is the 'cause' and what is the 'effect' (i.e. there is no confusion about which variable comes first)?	☑	☐	☐	☐
2. Were the participants included in any comparisons similar?	☑	☐	☐	☐
3. Were the participants included in any comparisons receiving similar treatment/care, other than the exposure or intervention of interest?	☑	☐	☐	☐
4. Was there a control group?	☐	☑	☐	☐
5. Were there multiple measurements of the outcome both pre and post the intervention/exposure?	☑	☐	☐	☐
6. Was follow up complete and if not, were differences between groups in terms of their follow up adequately described and analyzed?	☐	☐	☐	☑
7. Were the outcomes of participants included in any comparisons measured in the same way?	☑	☐	☐	☐
8. Were outcomes measured in a reliable way?	☑	☐	☐	☐
9. Was appropriate statistical analysis used?	☑	☐	☐	☐

Overall appraisal: Include ☑ Exclude ☐ Seek further info ☐

Comments (Including reason for exclusion)

____

____

____

© JBI, 2020. All rights reserved. JBI grants use of these tools for research purposes only. All other enquiries should be sent to jbisynthesis@adelaide.edu.au.

JBI CRITICAL APPRAISAL CHECKLIST FOR QUASI-EXPERIMENTAL STUDIES

Reviewer_______________________ Date 15.11.2020

Author Hein et al. Year 2016 Record Number_________

	Yes	No	Unclear	Not applicable
1. Is it clear in the study what is the 'cause' and what is the 'effect' (i.e. there is no confusion about which variable comes first)?	☐	☐	☑	☐
2. Were the participants included in any comparisons similar?	☑	☐	☐	☐
3. Were the participants included in any comparisons receiving similar treatment/care, other than the exposure or intervention of interest?	☑	☐	☐	☐
4. Was there a control group?	☐	☑	☐	☐
5. Were there multiple measurements of the outcome both pre and post the intervention/exposure?	☐	☑	☐	☐
6. Was follow up complete and if not, were differences between groups in terms of their follow up adequately described and analyzed?	☐	☐	☐	☑
7. Were the outcomes of participants included in any comparisons measured in the same way?	☑	☐	☐	☐
8. Were outcomes measured in a reliable way?	☑	☐	☐	☐
9. Was appropriate statistical analysis used?	☑	☐	☐	☐

Overall appraisal: Include ☑ Exclude ☐ Seek further info ☐

Comments (Including reason for exclusion)

___

___

___

© JBI, 2020. All rights reserved. JBI grants use of these tools for research purposes only. All other enquiries should be sent to jbisynthesis@adelaide.edu.au.

JBI CRITICAL APPRAISAL CHECKLIST FOR ANALYTICAL CROSS SECTIONAL STUDIES

Reviewer_______________________________________ Date 16.11.2020

Author Labban et al. Year 2017 Record Number_________

	Yes	No	Unclear	Not applicable
1. Were the criteria for inclusion in the sample clearly defined?	☑	☐	☐	☐
2. Were the study subjects and the setting described in detail?	☑	☐	☐	☐
3. Was the exposure measured in a valid and reliable way?	☑	☐	☐	☐
4. Were objective, standard criteria used for measurement of the condition?	☑	☐	☐	☐
5. Were confounding factors identified?	☑	☐	☐	☐
6. Were strategies to deal with confounding factors stated?	☑	☐	☐	☐
7. Were the outcomes measured in a valid and reliable way?	☑	☐	☐	☐
8. Was appropriate statistical analysis used?	☑	☐	☐	☐

Overall appraisal: Include ☑ Exclude ☐ Seek further info ☐

Comments (Including reason for exclusion)

____

____

____

© JBI, 2020. All rights reserved. JBI grants use of these tools for research purposes only. All other enquiries should be sent to jbisynthesis@adelaide.edu.au.

JBI CRITICAL APPRAISAL CHECKLIST FOR ANALYTICAL CROSS SECTIONAL STUDIES

Reviewer________________________ Date 16.11.2020

Author Lam et al Year 2018 Record Number________

	Yes	No	Unclear	Not applicable
1. Were the criteria for inclusion in the sample clearly defined?	☑	☐	☐	☐
2. Were the study subjects and the setting described in detail?	☑	☐	☐	☐
3. Was the exposure measured in a valid and reliable way?	☑	☐	☐	☐
4. Were objective, standard criteria used for measurement of the condition?	☑	☐	☐	☐
5. Were confounding factors identified?	☐	☐	☑	☐
6. Were strategies to deal with confounding factors stated?	☐	☐	☑	☐
7. Were the outcomes measured in a valid and reliable way?	☐	☐	☑	☐
8. Was appropriate statistical analysis used?	☐	☐	☐	☑

Overall appraisal: Include ☑ Exclude ☐ Seek further info ☐

Comments (Including reason for exclusion)

© JBI, 2020. All rights reserved. JBI grants use of these tools for research purposes only. All other enquiries should be sent to jbisynthesis@adelaide.edu.au.

Article title: Assessing Tooth Color Differences in Digital Facial Portraits

JBI CRITICAL APPRAISAL CHECKLIST FOR ANALYTICAL CROSS SECTIONAL STUDIES

Reviewer______________________ Date 16.11.20

Author Lindsey et al. Year 2010 Record Number__________

	Yes	No	Unclear	Not applicable
1. Were the criteria for inclusion in the sample clearly defined?	☑	☐	☐	☐
2. Were the study subjects and the setting described in detail?	☐	☐	☑	☐
3. Was the exposure measured in a valid and reliable way?	☑	☐	☐	☐
4. Were objective, standard criteria used for measurement of the condition?	☑	☐	☐	☐
5. Were confounding factors identified?	☐	☐	☑	☐
6. Were strategies to deal with confounding factors stated?	☐	☐	☑	☐
7. Were the outcomes measured in a valid and reliable way?	☑	☐	☐	☐
8. Was appropriate statistical analysis used?	☑	☐	☐	☐

Overall appraisal: Include ☑ Exclude ☐ Seek further info ☐

Comments (Including reason for exclusion)

© JBI, 2020. All rights reserved. JBI grants use of these tools for research purposes only. All other enquiries should be sent to jbisynthesis@adelaide.edu.au.

JBI CRITICAL APPRAISAL CHECKLIST FOR QUASI-EXPERIMENTAL STUDIES

Reviewer ______________________ Date 17.11.2020

Author Luo et al. Year 2017 Record Number ________

	Yes	No	Unclear	Not applicable
1. Is it clear in the study what is the 'cause' and what is the 'effect' (i.e. there is no confusion about which variable comes first)?	☑	☐	☐	☐
2. Were the participants included in any comparisons similar?	☐	☑	☐	☐
3. Were the participants included in any comparisons receiving similar treatment/care, other than the exposure or intervention of interest?	☐	☑	☐	☐
4. Was there a control group?	☐	☑	☐	☐
5. Were there multiple measurements of the outcome both pre and post the intervention/exposure?	☑	☐	☐	☐
6. Was follow up complete and if not, were differences between groups in terms of their follow up adequately described and analyzed?	☐	☐	☐	☑
7. Were the outcomes of participants included in any comparisons measured in the same way?	☑	☐	☐	☐
8. Were outcomes measured in a reliable way?	☑	☐	☐	☐
9. Was appropriate statistical analysis used?	☑	☐	☐	☐

Overall appraisal: Include ☑ Exclude ☐ Seek further info ☐

Comments (Including reason for exclusion)

____

____

____

© JBI, 2020. All rights reserved. JBI grants use of these tools for research purposes only. All other enquiries should be sent to jbisynthesis@adelaide.edu.au.

JBI CRITICAL APPRAISAL CHECKLIST FOR ANALYTICAL CROSS SECTIONAL STUDIES

Reviewer____________________ Date 17.11.2020

Author Maddula et al. Year 2018 Record Number_________

	Yes	No	Unclear	Not applicable
1. Were the criteria for inclusion in the sample clearly defined?	☐	☐	☑	☐
2. Were the study subjects and the setting described in detail?	☐	☑	☐	☐
3. Was the exposure measured in a valid and reliable way?	☑	☐	☐	☐
4. Were objective, standard criteria used for measurement of the condition?	☐	☐	☑	☐
5. Were confounding factors identified?	☐	☑	☐	☐
6. Were strategies to deal with confounding factors stated?	☐	☑	☐	☐
7. Were the outcomes measured in a valid and reliable way?	☑	☐	☐	☐
8. Was appropriate statistical analysis used?	☐	☐	☑	☐

Overall appraisal: Include ☐ Exclude ☐ Seek further info ☐

Comments (Including reason for exclusion)

© JBI, 2020. All rights reserved. JBI grants use of these tools for research purposes only. All other enquiries should be sent to jbisynthesis@adelaide.edu.au.

JBI CRITICAL APPRAISAL CHECKLIST FOR ANALYTICAL CROSS SECTIONAL STUDIES

Reviewer_______________________ Date 17.11.2020

Author Mahn et al. Year 2020 Record Number_________

	Yes	No	Unclear	Not applicable
1. Were the criteria for inclusion in the sample clearly defined?	☒	☐	☐	☐
2. Were the study subjects and the setting described in detail?	☒	☐	☐	☐
3. Was the exposure measured in a valid and reliable way?	☒	☐	☐	☐
4. Were objective, standard criteria used for measurement of the condition?	☒	☐	☐	☐
5. Were confounding factors identified?	☐	☐	☒	☐
6. Were strategies to deal with confounding factors stated?	☐	☐	☒	☐
7. Were the outcomes measured in a valid and reliable way?	☒	☐	☐	☐
8. Was appropriate statistical analysis used?	☒	☐	☐	☐

Overall appraisal: Include ☒ Exclude ☐ Seek further info ☐

Comments (Including reason for exclusion)

____

____

____

© JBI, 2020. All rights reserved. JBI grants use of these tools for research purposes only. All other enquiries should be sent to jbisynthesis@adelaide.edu.au.

JBI CRITICAL APPRAISAL CHECKLIST FOR ANALYTICAL CROSS SECTIONAL STUDIES

Reviewer______________________ Date 19.11.2020

Author Montero et al. Year 2014 Record Number__________

	Yes	No	Unclear	Not applicable
1. Were the criteria for inclusion in the sample clearly defined?	☑	☐	☐	☐
2. Were the study subjects and the setting described in detail?	☐	☐	☑	☐
3. Was the exposure measured in a valid and reliable way?	☐	☐	☑	☐
4. Were objective, standard criteria used for measurement of the condition?	☑	☐	☐	☐
5. Were confounding factors identified?	☐	☐	☑	☐
6. Were strategies to deal with confounding factors stated?	☐	☐	☑	☐
7. Were the outcomes measured in a valid and reliable way?	☐	☑	☐	☐
8. Was appropriate statistical analysis used?	☑	☐	☐	☐

Overall appraisal: Include ☑ Exclude ☐ Seek further info ☐

Comments (Including reason for exclusion)

____

____

____

© JBI, 2020. All rights reserved. JBI grants use of these tools for research purposes only. All other enquiries should be sent to jbisynthesis@adelaide.edu.au.

JBI CRITICAL APPRAISAL CHECKLIST FOR QUASI-EXPERIMENTAL STUDIES

Reviewer_______________________ Date 21.11.2020

Author Smith et al Year 2008 Record Number_________

	Yes	No	Unclear	Not applicable
1. Is it clear in the study what is the 'cause' and what is the 'effect' (i.e. there is no confusion about which variable comes first)?	☑	☐	☐	☐
2. Were the participants included in any comparisons similar?	☑	☐	☐	☐
3. Were the participants included in any comparisons receiving similar treatment/care, other than the exposure or intervention of interest?	☐	☐	☑	☐
4. Was there a control group?	☐	☑	☐	☐
5. Were there multiple measurements of the outcome both pre and post the intervention/exposure?	☑	☐	☐	☐
6. Was follow up complete and if not, were differences between groups in terms of their follow up adequately described and analyzed?	☐	☐	☐	☑
7. Were the outcomes of participants included in any comparisons measured in the same way?	☑	☐	☐	☐
8. Were outcomes measured in a reliable way?	☑	☐	☐	☐
9. Was appropriate statistical analysis used?	☑	☐	☐	☐

Overall appraisal: Include ☑ Exclude ☐ Seek further info ☐

Comments (Including reason for exclusion)

____

____

____

© JBI, 2020. All rights reserved. JBI grants use of these tools for research purposes only. All other enquiries should be sent to jbisynthesis@adelaide.edu.au.

JBI CRITICAL APPRAISAL CHECKLIST FOR QUASI-EXPERIMENTAL STUDIES

Reviewer______________________ Date 22.11.2020

Author Tam et al Year 2016 Record Number_________

	Yes	No	Unclear	Not applicable
1. Is it clear in the study what is the 'cause' and what is the 'effect' (i.e. there is no confusion about which variable comes first)?	☑	☐	☐	☐
2. Were the participants included in any comparisons similar?	☑	☐	☐	☐
3. Were the participants included in any comparisons receiving similar treatment/care, other than the exposure or intervention of interest?	☐	☐	☐	☑
4. Was there a control group?	☐	☑	☐	☐
5. Were there multiple measurements of the outcome both pre and post the intervention/exposure?	☐	☑	☐	☐
6. Was follow up complete and if not, were differences between groups in terms of their follow up adequately described and analyzed?	☐	☐	☐	☑
7. Were the outcomes of participants included in any comparisons measured in the same way?	☑	☐	☐	☐
8. Were outcomes measured in a reliable way?	☑	☐	☐	☐
9. Was appropriate statistical analysis used?	☑	☐	☐	☐

Overall appraisal: Include ☑ Exclude ☐ Seek further info ☐

Comments (Including reason for exclusion)

____

____

____

© JBI, 2020. All rights reserved. JBI grants use of these tools for research purposes only. All other enquiries should be sent to jbisynthesis@adelaide.edu.au.

JBI CRITICAL APPRAISAL CHECKLIST FOR QUASI-EXPERIMENTAL STUDIES

Reviewer_______________________________________ Date 23.11.2020

Author Tung et al. Year 2010 Record Number_________

	Yes	No	Unclear	Not applicable
1. Is it clear in the study what is the 'cause' and what is the 'effect' (i.e. there is no confusion about which variable comes first)?	☑	☐	☐	☐
2. Were the participants included in any comparisons similar?	☑	☐	☐	☐
3. Were the participants included in any comparisons receiving similar treatment/care, other than the exposure or intervention of interest?	☑	☐	☐	☐
4. Was there a control group?	☐	☐	☑	☐
5. Were there multiple measurements of the outcome both pre and post the intervention/exposure?	☐	☐	☐	☑
6. Was follow up complete and if not, were differences between groups in terms of their follow up adequately described and analyzed?	☐	☐	☐	☑
7. Were the outcomes of participants included in any comparisons measured in the same way?	☑	☐	☐	☐
8. Were outcomes measured in a reliable way?	☑	☐	☐	☐
9. Was appropriate statistical analysis used?	☑	☐	☐	☐

Overall appraisal: Include ☐ Exclude ☐ Seek further info ☐

Comments (Including reason for exclusion)

____

____

____

© JBI, 2020. All rights reserved. JBI grants use of these tools for research purposes only. All other enquiries should be sent to jbisynthesis@adelaide.edu.au.

JBI CRITICAL APPRAISAL CHECKLIST FOR QUASI-EXPERIMENTAL STUDIES

Reviewer________________ Date 24.11.2020

Author Liu et al Year 2019 Record Number_________

	Yes	No	Unclear	Not applicable
1. Is it clear in the study what is the 'cause' and what is the 'effect' (i.e. there is no confusion about which variable comes first)?	☑	☐	☐	☐
2. Were the participants included in any comparisons similar?	☑	☐	☐	☐
3. Were the participants included in any comparisons receiving similar treatment/care, other than the exposure or intervention of interest?	☑	☐	☐	☐
4. Was there a control group?	☐	☐	☑	☐
5. Were there multiple measurements of the outcome both pre and post the intervention/exposure?	☐	☐	☐	☑
6. Was follow up complete and if not, were differences between groups in terms of their follow up adequately described and analyzed?	☐	☐	☐	☑
7. Were the outcomes of participants included in any comparisons measured in the same way?	☑	☐	☐	☐
8. Were outcomes measured in a reliable way?	☑	☐	☐	☐
9. Was appropriate statistical analysis used?	☑	☐	☐	☐

Overall appraisal: Include ☑ Exclude ☐ Seek further info ☐

Comments (Including reason for exclusion)

____

____

____

© JBI, 2020. All rights reserved. JBI grants use of these tools for research purposes only. All other enquiries should be sent to jbisynthesis@adelaide.edu.au.

Article title: Comparative Analysis of Color Matching System for Teeth Recognition Using Color Moment

JBI CRITICAL APPRAISAL CHECKLIST FOR QUASI-EXPERIMENTAL STUDIES

Reviewer_______________________ Date 24.11.20

Author Justiawan et al. Year 2019 Record Number_________

	Yes	No	Unclear	Not applicable
1. Is it clear in the study what is the 'cause' and what is the 'effect' (i.e. there is no confusion about which variable comes first)?	☑	☐	☐	☐
2. Were the participants included in any comparisons similar?	☑	☐	☐	☐
3. Were the participants included in any comparisons receiving similar treatment/care, other than the exposure or intervention of interest?	☐	☐	☑	☐
4. Was there a control group?	☐	☑	☐	☐
5. Were there multiple measurements of the outcome both pre and post the intervention/exposure?	☐	☑	☐	☐
6. Was follow up complete and if not, were differences between groups in terms of their follow up adequately described and analyzed?	☐	☐	☐	☑
7. Were the outcomes of participants included in any comparisons measured in the same way?	☑	☐	☐	☐
8. Were outcomes measured in a reliable way?	☑	☐	☐	☐
9. Was appropriate statistical analysis used?	☑	☐	☐	☐

Overall appraisal: Include ☑ Exclude ☐ Seek further info ☐

Comments (Including reason for exclusion)

___

___

___

© JBI, 2020. All rights reserved. JBI grants use of these tools for research purposes only. All other enquiries should be sent to jbisynthesis@adelaide.edu.au.

JBI CRITICAL APPRAISAL CHECKLIST FOR QUASI-EXPERIMENTAL STUDIES

Reviewer______________________ Date 26.11.2020

Author Lin et al. Year 2019 Record Number_________

	Yes	No	Unclear	Not applicable
1. Is it clear in the study what is the 'cause' and what is the 'effect' (i.e. there is no confusion about which variable comes first)?	☒	☐	☐	☐
2. Were the participants included in any comparisons similar?	☒	☐	☐	☐
3. Were the participants included in any comparisons receiving similar treatment/care, other than the exposure or intervention of interest?	☐	☐	☐	☒
4. Was there a control group?	☐	☒	☐	☐
5. Were there multiple measurements of the outcome both pre and post the intervention/exposure?	☒	☐	☐	☐
6. Was follow up complete and if not, were differences between groups in terms of their follow up adequately described and analyzed?	☐	☐	☐	☒
7. Were the outcomes of participants included in any comparisons measured in the same way?	☒	☐	☐	☐
8. Were outcomes measured in a reliable way?	☒	☐	☐	☐
9. Was appropriate statistical analysis used?	☐	☐	☒	☐

Overall appraisal: Include ☒ Exclude ☐ Seek further info ☐

Comments (Including reason for exclusion)

____

____

____

© JBI, 2020. All rights reserved. JBI grants use of these tools for research purposes only. All other enquiries should be sent to jbisynthesis@adelaide.edu.au.

JBI CRITICAL APPRAISAL CHECKLIST FOR QUASI-EXPERIMENTAL STUDIES

Reviewer______________________________________ Date 25.11.2020

Author Kim et al. Year 2018 Record Number_________

	Yes	No	Unclear	Not applicable
1. Is it clear in the study what is the 'cause' and what is the 'effect' (i.e. there is no confusion about which variable comes first)?	☐	☐	☒	☐
2. Were the participants included in any comparisons similar?	☒	☐	☐	☐
3. Were the participants included in any comparisons receiving similar treatment/care, other than the exposure or intervention of interest?	☐	☐	☒	☐
4. Was there a control group?	☐	☒	☐	☐
5. Were there multiple measurements of the outcome both pre and post the intervention/exposure?	☐	☒	☐	☐
6. Was follow up complete and if not, were differences between groups in terms of their follow up adequately described and analyzed?	☐	☐	☐	☒
7. Were the outcomes of participants included in any comparisons measured in the same way?	☒	☐	☐	☐
8. Were outcomes measured in a reliable way?	☒	☐	☐	☐
9. Was appropriate statistical analysis used?	☒	☐	☐	☐

Overall appraisal: Include ☒ Exclude ☐ Seek further info ☐

Comments (Including reason for exclusion)

____

____

____

© JBI, 2020. All rights reserved. JBI grants use of these tools for research purposes only. All other enquiries should be sent to jbisynthesis@adelaide.edu.au.

Endodontics and Dental esthetics

JBI CRITICAL APPRAISAL CHECKLIST FOR ANALYTICAL CROSS SECTIONAL STUDIES

Reviewer________________________ Date 2.12.20

Author Bentley et al. Year 1999 Record Number__________

	Yes	No	Unclear	Not applicable
1. Were the criteria for inclusion in the sample clearly defined?	☑	☐	☐	☐
2. Were the study subjects and the setting described in detail?	☑	☐	☐	☐
3. Was the exposure measured in a valid and reliable way?	☑	☐	☐	☐
4. Were objective, standard criteria used for measurement of the condition?	☑	☐	☐	☐
5. Were confounding factors identified?	☐	☐	☑	☐
6. Were strategies to deal with confounding factors stated?	☐	☐	☑	☐
7. Were the outcomes measured in a valid and reliable way?	☑	☐	☐	☐
8. Was appropriate statistical analysis used?	☑	☐	☐	☐

Overall appraisal: Include ☑ Exclude ☐ Seek further info ☐

Comments (Including reason for exclusion)

© JBI, 2020. All rights reserved. JBI grants use of these tools for research purposes only. All other enquiries should be sent to jbisynthesis@adelaide.edu.au.

JBI CRITICAL APPRAISAL CHECKLIST FOR QUASI-EXPERIMENTAL STUDIES

Reviewer__________ Date 2.12.2020

Author Gadhia et al. Year 2006 Record Number__________

	Yes	No	Unclear	Not applicable
1. Is it clear in the study what is the 'cause' and what is the 'effect' (i.e. there is no confusion about which variable comes first)?	☑	☐	☐	☐
2. Were the participants included in any comparisons similar?	☑	☐	☐	☐
3. Were the participants included in any comparisons receiving similar treatment/care, other than the exposure or intervention of interest?	☑	☐	☐	☐
4. Was there a control group?	☐	☐	☑	☐
5. Were there multiple measurements of the outcome both pre and post the intervention/exposure?	☑	☐	☐	☐
6. Was follow up complete and if not, were differences between groups in terms of their follow up adequately described and analyzed?	☐	☐	☑	☐
7. Were the outcomes of participants included in any comparisons measured in the same way?	☐	☑	☐	☐
8. Were outcomes measured in a reliable way?	☑	☐	☐	☐
9. Was appropriate statistical analysis used?	☑	☐	☐	☐

Overall appraisal: Include ☑ Exclude ☐ Seek further info ☐

Comments (Including reason for exclusion)

© JBI, 2020. All rights reserved. JBI grants use of these tools for research purposes only. All other enquiries should be sent to jbisynthesis@adelaide.edu.au.

JBI CRITICAL APPRAISAL CHECKLIST FOR QUASI-EXPERIMENTAL STUDIES

Reviewer____________________ Date 3.12.2020

Author Garg et al. Year 2020 Record Number_________

	Yes	No	Unclear	Not applicable
1. Is it clear in the study what is the 'cause' and what is the 'effect' (i.e. there is no confusion about which variable comes first)?	☑	☐	☐	☐
2. Were the participants included in any comparisons similar?	☑	☐	☐	☐
3. Were the participants included in any comparisons receiving similar treatment/care, other than the exposure or intervention of interest?	☑	☐	☐	☐
4. Was there a control group?	☐	☑	☐	☐
5. Were there multiple measurements of the outcome both pre and post the intervention/exposure?	☑	☐	☐	☐
6. Was follow up complete and if not, were differences between groups in terms of their follow up adequately described and analyzed?	☑	☐	☐	☐
7. Were the outcomes of participants included in any comparisons measured in the same way?	☑	☐	☐	☐
8. Were outcomes measured in a reliable way?	☑	☐	☐	☐
9. Was appropriate statistical analysis used?	☑	☐	☐	☐

Overall appraisal: Include ☑ Exclude ☐ Seek further info ☐

Comments (Including reason for exclusion)

____

____

____

© JBI, 2020. All rights reserved. JBI grants use of these tools for research purposes only. All other enquiries should be sent to jbisynthesis@adelaide.edu.au.

Article title: The measurement of tooth whiteness by image analysis and spectrophotometry: a comparison

JBI CRITICAL APPRAISAL CHECKLIST FOR QUASI-EXPERIMENTAL STUDIES

Reviewer_______________________ Date 10.12.2020

Author Guan et al. Year 2003 Record Number_________

	Yes	No	Unclear	Not applicable
1. Is it clear in the study what is the 'cause' and what is the 'effect' (i.e. there is no confusion about which variable comes first)?	☐	☐	☑	☐
2. Were the participants included in any comparisons similar?	☑	☐	☐	☐
3. Were the participants included in any comparisons receiving similar treatment/care, other than the exposure or intervention of interest?	☑	☐	☐	☐
4. Was there a control group?	☐	☐	☑	☐
5. Were there multiple measurements of the outcome both pre and post the intervention/exposure?	☑	☐	☐	☐
6. Was follow up complete and if not, were differences between groups in terms of their follow up adequately described and analyzed?	☐	☐	☐	☑
7. Were the outcomes of participants included in any comparisons measured in the same way?	☑	☐	☐	☐
8. Were outcomes measured in a reliable way?	☑	☐	☐	☐
9. Was appropriate statistical analysis used?	☑	☐	☐	☐

Overall appraisal: Include ☑ Exclude ☐ Seek further info ☐

Comments (Including reason for exclusion)

____

____

____

© JBI, 2020. All rights reserved. JBI grants use of these tools for research purposes only. All other enquiries should be sent to jbisynthesis@adelaide.edu.au.

JBI CRITICAL APPRAISAL CHECKLIST FOR QUASI-EXPERIMENTAL STUDIES

Reviewer______________________________ Date 4.5.20______________________

Author Jarad et al.______________________ Year 2008______ Record Number_________

	Yes	No	Unclear	Not applicable
1. Is it clear in the study what is the 'cause' and what is the 'effect' (i.e. there is no confusion about which variable comes first)?	☐	☐	☑	☐
2. Were the participants included in any comparisons similar?	☑	☐	☐	☐
3. Were the participants included in any comparisons receiving similar treatment/care, other than the exposure or intervention of interest?	☐	☐	☑	☐
4. Was there a control group?	☐	☑	☐	☐
5. Were there multiple measurements of the outcome both pre and post the intervention/exposure?	☐	☐	☑	☐
6. Was follow up complete and if not, were differences between groups in terms of their follow up adequately described and analyzed?	☐	☐	☐	☑
7. Were the outcomes of participants included in any comparisons measured in the same way?	☑	☐	☐	☐
8. Were outcomes measured in a reliable way?	☑	☐	☐	☐
9. Was appropriate statistical analysis used?	☑	☐	☐	☐

Overall appraisal: Include ☑ Exclude ☐ Seek further info ☐

Comments (Including reason for exclusion)

____

____

____

© JBI, 2020. All rights reserved. JBI grants use of these tools for research purposes only. All other enquiries should be sent to jbisynthesis@adelaide.edu.au.

JBI CRITICAL APPRAISAL CHECKLIST FOR ANALYTICAL CROSS SECTIONAL STUDIES

Reviewer______________________________________ Date 5.12.2020______________________________

Author Kim et al.______________________________________ Year 2017______ Record Number__________

	Yes	No	Unclear	Not applicable
1. Were the criteria for inclusion in the sample clearly defined?	☑	☐	☐	☐
2. Were the study subjects and the setting described in detail?	☑	☐	☐	☐
3. Was the exposure measured in a valid and reliable way?	☑	☐	☐	☐
4. Were objective, standard criteria used for measurement of the condition?	☑	☐	☐	☐
5. Were confounding factors identified?	☐	☐	☑	☐
6. Were strategies to deal with confounding factors stated?	☐	☐	☑	☐
7. Were the outcomes measured in a valid and reliable way?	☑	☐	☐	☐
8. Was appropriate statistical analysis used?	☑	☐	☐	☐

Overall appraisal: Include ☑ Exclude ☐ Seek further info ☐

Comments (Including reason for exclusion)

© JBI, 2020. All rights reserved. JBI grants use of these tools for research purposes only. All other enquiries should be sent to jbisynthesis@adelaide.edu.au.

JBI CRITICAL APPRAISAL CHECKLIST FOR QUASI-EXPERIMENTAL STUDIES

Reviewer_______________________ Date 05.12.2020

Author Kuzekanani et al. Year 2009 Record Number_________

	Yes	No	Unclear	Not applicable
1. Is it clear in the study what is the 'cause' and what is the 'effect' (i.e. there is no confusion about which variable comes first)?	☑	☐	☐	☐
2. Were the participants included in any comparisons similar?	☑	☐	☐	☐
3. Were the participants included in any comparisons receiving similar treatment/care, other than the exposure or intervention of interest?	☑	☐	☐	☐
4. Was there a control group?	☐	☑	☐	☐
5. Were there multiple measurements of the outcome both pre and post the intervention/exposure?	☑	☐	☐	☐
6. Was follow up complete and if not, were differences between groups in terms of their follow up adequately described and analyzed?	☐	☐	☑	☐
7. Were the outcomes of participants included in any comparisons measured in the same way?	☑	☐	☐	☐
8. Were outcomes measured in a reliable way?	☑	☐	☐	☐
9. Was appropriate statistical analysis used?	☐	☐	☐	☐

Overall appraisal: Include ☑ Exclude ☐ Seek further info ☐

Comments (Including reason for exclusion)

____

____

____

© JBI, 2020. All rights reserved. JBI grants use of these tools for research purposes only. All other enquiries should be sent to jbisynthesis@adelaide.edu.au.

JBI CRITICAL APPRAISAL CHECKLIST FOR QUASI-EXPERIMENTAL STUDIES

Reviewer______________________ Date 06.12.2020______________________

Author Lee et al.______________________ Year 2007______ Record Number__________

	Yes	No	Unclear	Not applicable
1. Is it clear in the study what is the 'cause' and what is the 'effect' (i.e. there is no confusion about which variable comes first)?	☒	☐	☐	☐
2. Were the participants included in any comparisons similar?	☒	☐	☐	☐
3. Were the participants included in any comparisons receiving similar treatment/care, other than the exposure or intervention of interest?	☒	☐	☐	☐
4. Was there a control group?	☐	☒	☐	☐
5. Were there multiple measurements of the outcome both pre and post the intervention/exposure?	☒	☐	☐	☐
6. Was follow up complete and if not, were differences between groups in terms of their follow up adequately described and analyzed?	☐	☐	☐	☒
7. Were the outcomes of participants included in any comparisons measured in the same way?	☒	☐	☐	☐
8. Were outcomes measured in a reliable way?	☒	☐	☐	☐
9. Was appropriate statistical analysis used?	☒	☐	☐	☐

Overall appraisal: Include ☒ Exclude ☐ Seek further info ☐

Comments (Including reason for exclusion)

____

____

____

© JBI, 2020. All rights reserved. JBI grants use of these tools for research purposes only. All other enquiries should be sent to jbisynthesis@adelaide.edu.au.

Prosthodontics

JBI CRITICAL APPRAISAL CHECKLIST FOR COHORT STUDIES

Reviewer______________________ Date 31.10.2020

Author Yousof et al. Year 2019 Record Number 1

	Yes	No	Unclear	Not applicable
1. Were the two groups similar and recruited from the same population?	☐	☑	☐	☐
2. Were the exposures measured similarly to assign people<br>3. to both exposed and unexposed groups?	☐	☑	☐	☐
4. Was the exposure measured in a valid and reliable way?	☐	☐	☑	☐
5. Were confounding factors identified?	☐	☐	☑	☐
6. Were strategies to deal with confounding factors stated?	☐	☐	☑	☐
7. Were the groups/participants free of the outcome at the start of the study (or at the moment of exposure)?	☐	☐	☐	☑
8. Were the outcomes measured in a valid and reliable way?	☑	☐	☐	☐
9. Was the follow up time reported and sufficient to be long enough for outcomes to occur?	☐	☐	☐	☑
10. Was follow up complete, and if not, were the reasons to loss to follow up described and explored?	☐	☐	☐	☑
11. Were strategies to address incomplete follow up utilized?	☐	☐	☐	☑
12. Was appropriate statistical analysis used?	☑	☐	☐	☐

Overall appraisal: Include ☑ Exclude ☐ Seek further info ☐

Comments (Including reason for exclusion)

© JBI, 2020. All rights reserved. JBI grants use of these tools for research purposes only. All other enquiries should be sent to jbisynthesis@adelaide.edu.au.

JBI CRITICAL APPRAISAL CHECKLIST FOR COHORT STUDIES

Reviewer______________________________________ Date 14.10.2020

Author BILT* et al Year 2012 Record Number 2

	Yes	No	Unclear	Not applicable
1. Were the two groups similar and recruited from the same population?	☑	☐	☐	☐
2. Were the exposures measured similarly to assign people<br>3. to both exposed and unexposed groups?	☑	☐	☐	☐
4. Was the exposure measured in a valid and reliable way?	☑	☐	☐	☐
5. Were confounding factors identified?	☐	☐	☑	☐
6. Were strategies to deal with confounding factors stated?	☐	☐	☑	☐
7. Were the groups/participants free of the outcome at the start of the study (or at the moment of exposure)?	☐	☐	☐	☑
8. Were the outcomes measured in a valid and reliable way?	☑	☐	☐	☐
9. Was the follow up time reported and sufficient to be long enough for outcomes to occur?	☐	☐	☐	☑
10. Was follow up complete, and if not, were the reasons to loss to follow up described and explored?	☐	☐	☐	☑
11. Were strategies to address incomplete follow up utilized?	☐	☐	☐	☑
12. Was appropriate statistical analysis used?	☑	☐	☐	☐

Overall appraisal: Include ☑ Exclude ☐ Seek further info ☐

Comments (Including reason for exclusion)

____

____

© JBI, 2020. All rights reserved. JBI grants use of these tools for research purposes only. All other enquiries should be sent to jbisynthesis@adelaide.edu.au.

Article title: Intraoral infrared color photography of radiotherapy patients

JBI Critical Appraisal Checklist for Case Series

Reviewer_______________________ Date 14.10.2020

Author Maccarty et al. Year 1976 Record Number_________

	Yes	No	Unclear	Not applicable
• Were there clear criteria for inclusion in the case series?	☒	☐	☐	☐
• Was the condition measured in a standard, reliable way for all participants included in the case series?	☐	☐	☒	☐
• Were valid methods used for identification of the condition for all participants included in the case series?	☐	☐	☒	☐
• Did the case series have consecutive inclusion of participants?	☐	☒	☐	☐
• Did the case series have complete inclusion of participants?	☐	☐	☒	☐
• Was there clear reporting of the demographics of the participants in the study?	☒	☐	☐	☐
• Was there clear reporting of clinical information of the participants?	☒	☐	☐	☐
• Were the outcomes or follow up results of cases clearly reported?	☒	☐	☐	☐
• Was there clear reporting of the presenting site(s)/clinic(s) demographic information?	☐	☒	☐	☐
• Was statistical analysis appropriate?	☐	☐	☒	☐

Overall appraisal: Include ☒ Exclude ☐ Seek further info ☐

Comments (Including reason for exclusion)

___

___

© JBI, 2020. All rights reserved. JBI grants use of these tools for research purposes only. All other enquiries should be sent to jbisynthesis@adelaide.edu.au.

Maxillofacial Prostheses

Article title: Perceptibility and acceptability thresholds of simulated facial skin color differences

JBI CRITICAL APPRAISAL CHECKLIST FOR ANALYTICAL CROSS SECTIONAL STUDIES

Reviewer________________________Date 16.09.2020

Author Lagouvardos et al Year 2018 Record Number 1

	Yes	No	Unclear	Not applicable
1. Were the criteria for inclusion in the sample clearly defined?	☑	☐	☐	☐
2. Were the study subjects and the setting described in detail?	☑	☐	☐	☐
3. Was the exposure measured in a valid and reliable way?	☑	☐	☐	☐
4. Were objective, standard criteria used for measurement of the condition?	☑	☐	☐	☐
5. Were confounding factors identified?	☑	☐	☐	☐
6. Were strategies to deal with confounding factors stated?	☐	☐	☑	☐
7. Were the outcomes measured in a valid and reliable way?	☐	☑	☐	☐
8. Was appropriate statistical analysis used?	☑	☐	☐	☐

Overall appraisal: Include ☑ Exclude ☐ Seek further info ☐

Comments (Including reason for exclusion)

© JBI, 2020. All rights reserved. JBI grants use of these tools for research purposes only. All other enquiries should be sent to jbisynthesis@adelaide.edu.au.

Article title: Suitability of a Mobile Phone Colorimeter Application for Use as an Objective Aid when Matching Skin Color during the Fabrication of a Maxillofacial Prosthesis

JBI CRITICAL APPRAISAL CHECKLIST FOR COHORT STUDIES

Reviewer________________________ Date 16.092020

Author Mulcare et al Year 2018 Record Number 2

	Yes	No	Unclear	Not applicable
1. Were the two groups similar and recruited from the same population?	☑	☐	☐	☐
2. Were the exposures measured similarly to assign people<br>3. to both exposed and unexposed groups?	☑	☐	☐	☐
4. Was the exposure measured in a valid and reliable way?	☑	☐	☐	☐
5. Were confounding factors identified?	☑	☐	☐	☐
6. Were strategies to deal with confounding factors stated?	☑	☐	☐	☐
7. Were the groups/participants free of the outcome at the start of the study (or at the moment of exposure)?	☐	☐	☑	☐
8. Were the outcomes measured in a valid and reliable way?	☐	☐	☑	☐
9. Was the follow up time reported and sufficient to be long enough for outcomes to occur?	☐	☐	☐	☑
10. Was follow up complete, and if not, were the reasons to loss to follow up described and explored?	☐	☐	☐	☑
11. Were strategies to address incomplete follow up utilized?	☐	☐	☐	☑
12. Was appropriate statistical analysis used?	☑	☐	☐	☐

Overall appraisal: Include ☑ Exclude ☐ Seek further info ☐

Comments (Including reason for exclusion)

____

____

© JBI, 2020. All rights reserved. JBI grants use of these tools for research purposes only. All other enquiries should be sent to jbisynthesis@adelaide.edu.au.

JBI CRITICAL APPRAISAL CHECKLIST FOR QUASI-EXPERIMENTAL STUDIES

Reviewer_______________________ Date 17.09.2020

Author Sohaib et al Year 2018 Record Number 3

	Yes	No	Unclear	Not applicable
1. Is it clear in the study what is the 'cause' and what is the 'effect' (i.e. there is no confusion about which variable comes first)?	☑	☐	☐	☐
2. Were the participants included in any comparisons similar?	☑	☐	☐	☐
3. Were the participants included in any comparisons receiving similar treatment/care, other than the exposure or intervention of interest?	☑	☐	☐	☐
4. Was there a control group?	☐	☐	☑	☐
5. Were there multiple measurements of the outcome both pre and post the intervention/exposure?	☑	☐	☐	☐
6. Was follow up complete and if not, were differences between groups in terms of their follow up adequately described and analyzed?	☐	☐	☐	☑
7. Were the outcomes of participants included in any comparisons measured in the same way?	☑	☐	☐	☐
8. Were outcomes measured in a reliable way?	☑	☐	☐	☐
9. Was appropriate statistical analysis used?	☐	☐	☑	☐

Overall appraisal: Include ☑ Exclude ☐ Seek further info ☐

Comments (Including reason for exclusion)

____

____

____

© JBI, 2020. All rights reserved. JBI grants use of these tools for research purposes only. All other enquiries should be sent to jbisynthesis@adelaide.edu.au.

JBI CRITICAL APPRAISAL CHECKLIST FOR CASE REPORTS

Reviewer______________________ Date 17.09.2020

Author PRICOP et al Year 2016 Record Number 4

	Yes	No	Unclear	Not applicable
1. Were patient's demographic characteristics clearly described?	☐	☐	☑	☐
2. Was the patient's history clearly described and presented as a timeline?	☐	☑	☐	☐
3. Was the current clinical condition of the patient on presentation clearly described?	☐	☐	☑	☐
4. Were diagnostic tests or assessment methods and the results clearly described?	☐	☐	☑	☐
5. Was the intervention(s) or treatment procedure(s) clearly described?	☑	☐	☐	☐
6. Was the post-intervention clinical condition clearly described?	☐	☐	☑	☐
7. Were adverse events (harms) or unanticipated events identified and described?	☐	☑	☐	☐
8. Does the case report provide takeaway lessons?	☑	☐	☐	☐

Overall appraisal: Include ☑ Exclude ☐ Seek further info ☐

Comments (Including reason for exclusion)

____

____

____

© JBI, 2020. All rights reserved. JBI grants use of these tools for research purposes only. All other enquiries should be sent to jbisynthesis@adelaide.edu.au.

JBI CRITICAL APPRAISAL CHECKLIST FOR CASE REPORTS

Reviewer______________________ Date 21.09.2020

Author Grant et al Year 2015 Record Number 5

	Yes	No	Unclear	Not applicable
1. Were patient's demographic characteristics clearly described?	☑	☐	☐	☐
2. Was the patient's history clearly described and presented as a timeline?	☑	☐	☐	☐
3. Was the current clinical condition of the patient on presentation clearly described?	☑	☐	☐	☐
4. Were diagnostic tests or assessment methods and the results clearly described?	☐	☐	☑	☐
5. Was the intervention(s) or treatment procedure(s) clearly described?	☑	☐	☐	☐
6. Was the post-intervention clinical condition clearly described?	☑	☐	☐	☐
7. Were adverse events (harms) or unanticipated events identified and described?	☐	☐	☑	☐
8. Does the case report provide takeaway lessons?	☑	☐	☐	☐

Overall appraisal: Include ☑ Exclude ☐ Seek further info ☐

Comments (Including reason for exclusion)

____

____

____

© JBI, 2020. All rights reserved. JBI grants use of these tools for research purposes only. All other enquiries should be sent to jbisynthesis@adelaide.edu.au.

JBI CRITICAL APPRAISAL CHECKLIST FOR QUASI-EXPERIMENTAL STUDIES

Reviewer______________________ Date 18.09.2020

Author Xiao et al Year 2014 Record Number 6

	Yes	No	Unclear	Not applicable
1. Is it clear in the study what is the 'cause' and what is the 'effect' (i.e. there is no confusion about which variable comes first)?	☐	☐	☑	☐
2. Were the participants included in any comparisons similar?	☑	☐	☐	☐
3. Were the participants included in any comparisons receiving similar treatment/care, other than the exposure or intervention of interest?	☑	☐	☐	☐
4. Was there a control group?	☐	☐	☑	☐
5. Were there multiple measurements of the outcome both pre and post the intervention/exposure?	☑	☐	☐	☐
6. Was follow up complete and if not, were differences between groups in terms of their follow up adequately described and analyzed?	☐	☐	☐	☑
7. Were the outcomes of participants included in any comparisons measured in the same way?	☑	☐	☐	☐
8. Were outcomes measured in a reliable way?	☑	☐	☐	☐
9. Was appropriate statistical analysis used?	☐	☐	☐	☑

Overall appraisal: Include ☑ Exclude ☐ Seek further info ☐

Comments (Including reason for exclusion)

____

____

____

© JBI, 2020. All rights reserved. JBI grants use of these tools for research purposes only. All other enquiries should be sent to jbisynthesis@adelaide.edu.au.

JBI CRITICAL APPRAISAL CHECKLIST FOR CASE REPORTS

Reviewer________________ Date 21.09.2020

Author Buzayan et al Year 2014 Record Number 7

	Yes	No	Unclear	Not applicable
1. Were patient's demographic characteristics clearly described?	☐	☐	☑	☐
2. Was the patient's history clearly described and presented as a timeline?	☐	☐	☑	☐
3. Was the current clinical condition of the patient on presentation clearly described?	☐	☐	☑	☐
4. Were diagnostic tests or assessment methods and the results clearly described?	☑	☐	☐	☐
5. Was the intervention(s) or treatment procedure(s) clearly described?	☑	☐	☐	☐
6. Was the post-intervention clinical condition clearly described?	☑	☐	☐	☐
7. Were adverse events (harms) or unanticipated events identified and described?	☐	☐	☑	☐
8. Does the case report provide takeaway lessons?	☑	☐	☐	☐

Overall appraisal: Include ☑ Exclude ☐ Seek further info ☐

Comments (Including reason for exclusion)

© JBI, 2020. All rights reserved. JBI grants use of these tools for research purposes only. All other enquiries should be sent to jbisynthesis@adelaide.edu.au.

JBI CRITICAL APPRAISAL CHECKLIST FOR CASE CONTROL STUDIES

Reviewer______________________ Date 18.09.2020

Author Tan et al Year 2013 Record Number 8

	Yes	No	Unclear	Not applicable
1. Were the groups comparable other than the presence of disease in cases or the absence of disease in controls?	☐	☐	☐	☑
2. Were cases and controls matched appropriately?	☑	☐	☐	☐
3. Were the same criteria used for identification of cases and controls?	☑	☐	☐	☐
4. Was exposure measured in a standard, valid and reliable way?	☑	☐	☐	☐
5. Was exposure measured in the same way for cases and controls?	☑	☐	☐	☐
6. Were confounding factors identified?	☑	☐	☐	☐
7. Were strategies to deal with confounding factors stated?	☐	☐	☑	☐
8. Were outcomes assessed in a standard, valid and reliable way for cases and controls?	☑	☐	☐	☐
9. Was the exposure period of interest long enough to be meaningful?	☑	☐	☐	☐
10. Was appropriate statistical analysis used?	☑	☐	☐	☐

Overall appraisal: Include ☑ Exclude ☐ Seek further info ☐

Comments (Including reason for exclusion)

____

____

____

© JBI, 2020. All rights reserved. JBI grants use of these tools for research purposes only. All other enquiries should be sent to jbisynthesis@adelaide.edu.au.

JBI CRITICAL APPRAISAL CHECKLIST FOR QUASI-EXPERIMENTAL STUDIES

Reviewer_______________________ Date 19.09.2020

Author Xiao et al Year 2013 Record Number 9

	Yes	No	Unclear	Not applicable
1. Is it clear in the study what is the 'cause' and what is the 'effect' (i.e. there is no confusion about which variable comes first)?	☐	☐	☑	☐
2. Were the participants included in any comparisons similar?	☑	☐	☐	☐
3. Were the participants included in any comparisons receiving similar treatment/care, other than the exposure or intervention of interest?	☑	☐	☐	☐
4. Was there a control group?	☐	☐	☑	☐
5. Were there multiple measurements of the outcome both pre and post the intervention/exposure?	☑	☐	☐	☐
6. Was follow up complete and if not, were differences between groups in terms of their follow up adequately described and analyzed?	☐	☐	☐	☑
7. Were the outcomes of participants included in any comparisons measured in the same way?	☑	☐	☐	☐
8. Were outcomes measured in a reliable way?	☑	☐	☐	☐
9. Was appropriate statistical analysis used?	☑	☐	☐	☐

Overall appraisal: Include ☑ Exclude ☐ Seek further info ☐

Comments (Including reason for exclusion)

____

____

____

© JBI, 2020. All rights reserved. JBI grants use of these tools for research purposes only. All other enquiries should be sent to jbisynthesis@adelaide.edu.au.

JBI CRITICAL APPRAISAL CHECKLIST FOR COHORT STUDIES

Reviewer______________________ Date 17.09.2020

Author Seo et al Year 2011 Record Number 10

	Yes	No	Unclear	Not applicable
1. Were the two groups similar and recruited from the same population?	☑	☐	☐	☐
2. Were the exposures measured similarly to assign people	☑	☐	☐	☐
3. to both exposed and unexposed groups?				
4. Was the exposure measured in a valid and reliable way?	☑	☐	☐	☐
5. Were confounding factors identified?	☑	☐	☐	☐
6. Were strategies to deal with confounding factors stated?	☑	☐	☐	☐
7. Were the groups/participants free of the outcome at the start of the study (or at the moment of exposure)?	☐	☐	☑	☐
8. Were the outcomes measured in a valid and reliable way?	☑	☐	☐	☐
9. Was the follow up time reported and sufficient to be long enough for outcomes to occur?	☐	☐	☐	☑
10. Was follow up complete, and if not, were the reasons to loss to follow up described and explored?	☐	☐	☐	☑
11. Were strategies to address incomplete follow up utilized?	☐	☐	☐	☑
12. Was appropriate statistical analysis used?	☑	☐	☐	☐

Overall appraisal: Include ☑ Exclude ☐ Seek further info ☐

Comments (Including reason for exclusion)

____

____

© JBI, 2020. All rights reserved. JBI grants use of these tools for research purposes only. All other enquiries should be sent to jbisynthesis@adelaide.edu.au.

JBI CRITICAL APPRAISAL CHECKLIST FOR QUASI-EXPERIMENTAL STUDIES

Reviewer________________________ Date 19.09.2020

Author Delalleau et al Year 2011 Record Number 11

	Yes	No	Unclear	Not applicable
1. Is it clear in the study what is the 'cause' and what is the 'effect' (i.e. there is no confusion about which variable comes first)?	☑	☐	☐	☐
2. Were the participants included in any comparisons similar?	☑	☐	☐	☐
3. Were the participants included in any comparisons receiving similar treatment/care, other than the exposure or intervention of interest?	☑	☐	☐	☐
4. Was there a control group?	☐	☐	☑	☐
5. Were there multiple measurements of the outcome both pre and post the intervention/exposure?	☑	☐	☐	☐
6. Was follow up complete and if not, were differences between groups in terms of their follow up adequately described and analyzed?	☐	☐	☑	☐
7. Were the outcomes of participants included in any comparisons measured in the same way?	☑	☐	☐	☐
8. Were outcomes measured in a reliable way?	☑	☐	☐	☐
9. Was appropriate statistical analysis used?	☐	☐	☐	☑

Overall appraisal: Include ☑ Exclude ☐ Seek further info ☐

Comments (Including reason for exclusion)

____

____

____

© JBI, 2020. All rights reserved. JBI grants use of these tools for research purposes only. All other enquiries should be sent to jbisynthesis@adelaide.edu.au.

JBI CRITICAL APPRAISAL CHECKLIST FOR CASE REPORTS

Reviewer______________________ Date 21.09.2020

Author Jain et al Year 2010 Record Number 12

	Yes	No	Unclear	Not applicable
1. Were patient's demographic characteristics clearly described?	☑	☐	☐	☐
2. Was the patient's history clearly described and presented as a timeline?	☑	☐	☐	☐
3. Was the current clinical condition of the patient on presentation clearly described?	☑	☐	☐	☐
4. Were diagnostic tests or assessment methods and the results clearly described?	☑	☐	☐	☐
5. Was the intervention(s) or treatment procedure(s) clearly described?	☑	☐	☐	☐
6. Was the post-intervention clinical condition clearly described?	☑	☐	☐	☐
7. Were adverse events (harms) or unanticipated events identified and described?	☐	☐	☑	☐
8. Does the case report provide takeaway lessons?	☑	☐	☐	☐

Overall appraisal: Include ☑ Exclude ☐ Seek further info ☐

Comments (Including reason for exclusion)

____

____

____

© JBI, 2020. All rights reserved. JBI grants use of these tools for research purposes only. All other enquiries should be sent to jbisynthesis@adelaide.edu.au.

JBI CRITICAL APPRAISAL CHECKLIST FOR QUASI-EXPERIMENTAL STUDIES

Reviewer____________________ Date 20.09.2020

Author Pladellorens et al Year 2008 Record Number 13

	Yes	No	Unclear	Not applicable
1. Is it clear in the study what is the 'cause' and what is the 'effect' (i.e. there is no confusion about which variable comes first)?	☐	☐	☑	☐
2. Were the participants included in any comparisons similar?	☑	☐	☐	☐
3. Were the participants included in any comparisons receiving similar treatment/care, other than the exposure or intervention of interest?	☐	☐	☑	☐
4. Was there a control group?	☐	☐	☑	☐
5. Were there multiple measurements of the outcome both pre and post the intervention/exposure?	☐	☐	☑	☐
6. Was follow up complete and if not, were differences between groups in terms of their follow up adequately described and analyzed?	☐	☐	☑	☐
7. Were the outcomes of participants included in any comparisons measured in the same way?	☑	☐	☐	☐
8. Were outcomes measured in a reliable way?	☑	☐	☐	☐
9. Was appropriate statistical analysis used?	☐	☐	☐	☑

Overall appraisal: Include ☑ Exclude ☐ Seek further info ☐

Comments (Including reason for exclusion)

____

____

____

© JBI, 2020. All rights reserved. JBI grants use of these tools for research purposes only. All other enquiries should be sent to jbisynthesis@adelaide.edu.au.

JBI CRITICAL APPRAISAL CHECKLIST FOR QUASI-EXPERIMENTAL STUDIES

Reviewer_______________________ Date 18.09.2020

Author Tao et al Year 2003 Record Number 14

	Yes	No	Unclear	Not applicable
1. Is it clear in the study what is the 'cause' and what is the 'effect' (i.e. there is no confusion about which variable comes first)?	☐	☐	☑	☐
2. Were the participants included in any comparisons similar?	☑	☐	☐	☐
3. Were the participants included in any comparisons receiving similar treatment/care, other than the exposure or intervention of interest?	☐	☐	☑	☐
4. Was there a control group?	☐	☑	☐	☐
5. Were there multiple measurements of the outcome both pre and post the intervention/exposure?	☑	☐	☐	☐
6. Was follow up complete and if not, were differences between groups in terms of their follow up adequately described and analyzed?	☐	☐	☐	☑
7. Were the outcomes of participants included in any comparisons measured in the same way?	☑	☐	☐	☐
8. Were outcomes measured in a reliable way?	☑	☐	☐	☐
9. Was appropriate statistical analysis used?	☐	☐	☐	☐

Overall appraisal: Include ☐ Exclude ☐ Seek further info ☐

Comments (Including reason for exclusion)

___

___

___

© JBI, 2020. All rights reserved. JBI grants use of these tools for research purposes only. All other enquiries should be sent to jbisynthesis@adelaide.edu.au.

Periodontics and Oral Pathology

JBI CRITICAL APPRAISAL CHECKLIST FOR QUASI-EXPERIMENTAL STUDIES

Reviewer________________________ Date 29.10.2020

Author Ghanoum et al. Year 2019 Record Number 1

	Yes	No	Unclear	Not applicable
1. Is it clear in the study what is the 'cause' and what is the 'effect' (i.e. there is no confusion about which variable comes first)?	☐	☐	☑	☐
2. Were the participants included in any comparisons similar?	☑	☐	☐	☐
3. Were the participants included in any comparisons receiving similar treatment/care, other than the exposure or intervention of interest?	☐	☐	☑	☐
4. Was there a control group?	☐	☑	☐	☐
5. Were there multiple measurements of the outcome both pre and post the intervention/exposure?	☐	☑	☐	☐
6. Was follow up complete and if not, were differences between groups in terms of their follow up adequately described and analyzed?	☐	☐	☐	☑
7. Were the outcomes of participants included in any comparisons measured in the same way?	☑	☐	☐	☐
8. Were outcomes measured in a reliable way?	☑	☐	☐	☐
9. Was appropriate statistical analysis used?	☐	☐	☑	☐

Overall appraisal: Include ☑ Exclude ☐ Seek further info ☐

Comments (Including reason for exclusion)

____

____

____

© JBI, 2020. All rights reserved. JBI grants use of these tools for research purposes only. All other enquiries should be sent to jbisynthesis@adelaide.edu.au.

JBI CRITICAL APPRAISAL CHECKLIST FOR COHORT STUDIES

Reviewer________________________ Date 30.10.2020

Author Mayer et al Year 2017 Record Number 2

		Yes	No	Unclear	Not applicable
1.	Were the two groups similar and recruited from the same population?	☒	☐	☐	☐
2.	Were the exposures measured similarly to assign people to both exposed and unexposed groups?	☒	☐	☐	☐
3.	Was the exposure measured in a valid and reliable way?	☒	☐	☐	☐
4.	Were confounding factors identified?	☐	☒	☐	☐
5.	Were strategies to deal with confounding factors stated?	☐	☒	☐	☐
6.	Were the groups/participants free of the outcome at the start of the study (or at the moment of exposure)?	☒	☐	☐	☐
7.	Were the outcomes measured in a valid and reliable way?	☒	☐	☐	☐
8.	Was the follow up time reported and sufficient to be long enough for outcomes to occur?	☒	☐	☐	☐
9.	Was follow up complete, and if not, were the reasons to loss to follow up described and explored?	☐	☐	☒	☐
10.	Were strategies to address incomplete follow up utilized?	☐	☐	☒	☐
11.	Was appropriate statistical analysis used?	☒	☐	☐	☐

Overall appraisal: Include ☒ Exclude ☐ Seek further info ☐

Comments (Including reason for exclusion)

____

____

© JBI, 2020. All rights reserved. JBI grants use of these tools for research purposes only. All other enquiries should be sent to jbisynthesis@adelaide.edu.au.

JBI CRITICAL APPRAISAL CHECKLIST FOR QUASI-EXPERIMENTAL STUDIES

Reviewer________________________ Date 6.11.20

Author Iwami et al Year 2007 Record Number 3

	Yes	No	Unclear	Not applicable
1. Is it clear in the study what is the 'cause' and what is the 'effect' (i.e. there is no confusion about which variable comes first)?	☑	☐	☐	☐
2. Were the participants included in any comparisons similar?	☑	☐	☐	☐
3. Were the participants included in any comparisons receiving similar treatment/care, other than the exposure or intervention of interest?	☑	☐	☐	☐
4. Was there a control group?	☐	☐	☑	☐
5. Were there multiple measurements of the outcome both pre and post the intervention/exposure?	☐	☐	☑	☐
6. Was follow up complete and if not, were differences between groups in terms of their follow up adequately described and analyzed?	☐	☐	☐	☑
7. Were the outcomes of participants included in any comparisons measured in the same way?	☑	☐	☐	☐
8. Were outcomes measured in a reliable way?	☐	☑	☐	☐
9. Was appropriate statistical analysis used?	☑	☐	☐	☐

Overall appraisal: Include ☑ Exclude ☐ Seek further info ☐

Comments (Including reason for exclusion)

____

____

____

© JBI, 2020. All rights reserved. JBI grants use of these tools for research purposes only. All other enquiries should be sent to jbisynthesis@adelaide.edu.au.

JBI CRITICAL APPRAISAL CHECKLIST FOR QUASI-EXPERIMENTAL STUDIES

Reviewer__________ Date 6.11.20

Author Berdouses et al Year 2015 Record Number__________

4

	Yes	No	Unclear	Not applicable
1. Is it clear in the study what is the 'cause' and what is the 'effect' (i.e. there is no confusion about which variable comes first)?	☑	☐	☐	☐
2. Were the participants included in any comparisons similar?	☐	☐	☑	☐
3. Were the participants included in any comparisons receiving similar treatment/care, other than the exposure or intervention of interest?	☑	☐	☐	☐
4. Was there a control group?	☐	☑	☐	☐
5. Were there multiple measurements of the outcome both pre and post the intervention/exposure?	☐	☐	☑	☐
6. Was follow up complete and if not, were differences between groups in terms of their follow up adequately described and analyzed?	☐	☐	☐	☑
7. Were the outcomes of participants included in any comparisons measured in the same way?	☑	☐	☐	☐
8. Were outcomes measured in a reliable way?	☑	☐	☐	☐
9. Was appropriate statistical analysis used?	☐	☐	☑	☐

Overall appraisal: Include ☑ Exclude ☐ Seek further info ☐

Comments (Including reason for exclusion)

© JBI, 2020. All rights reserved. JBI grants use of these tools for research purposes only. All other enquiries should be sent to jbisynthesis@adelaide.edu.au.

JBI CRITICAL APPRAISAL CHECKLIST FOR COHORT STUDIES

Reviewer_______________________ Date 30.10.2020

Author Torlakovic et al Year 2012 Record Number 5

	Yes	No	Unclear	Not applicable
1. Were the two groups similar and recruited from the same population?	☑	☐	☐	☐
2. Were the exposures measured similarly to assign people to both exposed and unexposed groups?	☑	☐	☐	☐
3. Was the exposure measured in a valid and reliable way?	☐	☐	☑	☐
4. Were confounding factors identified?	☑	☐	☐	☐
5. Were strategies to deal with confounding factors stated?	☐	☑	☐	☐
6. Were the groups/participants free of the outcome at the start of the study (or at the moment of exposure)?	☑	☐	☐	☐
7. Were the outcomes measured in a valid and reliable way?	☑	☐	☐	☐
8. Was the follow up time reported and sufficient to be long enough for outcomes to occur?	☐	☐	☐	☑
9. Was follow up complete, and if not, were the reasons to loss to follow up described and explored?	☐	☐	☐	☑
10. Were strategies to address incomplete follow up utilized?	☐	☐	☐	☑
11. Was appropriate statistical analysis used?	☑	☐	☐	☐

Overall appraisal: Include ☑ Exclude ☐ Seek further info ☐

Comments (Including reason for exclusion)

____

____

© JBI, 2020. All rights reserved. JBI grants use of these tools for research purposes only. All other enquiries should be sent to jbisynthesis@adelaide.edu.au.

Orthodontics

JBI CRITICAL APPRAISAL CHECKLIST FOR QUASI-EXPERIMENTAL STUDIES

Reviewer____________________ Date 15.09.2020

Author Berssenbrügge et al Year 2015 Record Number 1

	Yes	No	Unclear	Not applicable
1. Is it clear in the study what is the 'cause' and what is the 'effect' (i.e. there is no confusion about which variable comes first)?	☑	☐	☐	☐
2. Were the participants included in any comparisons similar?	☑	☐	☐	☐
3. Were the participants included in any comparisons receiving similar treatment/care, other than the exposure or intervention of interest?	☑	☐	☐	☐
4. Was there a control group?	☐	☑	☐	☐
5. Were there multiple measurements of the outcome both pre and post the intervention/exposure?	☐	☐	☑	☐
6. Was follow up complete and if not, were differences between groups in terms of their follow up adequately described and analyzed?	☐	☐	☑	☐
7. Were the outcomes of participants included in any comparisons measured in the same way?	☑	☐	☐	☐
8. Were outcomes measured in a reliable way?	☐	☑	☐	☐
9. Was appropriate statistical analysis used?	☑	☐	☐	☐

Overall appraisal: Include ☑ Exclude ☐ Seek further info ☐

Comments (Including reason for exclusion)

____

____

____

© JBI, 2020. All rights reserved. JBI grants use of these tools for research purposes only. All other enquiries should be sent to jbisynthesis@adelaide.edu.au.

Article title: How Accurate Are the Fusion of Cone-Beam CT and 3-D Stereophotographic Images?

JBI CRITICAL APPRAISAL CHECKLIST FOR COHORT STUDIES

Reviewer________________________ Date 16.09.2020

Author Jayaratne et al Year 2012 Record Number 2

	Yes	No	Unclear	Not applicable
1. Were the two groups similar and recruited from the same population?	☑	☐	☐	☐
2. Were the exposures measured similarly to assign people<br>3. to both exposed and unexposed groups?	☑	☐	☐	☐
4. Was the exposure measured in a valid and reliable way?	☑	☐	☐	☐
5. Were confounding factors identified?	☑	☐	☐	☐
6. Were strategies to deal with confounding factors stated?	☑	☐	☐	☐
7. Were the groups/participants free of the outcome at the start of the study (or at the moment of exposure)?	☐	☐	☑	☐
8. Were the outcomes measured in a valid and reliable way?	☐	☐	☑	☐
9. Was the follow up time reported and sufficient to be long enough for outcomes to occur?	☐	☐	☑	☐
10. Was follow up complete, and if not, were the reasons to loss to follow up described and explored?	☐	☐	☑	☐
11. Were strategies to address incomplete follow up utilized?	☐	☐	☑	☐
12. Was appropriate statistical analysis used?	☑	☐	☐	☐

Overall appraisal: Include ☑ Exclude ☐ Seek further info ☐

Comments (Including reason for exclusion)

© JBI, 2020. All rights reserved. JBI grants use of these tools for research purposes only. All other enquiries should be sent to jbisynthesis@adelaide.edu.au.

Article title: Three-Dimensional Color Maps: A Novel Tool for Assessing Craniofacial Changes

JBI Critical Appraisal Checklist for Case Series

Reviewer_______________________ Date 15.09.2020

Author Jayaratne et al. Year 2010 Record Number 3

	Yes	No	Unclear	Not applicable
• Were there clear criteria for inclusion in the case series?	☐	☑	☐	☐
• Was the condition measured in a standard, reliable way for all participants included in the case series?	☑	☐	☐	☐
• Were valid methods used for identification of the condition for all participants included in the case series?	☑	☐	☐	☐
• Did the case series have consecutive inclusion of participants?	☐	☐	☑	☐
• Did the case series have complete inclusion of participants?	☐	☐	☑	☐
• Was there clear reporting of the demographics of the participants in the study?	☑	☐	☐	☐
• Was there clear reporting of clinical information of the participants?	☑	☐	☐	☐
• Were the outcomes or follow up results of cases clearly reported?	☐	☑	☐	☐
• Was there clear reporting of the presenting site(s)/clinic(s) demographic information?	☐	☐	☑	☐
• Was statistical analysis appropriate?	☐	☐	☐	☑

Overall appraisal: Include ☑ Exclude ☐ Seek further info ☐

Comments (Including reason for exclusion)

____

____

© JBI, 2020. All rights reserved. JBI grants use of these tools for research purposes only. All other enquiries should be sent to jbisynthesis@adelaide.edu.au.

JBI CRITICAL APPRAISAL CHECKLIST FOR COHORT STUDIES

Reviewer____________________ Date 17.09.2020

Author Brough et al Year 2010 Record Number 4

	Yes	No	Unclear	Not applicable
1. Were the two groups similar and recruited from the same population?	☑	☐	☐	☐
2. Were the exposures measured similarly to assign people	☑	☐	☐	☐
3. to both exposed and unexposed groups?				
4. Was the exposure measured in a valid and reliable way?	☑	☐	☐	☐
5. Were confounding factors identified?	☑	☐	☐	☐
6. Were strategies to deal with confounding factors stated?	☐	☐	☑	☐
7. Were the groups/participants free of the outcome at the start of the study (or at the moment of exposure)?	☑	☐	☐	☐
8. Were the outcomes measured in a valid and reliable way?	☐	☑	☐	☐
9. Was the follow up time reported and sufficient to be long enough for outcomes to occur?	☐	☐	☐	☑
10. Was follow up complete, and if not, were the reasons to loss to follow up described and explored?	☐	☐	☐	☑
11. Were strategies to address incomplete follow up utilized?	☐	☐	☐	☑
12. Was appropriate statistical analysis used?	☑	☐	☐	☐

Overall appraisal: Include ☑ Exclude ☐ Seek further info ☐

Comments (Including reason for exclusion)

____

____

© JBI, 2020. All rights reserved. JBI grants use of these tools for research purposes only. All other enquiries should be sent to jbisynthesis@adelaide.edu.au.
